# Supplementary material for: How to Teach Cross-Cultural Communication: A Workshop Using the Experiential Learning Model
Source: MedEdPORTAL. 2023 Nov 21;19:11365. doi: 10.15766/mep_2374-8265.11365 (PMC10662213; doi:10.15766/mep_2374-8265.11365)
Supplement: Supplementary file 1 — Participant Handout.docxFacilitator Guide.docxSlide Presentation.pptxRetrospective Pre-Post Survey.docx3-Month Postworkshop Survey.docx [file mep_2374-8265.11365-s001.zip › D. Retrospective Pre-Post Survey.docx]

Workshop Evaluation

Please complete the survey below. Thank you!

Please create a unique identifier as follows: first 4 letters of childhood street name AND year of high school graduation.

*For example, if childhood street name is Telegraph Road and year of graduation is 2006, unique identifier would be tele2006.*

Unique Identifier: __________________________________________

**How satisfied were you with the**

|  | Not at all satisfied | Slightly satisfied | Moderately satisfied | Quite satisfied | Extremely satisfied |
| --- | --- | --- | --- | --- | --- |
| overall structure of the workshop? | 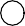 | 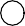 | 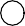 | 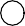 | 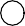 |
| main facilitator of the workshop? | 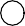 | 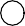 | 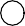 | 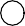 | 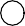 |

**How useful did you find the following parts of the training?**

|  | Not at all useful | Slightly useful | Moderately useful | Quite useful | Extremely useful |
| --- | --- | --- | --- | --- | --- |
| didactic session reviewing definitions? | 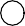 | 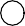 | 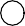 | 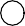 | 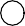 |
| small group self-reflection? | 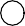 | 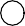 | 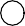 | 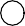 | 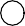 |
| large group discussion of Lia  Lee's case? | 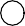 | 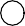 | 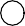 | 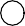 | 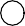 |
| didactic session reviewing  Kleinman's 8 questions and  LEARN model? | 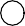 | 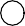 | 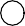 | 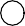 | 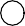 |
| video demonstrating use of  Kleinman's 8 questions and  LEARN model? | 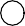 | 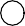 | 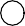 | 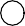 | 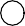 |
| Small group exercise  re-imagining Lia Lee's case  through cross-cultural  communication models? | 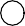 | 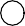 | 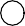 | 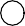 | 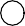 |

| Overall, how successful was this workshop in addressing its stated objectives? | - Not at all successful - Slightly successful - Moderately successful - Quite successful - Extremely successful |
| --- | --- |
| What is the one key takeaway you took from the  workshop? |  |
| If you were to change anything about this workshop,  what would it be? |  |

| **Please answer the following questions based on your familiarity/awareness/confidence BEFORE attending this workshop.** | | |
| --- | --- | --- |
| **Before this workshop,** |  |  |

| How familiar were you with Kleinman's 8 questions? | - Not at all familiar - Slightly familiar - Moderately familiar - Quite familiar - Extremely familiar |
| --- | --- |
| How familiar were you with the LEARN model of cross-cultural communication? | - Not at all familiar - Slightly familiar - Moderately familiar - Quite familiar - Extremely familiar |
| How aware were you of the impact of your own cultural identity on your approach to medicine? | - Not at all aware - Slightly aware - Moderately aware - Quite aware - Extremely aware |
| How aware were you of the impact of your own cultural identity on cross-cultural communication? | - Not at all aware - Slightly aware - Moderately aware - Quite aware - Extremely aware |
| How important did you feel understanding a patient's cultural identity was to effective cross-cultural communication? | - Not at all important - Slightly important - Moderately important - Quite important - Extremely important |
| How aware were you of the impact of Western medical culture on your own illness explanatory model? | - Not at all aware - Slightly aware - Moderately aware - Quite aware - Extremely aware |

**How confident did you feel**

|  | Not at all confident | Slightly confident | Moderately confident | Quite confident | Extremely confident |
| --- | --- | --- | --- | --- | --- |
| communicating with  patients/families with different cultural beliefs? | 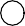 | 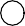 | 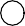 | 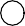 | 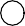 |
| eliciting different health  perspectives of patients and  their families? | 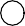 | 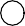 | 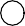 | 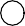 | 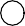 |
| managing cross-cultural  misunderstandings regarding  the work up for a diagnosis in  the inpatient setting? | 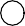 | 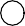 | 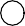 | 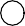 | 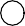 |
| managing cross-cultural  misunderstandings when  conveying a diagnosis in the  inpatient setting? | 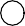 | 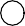 | 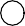 | 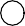 | 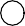 |
| managing cross-cultural  misunderstandings when  explaining disease management in the inpatient setting? | 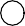 | 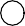 | 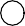 | 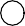 | 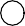 |

| **Please answer the following questions based on your familiarly/awareness/confidence AFTER**  **attending this workshop.** | | |
| --- | --- | --- |
| **After this workshop,** |  |  |

| How familiar are you with Kleinman's 8 questions? | - Not at all familiar - Slightly familiar - Moderately familiar - Quite familiar - Extremely familiar |
| --- | --- |
| How familiar are you with the LEARN model of cross-cultural communication? | - Not at all familiar - Slightly familiar - Moderately familiar - Quite familiar - Extremely familiar |
| How aware are you of the impact of your own cultural identity on your approach to medicine? | - Not at all aware - Slightly aware - Moderately aware - Quite aware - Extremely aware |
| How aware are you of the impact of your own cultural identity on cross-cultural communication? | - Not at all aware - Slightly aware - Moderately aware - Quite aware - Extremely aware |
| How important do you feel understanding a patient's cultural identity is to effective cross-cultural communication? | - Not at all important - Slightly important - Moderately important - Quite important - Extremely important |
| How aware are you of the impact of Western medical culture on your own illness explanatory model? | - Not at all aware - Slightly aware - Moderately aware - Quite aware - Extremely aware |

**How confident do you feel**

|  | Not at all confident | Slightly confident | Moderately confident | Quite confident | Extremely confident |
| --- | --- | --- | --- | --- | --- |
| communicating with  patients/families with different cultural beliefs? | 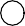 | 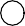 | 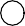 | 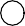 | 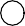 |
| eliciting different health  perspectives of patients and  their families? | 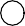 | 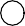 | 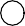 | 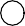 | 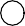 |
| managing cross-cultural  misunderstandings regarding  the work up for a diagnosis in  the inpatient setting? | 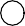 | 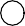 | 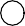 | 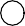 | 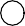 |
| managing cross-cultural  misunderstandings when  conveying a diagnosis in the  inpatient setting? | 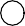 | 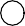 | 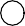 | 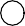 | 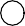 |
| managing cross-cultural  misunderstandings when  explaining disease management in the inpatient setting? | 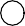 | 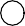 | 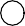 | 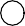 | 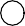 |
| using Kleinman's 8 questions to better understand a family's illness explanatory model? | 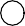 | 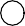 | 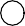 | 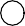 | 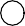 |
| using the LEARN model to come to an agreement with a  patient/parent regarding diagnostic and/or treatment  plan? | 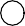 | 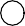 | 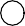 | 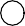 | 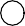 |

**How frequently**

|  | Never | Rarely | Sometimes | Often | Always |
| --- | --- | --- | --- | --- | --- |
| did cultural and/or spiritual  beliefs play a significant role in your family's approach to  healthcare? |  |  |  |  |  |
| do cultural and/or spiritual  beliefs play a significant role in your current approach to  healthcare? |  |  |  |  |  |

**Pre-Workshop Exposure**

| Have you ever participated in a curriculum or workshop focused on cross-cultural communication? | - Yes - No - I don’t know |
| --- | --- |
| Please specify in what context you participated in  this curriculum/workshop. |  |
| Did you participate in any specific tracks focused on underserved health during medical school? | - Yes - No - I don’t know |
| Please specify the track you participated in during  medical school. |  |

**Demographics**

| What is your postgraduate level? | - PGY-1 - PGY-2 - PGY-3 - PGY-4 - PGY-5 - Other- please specify |
| --- | --- |
| Other: please specify. |  |
| Please choose the description that is most applicable to you. | - Categorical Pediatric Resident - Internal Medicine-Pediatrics Resident - Triple Board Pediatrics, Adult Psychiatry, Child and Adolescent Psychiatry Resident OR Combined Pediatric and Medical Genetics Resident - Other- please specify. |
| Other: please specify. |  |
| To which gender identity do you most identify? | - Female - Male - Transgender Female - Transgender Male - Gender Variant/Non-conforming - Other- Please specify - Prefer not to answer |
| Other: please specify. |  |
| Please indicate your ethnicity. | - Hispanic or Latino - Not Hispanic or Latino - Prefer not to answer |
| Please indicate your race. | - White - Black/African American - Asian - Native American/Alaskan Native - Native Hawaiian/Other Pacific Islander - Other- please specify - Prefer not to answer |
| Other: please specify. |  |
| What is your generational status? Note, 1st generation means you were born in a different country, 2nd generation means you have at least one foreign-born parent, and 3rd and higher generation means you have two U.S. native parents. | - 1st generation - 2nd generation - 3rd or higher generation - I don't know |
| Have you lived in a country outside of the United States for any portion of your childhood? | - Yes - No |
| How many years did you live outside of the United States? | - Less than 1 year - 1-5 years - 5-10 years - Longer than 10 years |
| The time I spent living outside the United States was | - Within the last 5 years - Longer than 5 years ago |
| Where did you complete medical school? | - United States - Outside of the United States |
| Did you complete clinical rotations/observerships/externships in the United States while in medical school? | - Yes - No |
| Have you completed a residency program outside of the United States? | - Yes - No |
