# Supplementary material for: How to Teach Cross-Cultural Communication: A Workshop Using the Experiential Learning Model
Source: MedEdPORTAL. 2023 Nov 21;19:11365. doi: 10.15766/mep_2374-8265.11365 (PMC10662213; doi:10.15766/mep_2374-8265.11365)
Supplement: Supplementary file 1 — Participant Handout.docxFacilitator Guide.docxSlide Presentation.pptxRetrospective Pre-Post Survey.docx3-Month Postworkshop Survey.docx [file mep_2374-8265.11365-s001.zip › E. 3-Month Postworkshop Survey.docx]

Post workshop 3-month survey

Please complete the survey below.

Thank you!

| 1. Please create a unique identifier as follows: first 4 letters of childhood street name AND year of high school graduation. Please use the same unique identifier as you did for the first survey.   *For example, if childhood street name is Telegraph Road and year of graduation is 2006, unique identifier would be tele2006.* | Unique Identifier: |
| --- | --- |
| 1. How familiar are you with Kleinman's 8 questions? | - Not at all familiar - Slightly familiar - Moderately familiar - Quite familiar - Extremely familiar |
| 1. How familiar are you with the LEARN model of cross-cultural communication? | - Not at all familiar - Slightly familiar - Moderately familiar - Quite familiar - Extremely familiar |
| 1. How aware are you of the impact of your own cultural identity on your approach to medicine? | - Not at all aware - Slightly aware - Moderately aware - Quite aware - Extremely aware |
| 1. How aware are you of the impact of your own cultural identity on cross-cultural communication? | - Not at all aware - Slightly aware - Moderately aware - Quite aware - Extremely aware |
| 1. How important do you feel understanding a patient's cultural identity is to effective cross-cultural communication? | - Not at all important - Slightly important - Moderately important - Quite important - Extremely important |
| 1. How aware are you of the impact of Western medical culture on your own illness explanatory model? | - Not at all aware - Slightly aware - Moderately aware - Quite aware - Extremely aware |

**How confident do you feel**

|  | Not at all confident | Slightly confident | Moderately confident | Quite confident | Extremely confident |
| --- | --- | --- | --- | --- | --- |
| 1. communicating with patients/families with different cultural beliefs? | 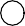 | 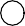 | 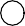 | 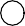 | 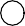 |
| 1. eliciting different health perspectives of patients and their families? | 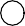 | 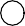 | 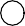 | 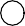 | 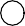 |
| 1. managing cross-cultural misunderstandings regarding the work-up for a diagnosis in the inpatient setting? | 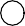 | 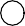 | 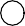 | 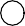 | 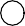 |
| 1. managing cross-cultural misunderstandings when conveying a diagnosis in the inpatient setting? | 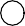 | 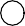 | 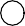 | 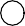 | 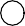 |
| 1. managing cross-cultural misunderstandings when explaining disease management in the inpatient setting? | 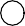 | 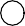 | 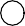 | 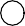 | 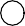 |
| 1. using Kleinman's 8 questions to better understand a family's illness explanatory model? | 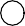 | 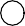 | 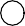 | 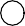 | 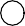 |
| 1. using the LEARN model to come to an agreement with a patient/parent regarding diagnostic and/or treatment plan? | 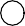 | 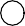 | 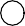 | 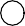 | 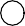 |

**Over the past 3 months, in what percentage of patient encounters have you**

|  | 0-25% | 25-50% | 50-75% | 75-100% |
| --- | --- | --- | --- | --- |
| 1. been exposed to a patient of different culture than your own? | 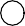 | 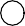 | 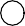 | 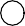 |
| 1. used the Kleinman model to facilitate cross-cultural communication when appropriate? | 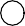 | 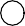 | 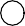 | 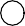 |
| 1. used the LEARN model to facilitate cross-cultural communication when appropriate? | 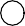 | 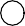 | 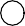 | 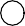 |

| 1. How likely are you to use these cross-cultural communication models in the future while on the inpatient floors? | - Very likely - Likely - Unlikely - Very unlikely |
| --- | --- |
| 1. How valuable has the Kleinman 8 question model been in guiding cross-cultural communication with patients and families in the inpatient setting? | - Not at all valuable - Slightly valuable - Moderately valuable - Quite valuable - Extremely valuable |
| 1. How valuable has the LEARN model been in guiding cross-cultural communication with patients and families in the inpatient setting? | - Not at all valuable - Slightly valuable - Moderately valuable - Quite valuable - Extremely valuable |
| 1. Did you read the email reminders that were sent out regarding cross-cultural communication? | - Yes - No - I don’t know - Prefer not to answer |
