# Supplementary material for: How to Teach Cross-Cultural Communication: A Workshop Using the Experiential Learning Model
Source: MedEdPORTAL. 2023 Nov 21;19:11365. doi: 10.15766/mep_2374-8265.11365 (PMC10662213; doi:10.15766/mep_2374-8265.11365)
Supplement: Supplementary file 1 — Participant Handout.docxFacilitator Guide.docxSlide Presentation.pptxRetrospective Pre-Post Survey.docx3-Month Postworkshop Survey.docx [file mep_2374-8265.11365-s001.zip › A. Participant Handout.docx]

**Appendix A: Participant Handout**

**This appendix is intended to be used by participants to follow along with the slides of Appendix C and to have a written copy of self-discussion prompts, cross-cultural communication models, and the Lia Lee case on hard (either electronic or hard copy) to reference. If distributing electronically, the facilitator can either email the appendix out to participants prior to the workshop, present a QR code on relevant slides (3, 17, and 36) that participants can scan to have access to the appendix in real time on their electronic device. An easy way to do this is to generate a QR code to a google drive folder where the document is housed. To do this, create a folder on google drive that is public. Then, generate a QR code that leads to this link by opening the document in Google Chrome, right clicking the Share button, and selecting “Create QR code for this page.” You can then download the QR code and paste it onto this slide.**

**Objectives**

By the end of this activity, learners will be able to:

1. Identify key elements of one’s own cultural identity and illness explanatory models through guided self-reflection.
2. Recognize the impact of western medical culture on our own illness explanatory models as physicians.
3. Explain the impact of cultural beliefs and values of both the provider and patient/parent on patient physician communication.
4. Demonstrate use of cross-cultural communication models (i.e., LEARN model, Kleinman’s 8 questions) in small groups and with peers to build skills for culturally challenging communication situations.
5. Apply cross-cultural communication models in clinical settings to adequately explore a patient’s or parent’s perspective on illness, including their understanding and fears regarding work-up, diagnosis, and treatment, and to form a therapeutic alliance between the medical team and patient/family.

**Part 1- Definitions and Self-Reflection Prompts**

**Definitions**

What is culture?

- Culture is a difficult word to define. According to Boutin-Foster, et. Al, “Culture refers to integrated patterns of human behavior that include language, thoughts, communications, actions, beliefs, values, and institutions of racial, ethnic, religious, or social groups.”^1^
- Most importantly, culture is not static, it is fluid. Culture can change with big life events, including becoming a parent, the death of a loved one, or experiencing our own health problems. It is important to self-reflect on these experiences from time to time to become familiar with how they affect our own cultural identities and how we see the world.
- Additionally, medicine has its own culture which we are all a part of. We have been informed by our medical school curriculum as well as peers and mentors. This affects how we see different symptoms and disease entities.

What is cultural humility?

- Most of you are familiar with the term cultural competency. Cultural humility, however, is different. Instead of focusing on understanding certain cultures and other groups by becoming familiar with a set of beliefs, values, and traditions they have in common, it focuses on getting to know the individual themselves. This requires you to constantly engage in self-reflection about your own culture in order to better understand others.^2, 3^
- Cultural humility demands understanding that culture is multifactorial, dynamic, fluid, and INDIVIDUAL.
- This is a lifelong process.

What is an illness explanatory model?

- Simply put, an illness explanatory model is how the patient explains his or her illness. More specifically, this model is how the patient understands what is causing their illness, why they feel the symptoms that they feel, and what they believe is necessary to treat their illness. Sometimes, this model matches ours as the medical team, and sometimes, our own model differs from our patients. Eliciting the patient’s illness explanatory model allows us to better understand their point of view and helps us approach the conversation regarding next steps with empathy and respect. ^4, 5^

**Exploring your own cultural identity**

Please reflect on the following prompts and questions. Be ready to discuss your thoughts in small groups. Feel free to use the spaces below under each prompt to write down your thoughts.

1. List 5 (or more!) components of your cultural identity.
2. How do these components, both separate and combined, affect how you see the world? How do they affect your illness explanatory model(s)?
3. Have you ever felt misunderstood due to your cultural beliefs/values? Have you ever been in or witnessed a patient encounter where there seemed to be a misunderstanding between the medical team and family due to different cultural beliefs/values?

*Reflection activity adapted from My Multicultural Self courtesy of the Southern Poverty Law Center

**Part 2- Models of Cross-Cultural Communication**

**Kleinman’s 8 Questions**^6^

What do you think has caused your problem?

Why do you think it started when it did?

What do you think your sickness does to you? How does it work?

How severe is your sickness? Will it have a short or long course?

What kind of treatment do you think you should receive?

What are the most important results you hope to receive from this treatment?

What are the chief problems your sickness has caused for you?

What do you fear most about your sickness?

**LEARN Model**^7^

L: ***Listen*** with sympathy and understanding to the patient’s perception of the problem

E: ***Explain*** your perceptions of the problem

A: ***Acknowledge*** and discuss the differences and similarities

R: ***Recommend*** treatment

N: ***Negotiate*** treatment

**Negotiation Tips**^8^

Describe diagnostic and treatment options in terms that are understandable to the patient and patient’s family

Determine the patient’s priorities.

Present a reasonable management plan- try to prioritize management if possible.

Determine patient and family’s level of acceptance of this plan by directly asking.

Conflict may remain. If so, focus on higher priorities.

**Part 3- Lia Lee Small Group Exercise**

Feel free to use the spaces below under each prompt to write down your thoughts.

**Group 1: L:** What questions would you ask to explore the parent’s understanding of Lia Lee’s condition and their understanding of the current treatment plan?

**Group 2: E:** How would you explain your (physician) perception of Lia’s illness and treatment?

**Group 3: A:** How would you acknowledge the parents’ illness explanatory model in your conversation and discuss similarities and differences of their model with yours in a non-judgmental way that shows empathy, curiosity, and respect?

**Group 4: R:** How would you phrase your recommendations to Lia’s parents?

**Group 5: N:** How would you approach negotiating the differences between your illness explanatory model and that of Lia’s parents? How might you incorporate Lia’s family’s concerns and approaches to the illness in your treatment plan? What concerns do you have about this?

**References**

1. Boutin-Foster C, Foster JC, Konopasek L. Viewpoint: physician, know thyself: the professional culture of medicine as a framework for teaching cultural competence. Acad Med. 2008 Jan;83(1):106-11. Doi: 10.1097/ACM.0b013e31815c6753. PMID: 18162762.
2. Chang ES, Simon M, Dong X. Integrating cultural humility into health care professional education and training. Adv Health Sci Educ Theory Pract. 2012 May;17(2):269-78. Doi: 10.1007/s10459-010-9264-1. Epub 2010 Dec 16. PMID: 21161680.
3. Tervalon M, Murray-García J. Cultural humility versus cultural competence: a critical distinction in defining physician training outcomes in multicultural education. J Health Care Poor Underserved. 1998 May;9(2):117-25. Doi: 10.1353/hpu.2010.0233. PMID: 10073197.
4. Dinos, S., Ascoli, M., Owiti, J., & Bhui, K. (2017). Assessing explanatory models and health beliefs: An essential but overlooked competency for clinicians. *BJPsych Advances,* *23*(2), 106-114. Doi:10.1192/apt.bp.114.013680
5. Kleinman A, Eisenberg L, Good B. Culture, illness, and care: clinical lessons from anthropologic and cross-cultural research. Ann Intern Med. 1978 Feb;88(2):251-8. Doi: 10.7326/0003-4819-88-2-251. PMID: 626456.
6. Kleinman A, Eisenberg L, Good B. Culture, illness, and care: clinical lessons from anthropologic and cross-cultural research. Ann Intern Med. 1978 Feb;88(2):251-8. Doi: 10.7326/0003-4819-88-2-251. PMID: 626456.
7. Berlin EA, Fowkes WC Jr. A teaching framework for cross-cultural health care. Application in family practice. West J Med. 1983 Dec;139(6):934-8. PMID: 6666112; PMCID: PMC1011028.
8. Carrillo JE, Green AR, Betancourt JR. Cross-cultural primary care: a patient-based approach. Ann Intern Med. 1999 May 18;130(10):829-34. Doi: 10.7326/0003-4819-130-10-199905180-00017. PMID: 10366373.

Worksheet compiled by Angie Buttigieg, MD
