# Supplementary material for: How to Teach Cross-Cultural Communication: A Workshop Using the Experiential Learning Model
Source: MedEdPORTAL. 2023 Nov 21;19:11365. doi: 10.15766/mep_2374-8265.11365 (PMC10662213; doi:10.15766/mep_2374-8265.11365)
Supplement: Supplementary file 1 — Participant Handout.docxFacilitator Guide.docxSlide Presentation.pptxRetrospective Pre-Post Survey.docx3-Month Postworkshop Survey.docx [file mep_2374-8265.11365-s001.zip › B. Facilitator Guide.docx]

**Appendix B: Facilitator Guide**

**Resource Description**

This two-hour workshop is designed to enhance resident skills and comfort with cross-cultural patient encounters. The workshop is broken down into two parts, each one hour long. The first half of the workshop is focused on self-reflection. After a brief introduction to the workshop and to relevant terminology, participants will be split into small groups and given self-reflection prompts to discuss amongst the group. The goal of this half of the session is to have participants explore their own cultural identity and illness explanatory models and how these are both influenced by their upbringing, life events, and their training.

The second half of the workshop is focused on introducing and practicing two models of cross-cultural communication- the LEARN model, and Kleinman’s eight questions. This half of the workshop is introduced via an example of poor cross-cultural communication and discussion regarding what went wrong, and what could be improved. The LEARN model and Kleinman’s eight questions are then introduced. A case will be used in real time to demonstrate each letter of the LEARN model, then a video will be shown at the end to demonstrate the use of these models in their entirety. Finally, participants will have a chance to re-imagine the case of poor cross-cultural communication discussed at the beginning of the session in small groups.

**Objectives**

By the end of this activity, learners will be able to:

1. Identify key elements of one’s own cultural identity and illness explanatory models through guided self-reflection.
2. Recognize the impact of western medical culture on our own illness explanatory models as physicians.
3. Explain the impact of cultural beliefs and values of both the provider and patient/parent on patient physician communication.
4. Demonstrate use of cross-cultural communication models (i.e., LEARN model, Kleinman’s 8 questions) in small groups and with peers to build skills for culturally challenging communication situations.
5. Apply cross-cultural communication models in clinical settings to adequately explore a patient’s or parent’s perspective on illness, including their understanding and fears regarding work-up, diagnosis, and treatment, and to form a therapeutic alliance between the medical team and patient/family.

**Workshop Outline**

| **Part 1: Self-Reflection** | |
| --- | --- |
| **Workshop Segment** | **Time** |
| Explanation of workshop objectives (Slide 2) | 5 minutes |
| Didactic (Slides 3-7)   - What is culture, what is cultural humility? - transition slides explaining our own cultural identities, medical professional culture, and the impact of this on cross-cultural communication | 5 minutes |
| Small Group Reflection about participants’ own cultural identity/values in small groups (Breakout rooms or physical small groups) (Slide 8-9) | 8 minutes |
| Large Group Discussion to share multiple different aspects of cultural identity (Slide 10) | 5 minutes |
| Small Group Reflection about how participants’ own cultural identity/values shapes the way they see the world and medicine (Slide 11) | 8 minutes |
| Large Group Discussion to share how these components affect how we see the world. (Slide 12) | 5 minutes |
| Small Group Reflection about a time they felt misunderstood or were in a patient encounter in which the patient felt misunderstood due to different cultural beliefs/values (Slide 13) | 8 minutes |
| Large Group Discussion- 1-2 groups share examples (Slide 14) | 5 minutes |
| Wrap Up Slide- Importance of continuous self-reflection (Slide 15-16) | 5 minutes |
| **Total:** | **54 minutes** |

| **Part 2: Models of Cross-Cultural Communication** | |
| --- | --- |
| **Workshop Segment** | **Time** |
| Present opening case of that highlights cross-cultural communication: Lia Lee from “The Spirit Catches You and You Fall Down” by Anne Fadiman (Slide 18-20) | 5 minutes |
| Large group discussion questions regarding Lia Lee’s case (Slide 21-23) | 15 minutes |
| Speaker will present the LEARN Model and Kleinman’s 8 questions and move through slides explaining each step. A video will be played at the end to summarize the LEARN model (Slide 24-32) | 10 minutes |
| Group will be split into 5 breakout rooms or groups, and each assigned a letter of the LEARN model. They then will brainstorm how to use that letter in the Lia Lee Case. (Slide 34-36) | 10 minutes |
| Each small group will present their assigned letter to the large group and reflect on difficulties they may have had while performing the exercise. (Slide 37-38) | 10 minutes |
| Speaker will present summary slide (Slide 39) | 5 minutes |
| Participants will be given time to complete survey | 5 minutes |
| **Total:** | **60 min** |

**Intended Audience(s)**

This workshop is intended for residents who work with pediatric patients, including pediatric residents, family medicine residents, combined pediatric/medicine residents, preventative medicine residents, etcetera. This workshop works best for audiences who already have some experience working with pediatric patients and their families as it requires participants to draw from previous examples of cross-cultural encounters with patients and their families.

**Facilitator Qualifications and Responsibilities**

The facilitator has multiple roles in the delivery of this workshop. The first is to guide participants through the workshop via use of the provided slide set with use of the workshop outline below for timing. As part of this role, facilitators will explain various definitions to participants and will thus need to be familiar with these terms. Speaker notes are provided within the slide set as a guide for the facilitator. Facilitators will also need to break participants up into small groups of about 3-5 participants twice in the workshop- once for the self-reflection prompts, and again towards the end of the workshop when using the models of cross-cultural communication. The facilitator also has the important role of facilitating and encouraging both small group and large group discussion. Facilitators should be comfortable providing personal examples during the introduction of each self-reflection prompt as well as listening to each group’s discussion to facilitate large group reflection after each prompt. Facilitators should also be comfortable providing real examples of a case of poor cross-cultural communication and a case with which to demonstrate each letter of the LEARN model (one is provided for you in the slides, but a personal example tends to work best). Finally, the facilitator is responsible for keeping track of time during both the slide presentation and the small group discussions to leave adequate time for small group discussions and evaluation at the end of the workshop.

**Pre-requisites**

*Participants*

Participants will be sent Appendix A: Participant handout advance of the workshop. This includes required pre-reading. The pre-reading includes two parts. The first part of the participant handout goes through the definitions of culture, cultural humility, and illness explanatory models and provides the self-reflection prompts to be used during the workshop. The second part of the participant handout includes Kleinman's 8 questions, the LEARN model, and Negotiation tips that will be reviewed in the second half of the workshop. The participant handout can be emailed as attachments, or the email can include a QR code which will lead participants to a google drive housing this document.

*Facilitators*

Facilitators should familiarize themselves with the following definitions: culture, cultural humility, illness explanatory model. Facilitators should also familiarize themselves with Kleinman’s 8 questions and the LEARN model of cross-cultural communication. This can be done by reading the pre-reading that is also provided to participants. The facilitator should also be familiar with the case of Lia Lee from “The Spirit Catches You and You Fall Down.” A succinct review article written by the author, Anne Fadiman, is provided below. This is the case that will be used during the second half of the workshop to demonstrate a poor case of cross-cultural communication, and the case that will be re-imagined in small groups with the models presented in the workshop. Finally, facilitators should review the 3 self-reflection prompts and think of personal examples that they can provide to the group while introducing each prompt. Facilitators should also think of a case with which to demonstrate each letter of the LEARN model as they are presented.

*Recommended pre-reading for facilitators*

1. Fadiman A. "The spirit catches you and you fall down": epilepsy and the hmong. Epilepsy Behav. 2000 Feb;1(1):S3-8. doi: 10.1006/ebeh.2000.0037. PMID: 12609130.

*Optional additional pre-reading for facilitators*

1. Berlin EA, Fowkes WC Jr. A teaching framework for cross-cultural health care. Application in family practice. West J Med. 1983 Dec;139(6):934-8. PMID: 6666112; PMCID: PMC1011028.
2. Boutin-Foster C, Foster JC, Konopasek L. Viewpoint: physician, know thyself: the professional culture of medicine as a framework for teaching cultural competence. Acad Med. 2008 Jan;83(1):106-11. doi: 10.1097/ACM.0b013e31815c6753. PMID: 18162762.
3. Carrillo JE, Green AR, Betancourt JR. Cross-cultural primary care: a patient-based approach. Ann Intern Med. 1999 May 18;130(10):829-34. doi: 10.7326/0003-4819-130-10-199905180-00017. PMID: 10366373.
4. Kleinman A, Benson P. Anthropology in the clinic: the problem of cultural competency and how to fix it. PLoS Med. 2006 Oct;3(10):e294. doi: 10.1371/journal.pmed.0030294. PMID: 17076546; PMCID: PMC1621088.

**Required Resources**

*Common resources*

1. PowerPoint Slides (Appendix C) with speaker notes
   1. When applicable, slides in the PowerPoint presentation contain speaker notes that provide the facilitator with a summary of the objective of the slide and example commentary. Some slides are self-explanatory and therefore do not have associated speaker notes.
2. Participant Handout (Appendix A)
3. Ability to time small group sessions (either via wall clock, phone, etc.)

*In-Person Version*

1. Conference room with the following capabilities:
   1. Seating for 25-30 participants
   2. Ability to project a PowerPoint presentation and play a video with sound.
   3. Ability to move seats around to facilitate small group discussions.

*Virtual Version*

1. Professional zoom account with ability to break groups up into break out rooms and use whiteboard function during large group discussions
2. (Not required but recommended) Access to cloud share applications to use during small and large group discussions.

**Strategies for successful implementation**

*Common tips*

1. The most successful iterations of this workshop have been when the facilitator is engaged with both the content and the participants. Providing robust, clear examples for the self-reflection prompts make the prompts easy to understand and get the participants thinking about similar experiences in their lives. Having a prepared case to demonstrate each letter of the LEARN model was also helpful for participants to see how the model can be used in clinical situations. Sharing experiences as a facilitator also helps create a safe space for participants.
2. Having the participant handout readily accessible during the workshop was essential to participant participation in the second half of the workshop. In our workshops, this document was provided via QR code that participants could scan. Other options include emailing this document as an attachment to participants to open before and during the workshop or printing out the document to distribute during the workshop.
3. This workshop worked best when given as a part of intern orientation. At this point, self-reflection served 2 purposes: it allowed participants to reflect on their own cultural identity, but also allowed participants to get to know others in their intern class. Participants at this point in their training also had the space and time to do this sort of reflection and learning, work that often gets overshadowed by clinical duties later on in residency.
4. Ideally, this workshop should be given in a single 2-hour time frame. The workshop can technically be divided into two 1-hour sessions. If this is done, it is imperative that the sessions not occur too far apart (no farther than 1 week), and that the same participants that attended the first session attend the second session. It may also be useful to give participants a homework assignment, such as thinking of a case of poor cross-cultural communication to bring to the next session.
5. This workshop is best suited for about 25-30 participants, with small groups consisting of 3-5 participants per group.

*In-person session Tips*

1. During large group discussion following small group activities, short interactions with each small group can help encourage participation and knowledge sharing. In these interactions, facilitators can prompt discussion with more questions, and provide positive feedback on sharing thus far, as well as ask participants if they are comfortable sharing in the larger group setting.

*Virtual session Tips*

1. While participants are in break out rooms, it is helpful for the facilitator to move from room to room to facilitate discussion and share personal examples if discussion is slow. It is also helpful to listen to the discussion and encourage sharing with the large group if the participant feels comfortable.
2. Use of the whiteboard function and/or cloud share applications (such as word cloud programs) during small and large group activities as a visual representation of discussion may help keep participants engaged and encourage participation.

**Participant Handout (for reference)**

**Objectives**

By the end of this activity, learners will be able to:

1. Identify key elements of one’s own cultural identity and illness explanatory models through guided self-reflection.
2. Describe the impact of western medical culture on our own illness explanatory models as physicians.
3. Explain the impact of cultural beliefs and values of both the provider and patient/parent on patient physician communication.
4. Demonstrate use of cross-cultural communication models (i.e., LEARN model, Kleinman’s 8 questions) in small groups and with peers to build skills for culturally challenging communication situations.
5. Apply cross-cultural communication models in clinical settings to adequately explore a patient’s or parent’s perspective on illness, including their understanding and fears regarding work-up, diagnosis, and treatment, and to form a therapeutic alliance between the medical team and patient/family.

**Part 1- Definitions and Self-Reflection Prompts**

**Definitions**

What is culture?

- Culture is a difficult word to define. According to Boutin-Foster, et. Al, “Culture refers to integrated patterns of human behavior that include language, thoughts, communications, actions, beliefs, values, and institutions of racial, ethnic, religious, or social groups.”^1^
- Most importantly, culture is not static, it is fluid. Culture can change with big life events, including becoming a parent, the death of a loved one, or experiencing our own health problems. It is important to self-reflect on these experiences from time to time to become familiar with how they affect our own cultural identities and how we see the world.
- Additionally, medicine has its own culture which we are all a part of. We have been informed by our medical school curriculum as well as peers and mentors. This affects how we see different symptoms and disease entities.

What is cultural humility?

- Most of you are familiar with the term cultural competency. Cultural humility, however, is different. Instead of focusing on understanding certain cultures and other groups by becoming familiar with a set of beliefs, values, and traditions they have in common, it focuses on getting to know the individual themselves. This requires you to constantly engage in self-reflection about your own culture in order to better understand others.^2,3^
- Cultural humility demands understanding that culture is multifactorial, dynamic, fluid, and INDIVIDUAL.
- This is a lifelong process.

What is an illness explanatory model?

- Simply put, an illness explanatory model is how the patient explains his or her illness. More specifically, this model is how the patient understands what is causing their illness, why they feel the symptoms that they feel, and what they believe is necessary to treat their illness. Sometimes, this model matches ours as the medical team, and sometimes, our own model differs from our patients. Eliciting the patient’s illness explanatory model allows us to better understand their point of view and helps us approach the conversation regarding next steps with empathy and respect. ^4, 5^

**Exploring your own cultural identity**

Please reflect on the following prompts and questions. Be ready to discuss your thoughts in small groups. Feel free to use the spaces below under each prompt to write down your thoughts.

1. List 5 (or more!) components of your cultural identity.
2. How do these components, both separate and combined, affect how you see the world? How do they affect your illness explanatory model(s)?
3. Have you ever felt misunderstood due to your cultural beliefs/values? Have you ever been in or witnessed a patient encounter where there seemed to be a misunderstanding between the medical team and family due to different cultural beliefs/values?

*Reflection activity adapted from My Multicultural Self courtesy of the Southern Poverty Law Center

**Part 2- Models of Cross-Cultural Communication**

**Kleinman’s 8 Questions**^6^

What do you think has caused your problem?

Why do you think it started when it did?

What do you think your sickness does to you? How does it work?

How severe is your sickness? Will it have a short or long course?

What kind of treatment do you think you should receive?

What are the most important results you hope to receive from this treatment?

What are the chief problems your sickness has caused for you?

What do you fear most about your sickness?

**LEARN Model**^7^

L: ***Listen*** with sympathy and understanding to the patient’s perception of the problem

E: ***Explain*** your perceptions of the problem

A: ***Acknowledge*** and discuss the differences and similarities

R: ***Recommend*** treatment

N: ***Negotiate*** treatment

**Negotiation Tips**^8^

Describe diagnostic and treatment options in terms that are understandable to the patient and patient’s family

Determine the patient’s priorities

Present a reasonable management plan- try to prioritize management if possible

Determine patient and family’s level of acceptance of this plan by directly asking

Conflict may remain. If so, focus on higher priorities

**Part 3- Lia Lee Small Group Exercise**

Feel free to use the spaces below under each prompt to write down your thoughts.

**Group 1: L:** What questions would you ask to explore the parent’s understanding of Lia Lee’s condition and their understanding of the current treatment plan?

**Group 2: E:** How would you explain your (physician) perception of Lia’s illness and treatment?

**Group 3: A:** How would you acknowledge the parents’ illness explanatory model in your conversation and discuss similarities and differences of their model with yours in a non-judgmental way that shows empathy, curiosity, and respect?

**Group 4: R:** How would you phrase your recommendations to Lia’s parents?

**Group 5: N:** How would you approach negotiating the differences between your illness explanatory model and that of Lia’a parents? How might you incorporate Lia’s family’s concerns and approaches to the illness in your treatment plan? What concerns do you have about this?

**References**

1. Boutin-Foster C, Foster JC, Konopasek L. Viewpoint: physician, know thyself: the professional culture of medicine as a framework for teaching cultural competence. Acad Med. 2008 Jan;83(1):106-11. Doi: 10.1097/ACM.0b013e31815c6753. PMID: 18162762.
2. Chang ES, Simon M, Dong X. Integrating cultural humility into health care professional education and training. Adv Health Sci Educ Theory Pract. 2012 May;17(2):269-78. Doi: 10.1007/s10459-010-9264-1. Epub 2010 Dec 16. PMID: 21161680.
3. Tervalon M, Murray-García J. Cultural humility versus cultural competence: a critical distinction in defining physician training outcomes in multicultural education. J Health Care Poor Underserved. 1998 May;9(2):117-25. Doi: 10.1353/hpu.2010.0233. PMID: 10073197.
4. Dinos, S., Ascoli, M., Owiti, J., & Bhui, K. (2017). Assessing explanatory models and health beliefs: An essential but overlooked competency for clinicians. *BJPsych Advances,* *23*(2), 106-114. Doi:10.1192/apt.bp.114.013680
5. Kleinman A, Eisenberg L, Good B. Culture, illness, and care: clinical lessons from anthropologic and cross-cultural research. Ann Intern Med. 1978 Feb;88(2):251-8. Doi: 10.7326/0003-4819-88-2-251. PMID: 626456.
6. Kleinman A, Eisenberg L, Good B. Culture, illness, and care: clinical lessons from anthropologic and cross-cultural research. Ann Intern Med. 1978 Feb;88(2):251-8. Doi: 10.7326/0003-4819-88-2-251. PMID: 626456.
7. Berlin EA, Fowkes WC Jr. A teaching framework for cross-cultural health care. Application in family practice. West J Med. 1983 Dec;139(6):934-8. PMID: 6666112; PMCID: PMC1011028.
8. Carrillo JE, Green AR, Betancourt JR. Cross-cultural primary care: a patient-based approach. Ann Intern Med. 1999 May 18;130(10):829-34. Doi: 10.7326/0003-4819-130-10-199905180-00017. PMID: 10366373.

Facilitator guide developed by Angie Buttigieg, MD
