# Supplementary material for: How to Teach Cross-Cultural Communication: A Workshop Using the Experiential Learning Model
Source: MedEdPORTAL. 2023 Nov 21;19:11365. doi: 10.15766/mep_2374-8265.11365 (PMC10662213; doi:10.15766/mep_2374-8265.11365)
Supplement: Supplementary file 1 — Participant Handout.docxFacilitator Guide.docxSlide Presentation.pptxRetrospective Pre-Post Survey.docx3-Month Postworkshop Survey.docx [file mep_2374-8265.11365-s001.zip › _Educational Summary Report_11365.pdf]

MedEdPORTAL® The AAMC Journal of Teaching and Learning Resources

Original Publication Open Access

How to Teach Cross-Cultural Communication: A Workshop Using the Experiential Learning Model

Angie Buttigieg, MD*, Deanna Chieco, MD, EdM, Maria Maldonado, MD, Kelly Wang, MPH, Allison Gault, MD, Leora Mogilner, MD

*Corresponding author: angie.buttigieg@mountsinai.org

Abstract

Introduction: The United States population is diversifying, leading to higher rates of cultural, ethnic, and racial discordance between medical teams and patients. Studies show that pediatric residents lack training in cross-cultural communication (CCC). **Methods:** We based learning objectives on the AAMC's Tool for Assessing Cultural Competency Training. The workshop design was based on Kolb's experiential learning model. In 2020-2021, we delivered this 2-hour workshop to trainees at two large, urban sites. We administered two surveys to evaluate our workshop: a retrospective pre-post survey following the workshop and a 3-month follow-up survey. Using 5-point Likert scales, participants rated their awareness of the effect of their own cultural identity on CCC and familiarity with and confidence using CCC models. We analyzed responses using Wilcoxon signed rank tests. **Results:** Sixty-two trainees participated in the workshop; 44 completed the retrospective pre-post survey (71%). After the workshop, 36% were extremely aware of the effect of their own cultural identity on CCC compared to 4% before the workshop ($p < .001$). Confidence managing cross-cultural misunderstandings when conveying a diagnosis and explaining disease management increased after the workshop (70% vs. 25%, $p < .001$; 70% vs. 20%, $p < .001$, respectively). Twelve participants completed a 3-month follow-up survey (27%). **Discussion:** A workshop using the experiential learning model to teach CCC increased participants' awareness of how their cultural identity impacted CCC and familiarity with and confidence in using two CCC models. This workshop offers pediatric program directors a tool to enhance their CCC curricula and meet ACGME requirements.

Keywords
Cross-Cultural Communication, Self-Reflection, Cased-Based Learning, Communication Skills, Cultural Competence, Diversity, Equity, Inclusion

Educational Objectives

By the end of this activity, learners will be able to:

1. Identify key elements of their own cultural identity and illness explanatory models through guided self-reflection.
2. Recognize the impact of Western medical culture on their own illness explanatory models as physicians.
3. Explain the impact of cultural beliefs and values of both the provider and patient/parent on patient-physician communication.
4. Demonstrate use of cross-cultural communication models (i.e., LEARN model, Kleinman's eight questions) in small groups and with peers to build skills for culturally challenging communication situations.
5. Apply cross-cultural communication models in clinical settings to adequately explore a patient's or parent's perspective on illness, including their understanding and fears regarding workup, diagnosis, and treatment, and to form a therapeutic alliance between medical team and patient/family.

Citation:
Buttigieg A, Chieco D, Maldonado M, Wang K, Gault A, Mogilner L. How to teach cross-cultural communication: a workshop using the experiential learning model. *MedEdPORTAL*. 2023;19:11365.
https://doi.org/10.15766/mep_2374-8265.11365

Introduction

The United States' patient population is diversifying at a faster rate than medical teams, leading to higher rates of cultural, ethnic, and racial discordance between patients and their medical teams.[1,2] Lack of knowledge about how culture affects health care and lack of cross-cultural communication (CCC) skills are hypothesized to contribute to continued health care disparities between racial and ethnic groups.[1]

Copyright © 2023 Buttigieg et al. This is an open-access publication distributed under the terms of the Creative Commons Attribution license.

In 2022, the Accreditation Council for Graduate Medical Education (ACGME) released updated common program requirements for pediatric residency. The ability to respectfully interact with diverse patient populations is now included in the professionalism core competency.[3] However, a 2018 study conducted at a large pediatric program revealed that pediatric residents continued to cite a lack of cultural humility training in their programs, including lack of training in CCC.[4] In addition, a 2021 study done at a large urban pediatric residency program found that pediatric residents without CCC training reported feeling significantly less skilled at eliciting patient and parent perspectives regarding illness than those who had received training.[5] Residents wanted more training on how to identify personal implicit bias and change behaviors based on this self-assessment.[5] An editorial published in 2020 detailed gaps in current communication curricula within medical school training. It stated that educators assume medical students have previous experience with diverse communication encounters and do not recognize the need to explicitly teach the skills required to communicate sensitively and effectively with patients from diverse backgrounds.[6] With the goal of closing this curricular gap, we developed a workshop using a revised version of the Tool for Assessing Cultural Competence Training (TACCT)[7,8] as a guide. A survey instrument developed by the American Association of Medical Colleges, the TACCT can be used by program leadership and trainees to assess the adequacy of a program's cultural competency training as well as to find and address any gaps. Our workshop focused on teaching pediatric residents the effect of their cultural identity on the patient-physician relationship and providing them with tools for successful CCC with their patients.

MedEdPORTAL has published several resources on cultural self-awareness and CCC. One resource encourages discovery of one's own cultural identity through various self-reflection prompts and a game.[9] Others use standardized cases to teach various CCC models, including ETHNIC (explanation, treatment, healers, negotiate, intervention, collaboration), Kleinman's eight questions, and the LEARN (listen, explain, acknowledge, recommend, negotiate) model.[10-13] Other curricula focus on older patients, hematology/oncology patients, and psychiatry patients.[14-16] Finally, a further resource teaches CCC using a slide presentation with videos highlighting both good and bad cross-cultural interactions.[11] However, while geared towards medical students and introducing Kleinman's eight questions, that resource does not teach the LEARN model, nor does it incorporate self-reflection as a tool for discovering one's own culture and conducting effective CCC. There is no resource rooted in general pediatrics that combines both self-reflection and teaching of CCC models with relevant pediatric cases to teach CCC skills to pediatric residents.

Methods

Workshop Development and Content

We developed this workshop using Kern's six-step method for curriculum development.[17] The objectives were based on TACCT domains IV and VI and guided by Bloom's taxonomy.[18] Domain IV focused on CCC and recommended knowledge, skills, and attitudes that should be incorporated within a curriculum. Domain VI concentrated on self-reflection and the culture of medicine itself. We used these two domains to inform the content of our workshop and both the retrospective pre-post survey and the 3-month follow-up survey. The overarching goals of the workshop were to learn how to use self-reflection techniques to better understand one's own culture and the culture of medicine, identify any inherent bias, and apply this new perspective along with CCC tools to improve comfort with CCC. We also emphasized the lifelong learning and work required by effective CCC and encouraged participants to continue to self-reflect and refine their skills as their lives changed. We based the structure of this workshop on the experiential learning model (concrete experience, reflective observation, abstract conceptualization, active experimentation).[19] Participants brought concrete experiences of past cross-cultural encounters to the workshop. They took part in small-group self-reflection on their own cultural identities and these past experiences. We then presented Kleinman's eight questions[20] and the LEARN model.[21] Kleinman's eight questions provided a framework for eliciting a patient's illness explanatory model, and the LEARN model helped facilitate empathetic CCC. Finally, participants learned about a challenging cultural communication case and engaged in active experimentation by applying these models to this case in small groups. The Institutional Review Board office (Program for the Protection of Human Subjects) at Mount Sinai Hospital determined this study to be exempt.

Implementation

The primary target audience for this resource was pediatric, medicine/pediatric, and family medicine residents of all postgraduate years, though it could also be applicable to medical students. Prior to the workshop, we sent participants one handout (Appendix A), which included three parts. Part 1 contained definitions of culture, cultural humility, and illness explanatory models, as well as the self-reflection prompts that would be used in the workshop. Part 2 defined Kleinman's eight questions and the LEARN model and reviewed negotiation skills for a cross-

Copyright © 2023 Buttigieg et al. This is an open-access publication distributed under the terms of the Creative Commons Attribution license.

cultural encounter. Part 3 contained discussion questions that would be used when discussing the patient case. During the workshop, these handouts were also made available via QR code.

The facilitator guide (Appendix B) offered a detailed approach to presenting the workshop. The key role of the facilitator was to guide participants through the workshop and facilitate and encourage small- and large-group discussions. While no specific background training was needed to lead the workshop, expertise and comfort communicating with patients of different backgrounds were preferred. Facilitators needed to familiarize themselves with the definitions and CCC models presented prior to the workshop. This could be done by reading the participant handout included as a reference in the facilitator guide (Appendix B). Facilitators also had to familiarize themselves with the patient case (review article provided in Appendix A) and come prepared with a case with which to demonstrate the LEARN model. Finally, it was recommended that the facilitator review the self-reflection prompts prior to the workshop to come up with personal examples that would engage discussion. The facilitator used a slide presentation to guide the workshop (Appendix C). After a brief introduction, there was an overview during which relevant definitions were reviewed. Then, the facilitator presented the self-reflection prompts and split the participants into small groups to discuss each one (Appendix A). Following each prompt, participants shared what they had discussed in their small groups. The second half of the workshop opened with a CCC case featuring Lia Lee from *The Spirit Catches You and You Fall Down*.[22] The facilitator posed several discussion questions about the case, and the case was discussed as a large group. Then, the facilitator presented both the LEARN model and Kleinman's eight questions (Appendix A). Ideally, the facilitator would utilize a case from their own experience to show the use of the LEARN model and Kleinman's eight questions in real time. Next was shown a video that demonstrated the use of these models. Finally, the facilitator reintroduced the Lia Lee case, split participants into small groups, and assigned each group a letter of the LEARN model. Each group's task was to reimagine their part of the conversation. The small groups then came together to share their part of the reimagined conversation with the whole group (Appendix A). At the end of the workshop, participants were given 5 minutes to complete the retrospective pre-post survey electronically. The survey was provided via QR code on the last slide of the presentation.

In total, we gave the workshop to 44 participants over five sessions. One session was in person, three sessions were hybrid sessions, and one session used a virtual platform. All virtual sessions were conducted using the Zoom platform.

The original intent was to have all workshops be held in person. However, due to the COVID-19 pandemic, the workshop was offered in several ways. It was first implemented exclusively in person during intern orientation. The workshop was held in a small conference room. Space was flexible and allowed chairs to be rearranged to facilitate small-group discussion. Subsequently, the workshop was given in a hybrid in-person and virtual format, once as two 1-hour noon conferences and once as one 2-hour conference. The final session was conducted virtually. We found that our workshop easily lent itself to virtual learning with the use of breakout rooms to facilitate small-group discussion. We were able to reach more learners as well as easily accommodate scheduling conflicts with the use of virtual sessions. The facilitator guide provided tips on how best to present this workshop both live and virtually.

Evaluation

We drafted two surveys to evaluate our workshop. A retrospective pre-post survey[23] was drafted using the New World Kirkpatrick model[24] as a guide (Appendix D). We also drafted a 3-month follow-up survey to assess retained knowledge and attitudes, as well as real-time use of skills gained during the workshop (Appendix E). Content experts in medical education and CCC performed multiple reviews of both surveys to ensure content validity. We administered a preliminary retrospective pre-post survey to the outgoing third-year residents who had participated in the workshop as a pilot prior to formal data gathering and edited the survey based on their feedback. We chose to pilot the workshop and the retrospective pre-post survey with the outgoing third-year residents at their senior retreat to ensure turnout and participation. This retreat was scheduled close to the time of graduation, and therefore, the residents would not be participating in the formal curriculum and would not skew survey results. The retrospective pre-post survey used a mixed methods approach with both free-text and Likert-style questions. We chose to administer a single retrospective pre-post survey immediately following the workshop to reduce response shift bias and improve response rate.[23] In addition to evaluating the workshop, participants were asked to recall their familiarity with and awareness of CCC concepts prior to the workshop and compare them to their familiarity and awareness after the workshop. Participants were similarly asked to rate their confidence in using CCC models before versus after the workshop. We presented a QR code that led to the retrospective pre-post survey on the last slide of the presentation. We also sent participants two weekly email reminders with links to the survey.

Copyright © 2023 Buttigieg et al. This is an open-access publication distributed under the terms of the Creative Commons Attribution license.

Three months after the workshop, the 3-month follow-up survey was distributed to participants via email (Appendix E). Survey data were collected and managed using REDCap version 13.1.37, hosted at the Icahn School of Medicine at Mount Sinai.[25,26] We linked the retrospective pre-post survey to the 3-month follow-up survey using a unique identifier selected by each resident. Participants could opt to enter a raffle for a gift card as an incentive for participation in the 3-month follow-up survey.

We defined Likert scale scores for each question as follows: 1 = *Not at all,* 2 = *Slightly,* 3 = *Moderately,* 4 = *Quite,* 5 = *Extremely.* Participant responses from the retrospective pre-post survey were referred to as preworkshop and postworkshop, with the intervention being the workshop itself, and responses from the 3-month follow-up survey referred to as 3 months after the workshop. The Wilcoxon signed rank test was used to determine if a participant's familiarity, awareness, sense of importance, and confidence related to CCC changed from preworkshop to postworkshop. Differences in Likert scores from preworkshop and postworkshop were calculated (preworkshop minus postworkshop). We conducted similar analyses to compare differences in preworkshop and postworkshop responses among different postgraduate years. The Mann-Whitney *U* test was used to assess whether differences in survey responses differed between individuals from different hospitals, between location of medical school training, and between postgraduate years. When performing this analysis, postgraduate level was dichotomized into two categories: (1) PGY 1 and other and (2) PGY 2-PGY 5. A result was considered statistically significant at the $p < .05$ level of significance. All analyses were performed using SAS version 9.4 (SAS Institute).

Results

Sixty-two trainees participated in the workshop. Forty-four completed the retrospective pre-post survey following the workshop (71%). Table 1 displays descriptive statistics concerning participant demographics and history of CCC training in the past. Most residents were female (70%), White (59%), not Hispanic or Latino (86%), at least third-generation Americans (41%), in the pediatric residency training program (66%), and PGY 1 (66%). Most participants reported that they had not previously participated in a curriculum focused on CCC (57%).

Table 2 provides descriptive statistics on participants' perceptions of the workshop. Overall, participants were either quite satisfied or extremely satisfied with the structure and the facilitator of the workshop (98%). After the workshop, the majority of the residents reported feeling quite confident with using Kleinman's eight questions (50%) and the LEARN model of CCC (55%). Responses of quite or extremely familiar with Kleinman's eight questions and the LEARN model increased postworkshop compared to preworkshop (68% vs. 7%, $p < .001$, and 84% vs. 2%, $p < .001$, respectively). Responses of quite or extremely aware to questions regarding the impact of one's own cultural identity on one's approach to medicine, impact of cultural identity on CCC, and the impact of Western medical culture on one's own illness explanatory model also significantly increased ($p < .05$ for all). Finally, confidence in communicating with patients and families with different cultural beliefs, eliciting different health perspectives of patients and families, and managing cross-cultural misunderstandings regarding workup, conveying a diagnosis, and explaining disease management significantly increased postworkshop ($p < .05$ for all; Table 3).

We found no significant difference in responses based on PGY status. Interestingly, we saw a significant difference by site of medical school training, with those individuals who studied outside the United States feeling more confident in managing cross-cultural misunderstandings when explaining disease management and more confident in eliciting different health perspectives of patients and their families before the workshop compared to those who studied in the United States (*p*s = .03 and .04, respectively).

Of the 44 participants who participated in the initial workshop, 20 participated in the 3-month follow-up survey. Among the 20 responses, 12 identifiers were linked to the retrospective preworkshop and postworkshop responses. For almost all questions, participants on average reported feeling less familiar, less aware, and less confident and having lost a sense of importance 3 months after the workshop compared to right after the workshop (median differences in Likert score ≤ 0). However, the median differences in Likert scores did not change for the questions "How important did you feel understanding a patient's cultural identity was to effective CCC?", "How confident did you feel communicating with patients/families with different cultural beliefs?", "How confident did you feel managing cross-cultural misunderstandings regarding the workup for a diagnosis in the inpatient setting?", and "How confident did you feel managing cross-cultural misunderstandings when explaining disease management in the inpatient setting?" (median differences = 0). Thus, we can conclude that some attitudes and skills were sustained in our sample 3 months after the workshop (Table 4).

Finally, our 3-month survey assessed the diversity of patient population encountered by participants, their real-time use of the models presented, and their anticipated use in the future. The majority of participants said that they were exposed to a

Copyright © 2023 Buttigieg et al. This is an open-access publication distributed under the terms of the Creative Commons Attribution license.

Table 1. Participant Demographics and History of Past Cross-Cultural Communication Training ($N = 44$)

Variable	No. (%)
Gender	
Female	31 (70)
Male	13 (30)
Ethnicity	
Hispanic or Latino	5 (11)
Not Hispanic or Latino	38 (86)
Prefer not to answer	1 (2)
Race	
Asian	7 (16)
Black/African American	7 (16)
White	26 (59)
Other—please specify	2 (4)
Prefer not to answer	2 (4)
Generational status	
1st generation	15 (34)
2nd generation	9 (20)
3rd or higher generation	18 (41)
I don't know	2 (4)
Postgraduate year	
1	29 (66)
2	4 (9)
3	4 (9)
4	2 (4)
5	2 (4)
Other (MS3, medical student[a])	3 (7)
Program	
Categorical pediatric resident	29 (66)
Internal medicine-pediatrics resident	3 (7)
Triple board pediatrics, adult psychiatry, child and adolescent psychiatry resident, or combined pediatrics	5 (11)
Other (MS3, medical student,[a] preliminary resident, preventive medicine/internal medicine)	7 (16)
Hospital	
Kravis	31 (70)
Elmhurst	13 (30)
Have you ever participated in a curriculum or workshop focused on cross-cultural communication?	
Yes	11 (25)
No	25 (57)
I don't know	8 (18)

[a]Some medical students wrote MS3, and some wrote medical student. The medical student response may mean either a third- or fourth-year medical student.

patient of a different culture than their own in over half of their encounters (83%) but used the Kleinman model to facilitate CCC in only 0%-50% of relevant patient encounters. Participants indicated that they used the LEARN model to facilitate CCC in 0%-75% of their encounters. Although they did not use it as often, 75% of participants indicated they were likely to use these models in the future while on the inpatient floor. They also indicated that the Kleinman model and LEARN model were moderately or quite valuable in guiding CCC (50% and 58%, respectively).

Discussion

This workshop was created to address the need for a robust CCC curriculum in pediatrics. From June to November 2021, the workshop was given five times to different groups of residents and medical students. Overall, participants were satisfied with the workshop and believed that the stated objectives were met. After the workshop, most respondents reported being quite or extremely familiar with both Kleinman's eight questions and the LEARN model. They also reported understanding the impact of cultural identity on CCC. Finally, most participants reported confidence in communicating with patients and families with different cultural beliefs, eliciting different health perspectives of patients and families, and managing cross-cultural misunderstandings regarding workup, conveying a diagnosis, and explaining disease management.

While several gains in skills, attitudes, and knowledge were lost after 3 months in the group that completed the 3-month survey, what did persist was respondents' understanding of the importance of a patient's cultural identity to effective CCC and their confidence in communicating effectively with patients of different backgrounds. Although the specifics of the different models taught may not have been retained, we believe that

Copyright © 2023 Buttigieg et al. This is an open-access publication distributed under the terms of the Creative Commons Attribution license.

Table 2. Perceptions of the Workshop and Confidence in Using CCC Models After the Workshop ($N = 44$)

Variable	No. (%)
Structure of the workshop[a]	
Moderately satisfied	1 (2)
Quite satisfied	12 (28)
Extremely satisfied	30 (70)
Facilitator of the workshop[a]	
Quite satisfied	9 (21)
Extremely satisfied	34 (79)
Finding didactic session reviewing definitions to be useful	
Moderately useful	2 (4)
Quite useful	17 (39)
Extremely useful	25 (57)
Finding small-group reflection to be useful	
Moderately useful	1 (2)
Quite useful	12 (27)
Extremely useful	31 (70)
Finding didactic session reviewing Kleinman's eight questions and LEARN model useful	
Moderately useful	5 (11)
Quite useful	9 (20)
Extremely useful	30 (68)
Finding video demonstrating use of Kleinman's eight questions and LEARN model to be useful[b]	
Slightly useful	1 (2)
Moderately useful	6 (14)
Quite useful	12 (29)
Extremely useful	23 (55)
Finding small-group exercise reimagining Lia Lee's case through CCC models to be useful	
Moderately useful	4 (9)
Quite useful	13 (30)
Extremely useful	27 (61)
Overall, how successful was this workshop in addressing its stated objectives?	
Moderately successful	3 (7)
Quite successful	11 (25)
Extremely successful	30 (68)
After attending this workshop, how confident do you feel using Kleinman's eight questions to better understand a family's illness explanatory model?	
Slightly confident	1 (2)
Moderately confident	13 (30)
Quite confident	22 (50)
Extremely confident	8 (18)
After attending this workshop, how confident do you feel using the LEARN model to come to an agreement with a patient/parent regarding a diagnostic and/or treatment plan?	
Slightly confident	1 (2)
Moderately confident	11 (25)
Quite confident	24 (55)
Extremely confident	8 (18)

Abbreviations: CCC, cross-cultural communication; LEARN, listen, explain, acknowledge, recommend, negotiate.
[a]Missing data on one individual (2%).
[b]Missing data on two individuals (4%).

participants have internalized the precepts of the workshop and are implementing them, even if they are not using the models taught specifically. Faculty development of those who supervise residents may help ensure that skills learned in the workshop are implemented in real-time practice. We believe that, moving forward, the best way to encourage use of these CCC models and ultimately change behavior is through faculty modeling in real time with patients on rounds or during one-on-one clinical encounters in the days and weeks following the workshop. Furthermore, there was no resident representation when creating this workshop. We would recommend including residents in the development of further workshops to address this issue more specifically.

Interestingly, our survey also revealed that, even before the workshop, participants who studied medicine outside the United States felt more confidence in their CCC skills than participants who studied in the United States. This finding was evident during small- and large-group discussions. Participants shared examples of having to reconcile cultural differences in the United States with cultural norms in the countries where they trained, including differences in the perceived role of women in

Copyright © 2023 Buttigieg et al. This is an open-access publication distributed under the terms of the Creative Commons Attribution license.

Table 3. Familiarity, Awareness, and Confidence in CCC Pre- Versus Postworkshop

Question	*Mdn* (IQR)[a]		*Mdn* Difference (IQR)[a]	p[b]
	Preworkshop	Postworkshop		
How familiar were you with Kleinman's eight questions?	1 (1, 2)	4 (3, 4)	−2 (−3, −2)	<.001
How familiar were you with the LEARN model of CCC?[c]	1 (1, 2)	4 (4, 5)	−3 (−3, −2)	<.001
How aware were you of the impact of your own cultural identity on your approach to medicine?	3 (3, 4)	4 (4, 5)	−1 (−1, 0)	<.001
How aware were you of the impact of your own cultural identity on CCC?	3 (3, 4)	4 (4, 5)	−1 (−1, 0)	<.001
How important did you feel understanding a patient's cultural identity was to effective CCC?	4 (4, 5)	5 (4, 5)	0 (−1, 0)	<.001
How aware were you of the impact of Western medical culture on your own illness explanatory model?	4 (3, 4)	4.5 (4 ,5)	−1 (−1, 0)	<.001
How confident did you feel communicating with patients/families with different cultural beliefs?	3 (3, 4)	4 (3, 4)	−1 (−1, 0)	<.001
How confident did you feel eliciting different health perspectives of patients and their families?	3 (3, 4)	4 (3, 4)	−1 (−1, 0)	<.001
How confident did you feel managing cross-cultural misunderstandings regarding the workup for a diagnosis in the inpatient setting?[d]	3 (3, 3.5)	4 (3.5, 4)	−1 (−1, 0)	.004
How confident did you feel managing cross-cultural misunderstandings when conveying a diagnosis in the inpatient setting?	3 (3, 3.5)	4 (3, 4)	−1 (−1, 0)	<.001
How confident did you feel managing cross-cultural misunderstandings when explaining disease management in the inpatient setting?	3 (3, 3)	4 (3, 4)	−1 (−1, 0)	<.001

Abbreviations: CCC, cross-cultural communication; IQR, interquartile range; LEARN, listen, explain, acknowledge, recommend, negotiate.
[a]Likert scores for familiar, aware, important, and confident were defined as follows: 1 = *Not at all,* 2 = *Slightly,* 3 = *Moderately,* 4 = *Quite,* 5 = *Extremely.*
[b]The Wilcoxon signed rank test was used to calculate pre-post survey response differences in Likert scores.
[c]Missing one response in postsurvey.
[d]Missing 24 responses.

decision-making, patient autonomy, practices of disclosing illness, and normalcy of women having more than one reproductive partner. The increased confidence in CCC skills seen in foreign medical graduates thus makes sense considering these residents navigate cross-cultural interactions daily, in both their personal and professional lives. This hypothesis is further supported by an editorial by Baugh, Vanderbilt, and Baugh suggesting that lived experience with different cultures improves CCC and that U.S.-trained medical students lack this experience, leading to poor CCC skills and the development of biases.[6]

We learned several lessons in the implementation of this workshop. The workshop works best for a group of 10-30 participants, with small groups of three to five for self-reflection prompts and active experimentation. The room setup should be conducive to reorganizing into small groups, ideally with mobile chairs. Most of the workshops were given in one 2-hour block; however, for scheduling purposes, two workshops were given as consecutive 1-hour noon conferences. While the workshop has a natural halfway point, we found that momentum and participation significantly decreased when split into two noon conferences. If the workshop must be given in two sessions, it is imperative to ensure the same group of learners is present at both sessions, and it may be valuable to give homework after the first session to keep momentum going. Because of the COVID-19 pandemic, this workshop was offered both in person, virtually, and as a hybrid model. Anecdotally, we found that the groups that were all in person had better engagement than the hybrid sessions as evidenced by ease of small- and large-group participation and robustness of discussion. We believe increased engagement results in a more effective workshop. However, we recognize the importance of virtual workshops because education is trending in this direction and the format has the ability to reach more participants. To improve engagement with this workshop when given virtually, we suggest use of the breakout room function for small-group discussion. We also suggest the use of collaborative cloud applications and whiteboard functions to encourage interaction. Use of personal examples by the facilitator during the explanation of small-group prompts was essential to encouraging participation of the group. One workshop was given during intern orientation, which seemed to be a good time for the workshop for several reasons. First, the self-reflection prompts served as an icebreaker for the interns, who did not know each other well. Second, interns during orientation do not have clinical duties to distract them from the workshop. Having the QR code with access to Kleinman's eight questions and the LEARN model was vital to the second half of the workshop so participants could refer to it during their active experimentation activity.

There were several limitations in the implementation and evaluation of this workshop. Due to the COVID-19 pandemic, several of the workshops were given in hybrid fashion, with some participants in person and some virtually. This made breaking up into small groups logistically difficult. Additionally, splitting the

Copyright © 2023 Buttigieg et al. This is an open-access publication distributed under the terms of the Creative Commons Attribution license.

Table 4. 3-Month Follow-up Survey

Question	*Mdn* (IQR)[a]		*Mdn* Difference (IQR)[a]	*p*[b]
	Postworkshop	3 Months After Workshop		
How familiar were you with Kleinman's eight questions?	4 (4, 4.5)	2 (2, 3)	−1.5 (−2.5, −1)	<.001
How familiar were you with the LEARN model of CCC?	4 (4, 5)	2 (1.5, 3)	−2 (−3, −1)	<.001
How aware were you of the impact of your own cultural identity on your approach to medicine?	4 (4, 5)	3 (3, 4)	−1 (−2, 0)	.008
How aware were you of the impact of your own cultural identity on CCC?	4 (4, 5)	4 (3, 4)	−1 (−1, 0)	.02
How important did you feel understanding a patient's cultural identity was to effective CCC?	5 (4.5, 5)	5 (4, 5)	0 (−0.5, 0)	>.99
How aware were you of the impact of Western medical culture on your own illness explanatory model?	5 (4, 5)	4 (3, 4)	−1 (−1, −0.5)	.02
How confident did you feel communicating with patients/families with different cultural beliefs?	4 (3.5, 4)	4 (3, 4)	0 (−1, 0)	.22
How confident did you feel eliciting different health perspectives of patients and their families?	4 (4, 4)	4 (3, 4)	−0.5 (−1, 0)	.03
How confident did you feel managing cross-cultural misunderstandings regarding the workup for a diagnosis in the inpatient setting?[c]	4 (4, 4)	3 (3, 4)	0 (−1, 0)	.50
How confident did you feel managing cross-cultural misunderstandings when conveying a diagnosis in the inpatient setting?	4 (3.5, 4)	3 (3, 4)	−1 (−1, 0)	.02
How confident did you feel managing cross-cultural misunderstandings when explaining disease management in the inpatient setting?	4 (3, 4)	3.5 (3, 4)	0 (−1, 0)	.36
How confident did you feel using Kleinman's eight questions to better understand a family's illness explanatory model?	4 (4, 4.5)	2 (1.5, 3)	−2 (−2, −1.5)	.001
How confident did you feel using the LEARN model to come to an agreement with a patient/parent regarding diagnostic and/or treatment plan?	4 (4, 4)	2.5 (1.5, 3)	−1.5 (−2, −1)	.001

Abbreviations: CCC, cross-cultural communication; IQR, interquartile range; LEARN, listen, explain, acknowledge, recommend, negotiate.
[a]Likert scores for familiar, aware, important, and confident were defined as follows: 1 = *Not at all,* 2 = *Slightly,* 3 = *Moderately,* 4 = *Quite,* 5 = *Extremely.*
[b]The Wilcoxon signed rank test was used to calculate pre-post survey response differences in Likert scores.
[c]Seven individuals were missing postworkshop responses.

workshop into two sessions meant that some residents from the first session did not attend the second session and vice versa, making the data more difficult to interpret. We also could not link some of the pre-post survey responses because some participants forgot their unique IDs. This resulted in a smaller sample at the 3-month mark. Finally, we had a small response rate to the postworkshop 3-month survey, making it hard to draw any conclusions from the data.

This workshop provides residency program directors with a tool that can help satisfy the ACGME requirement for communication with patients with diverse backgrounds. Further work needs to be done on how to sustain knowledge, skill, and attitude gains after the workshop. Future studies could also address patient satisfaction with their providers before and after this workshop. While our curriculum is geared towards pediatric residents, family medicine, medicine/pediatric, and preventative health residents as well as medical students on their pediatric rotations also participated in and enjoyed the workshop. We believe that the concepts taught in this workshop apply to a wide range of learners, including medical students at any stage of their education and residents of all specialties. By changing the cases to represent the desired patient population, the workshop can be further customized. Additionally, we believe that faculty would also benefit from this workshop and that the workshop would lend itself well to faculty development or retreat settings.

Appendices

A. Participant Handout.docx

B. Facilitator Guide.docx

C. Slide Presentation.pptx

D. Retrospective Pre-Post Survey.docx

E. 3-Month Postworkshop Survey.docx

All appendices are peer reviewed as integral parts of the Original Publication.

Angie Buttigieg, MD: Assistant Professor, Department of Pediatrics, Icahn School of Medicine at Mount Sinai; ORCID: https://orcid.org/0000-0001-7361-8504

Deanna Chieco, MD, EdM: Assistant Professor, Department of Pediatrics, Icahn School of Medicine at Mount Sinai

Maria Maldonado, MD: Adjunct Associate Professor of Medicine, Department of Medicine, Icahn School of Medicine at Mount Sinai

Kelly Wang, MPH: Biostatistician, Department of Population Health Science and Policy, Icahn School of Medicine at Mount Sinai

Copyright © 2023 Buttigieg et al. This is an open-access publication distributed under the terms of the Creative Commons Attribution license.

Allison Gault, MD: Associate Professor, Department of Pediatrics, Icahn School of Medicine at Mount Sinai

Leora Mogilner, MD: Associate Professor, Department of Pediatrics, Icahn School of Medicine at Mount Sinai

Disclosures
None to report.

Funding/Support
None to report.

Ethical Approval
The Mount Sinai Hospital Program for the Protection of Human Subjects deemed further review of this project not necessary.

References

1. Shen MJ, Peterson EB, Costas-Muñiz R, et al. The effects of race and racial concordance on patient-physician communication: a systematic review of the literature. *J Racial Ethn Health Disparities*. 2018;5(1):117-140. https://doi.org/10.1007/s40615-017-0350-4
2. Diversity in medicine: facts and figures 2019. Association of American Medical Colleges. 2019. Accessed October 10, 2023. http://www.aamc.org/data-reports/workforce/report/diversity-medicine-facts-and-figures-2019
3. *ACGME Common Program Requirements (Residency)*. Accreditation Council for Graduate Medical Education; 2021. Updated July 1, 2022. Accessed October 10, 2023. https://www.acgme.org/globalassets/pfassets/programrequirements/cprresidency_2022v3.pdf
4. Rule ARL, Reynolds K, Sucharew H, Volck B. Perceived cultural competency skills and deficiencies among pediatric residents and faculty at a large teaching hospital. *Hosp Pediatr*. 2018;8(9):554-569. https://doi.org/10.1542/hpeds.2017-0110
5. Barber Doucet H, Ward VL, Johnson TJ, Lee LK. Implicit bias and caring for diverse populations: pediatric trainee attitudes and gaps in training. *Clin Pediatr (Phila)*. 2021;60(9-10):408-417. https://doi.org/10.1177/00099228211035225
6. Baugh AD, Vanderbilt AA, Baugh RF. Communication training is inadequate: the role of deception, non-verbal communication, and cultural proficiency. *Med Educ Online*. 2020;25(1):1820228. https://doi.org/10.1080/10872981.2020.1820228
7. *Tool for Assessing Cultural Competence Training (TACCT)*. Association of American Medical Colleges. Accessed October 10, 2023. https://www.aamc.org/media/20841/download?attachment
8. Lie D, Boker J, Crandall S, et al. A revised curriculum Tool for Assessing Cultural Competency Training (TACCT) in health professions education. *MedEdPORTAL*. 2009;5:3185. https://doi.org/10.15766/mep_2374-8265.3185
9. Elliott D. Cultural self-awareness workshop. *MedEdPORTAL*. 2009;5:1128. https://doi.org/10.15766/mep_2374-8265.1128
10. Van Schaik E, Roat C, Fronek L. Culture & End-of-Life Care: an e-learning course. *MedEdPORTAL*. 2014;10:9958. https://doi.org/10.15766/mep_2374-8265.9958
11. Goleman J. Doctor-patient communication: speed bumps and hiccups. *MedEdPORTAL*. 2010;6:7908. https://doi.org/10.15766/mep_2374-8265.7908
12. Stuart E, Bereknyei S, Long M, Blankenburg R, Weak V, Garcia R. Standardized patient cases for skill building in patient-centered, cross-cultural interviewing (Stanford Gap Cases). *MedEdPORTAL*. 2012;8:9133. https://doi.org/10.15766/mep_2374-8265.9133
13. Blue A. Cultural competency interviewing case using the ETHNIC mnemonic. *MedEdPORTAL*. 2009;5:155. https://doi.org/10.15766/mep_2374-8265.155
14. Agness-Whittaker CF, Macedo L. Aging, culture, and health communication: exploring personal cultural health beliefs and strategies to facilitate cross-cultural communication with older adults. *MedEdPORTAL*. 2016;12:10374. https://doi.org/10.15766/mep_2374-8265.10374
15. Rao AN, Warad D, Rodriguez V. Cross-cultural care training for pediatric hematology/oncology fellows. *MedEdPORTAL*. 2017;13:10543. https://doi.org/10.15766/mep_2374-8265.10543
16. Elliott D, St. George C, Signorelli D, Trial J. Stereotypes and bias at the psychiatric bedside—cultural competence in the third-year required clerkships. *MedEdPORTAL*. 2010;6:1150. https://doi.org/10.15766/mep_2374-8265.1150
17. Thomas PA, Kern DE, Hughes MT, Chen BY, eds. *Curriculum Development for Medical Education: A Six-Step Approach*. 3rd ed. Johns Hopkins University Press; 2016.
18. Bloom BS, ed. *Taxonomy of Educational Objectives: The Classification of Educational Goals—Handbook 1. Cognitive Domain*. Longmans, Green; 1956.
19. Kolb DA. *Experiential Learning: Experience as the Source of Learning and Development*. 2nd ed. Pearson Education; 2015.
20. Kleinman A, Benson P. Anthropology in the clinic: the problem of cultural competency and how to fix it. *PLoS Med*. 2006;3(10):e294. https://doi.org/10.1371/journal.pmed.0030294
21. Berlin EA, Fowkes WC Jr. A teaching framework for cross-cultural health care—application in family practice. *West J Med*. 1983;139(6):934-938.
22. Fadiman A. *The Spirit Catches You and You Fall Down: A Hmong Child, Her American Doctors, and the Collision of Two Cultures*. Farrar, Straus and Giroux; 1997.
23. Geldhof GJ, Warner DA, Finders JK, Thogmartin AA, Clark A, Longway KA. Revisiting the utility of retrospective pre-post designs: the need for mixed-method pilot data. *Eval Program Plann*. 2018;70:83-89. https://doi.org/10.1016/j.evalprogplan.2018.05.002

Copyright © 2023 Buttigieg et al. This is an open-access publication distributed under the terms of the Creative Commons Attribution license.

24. Kirkpatrick DL, Kirkpatrick JD. *Evaluating Training Programs: The Four Levels*. 3rd ed. Berrett-Koehler; 2006.

25. Harris PA, Taylor R, Thielke R, Payne J, Gonzalez N, Conde JG. Research electronic data capture (REDCap)—a metadata-driven methodology and workflow process for providing translational research informatics support. *J Biomed Inform*. 2009;42(2): 377-381. https://doi.org/10.1016/j.jbi.2008.08.010

26. Harris PA, Taylor R, Minor BL, et al.; REDCap Consortium. The REDCap Consortium: building an international community of software platform partners. *J Biomed Inform*. 2019;95:103208. https://doi.org/10.1016/j.jbi.2019.103208

Received: March 29, 2023
Accepted: September 14, 2023
Published: November 21, 2023

Copyright © 2023 Buttigieg et al. This is an open-access publication distributed under the terms of the Creative Commons Attribution license.
