# Supplementary material for: How to Teach Cross-Cultural Communication: A Workshop Using the Experiential Learning Model
Source: MedEdPORTAL. 2023 Nov 21;19:11365. doi: 10.15766/mep_2374-8265.11365 (PMC10662213; doi:10.15766/mep_2374-8265.11365)
Supplement: Supplementary file 1 — Participant Handout.docxFacilitator Guide.docxSlide Presentation.pptxRetrospective Pre-Post Survey.docx3-Month Postworkshop Survey.docx [file mep_2374-8265.11365-s001.zip › C. Slide Presentation.pptx]

## Slide 1
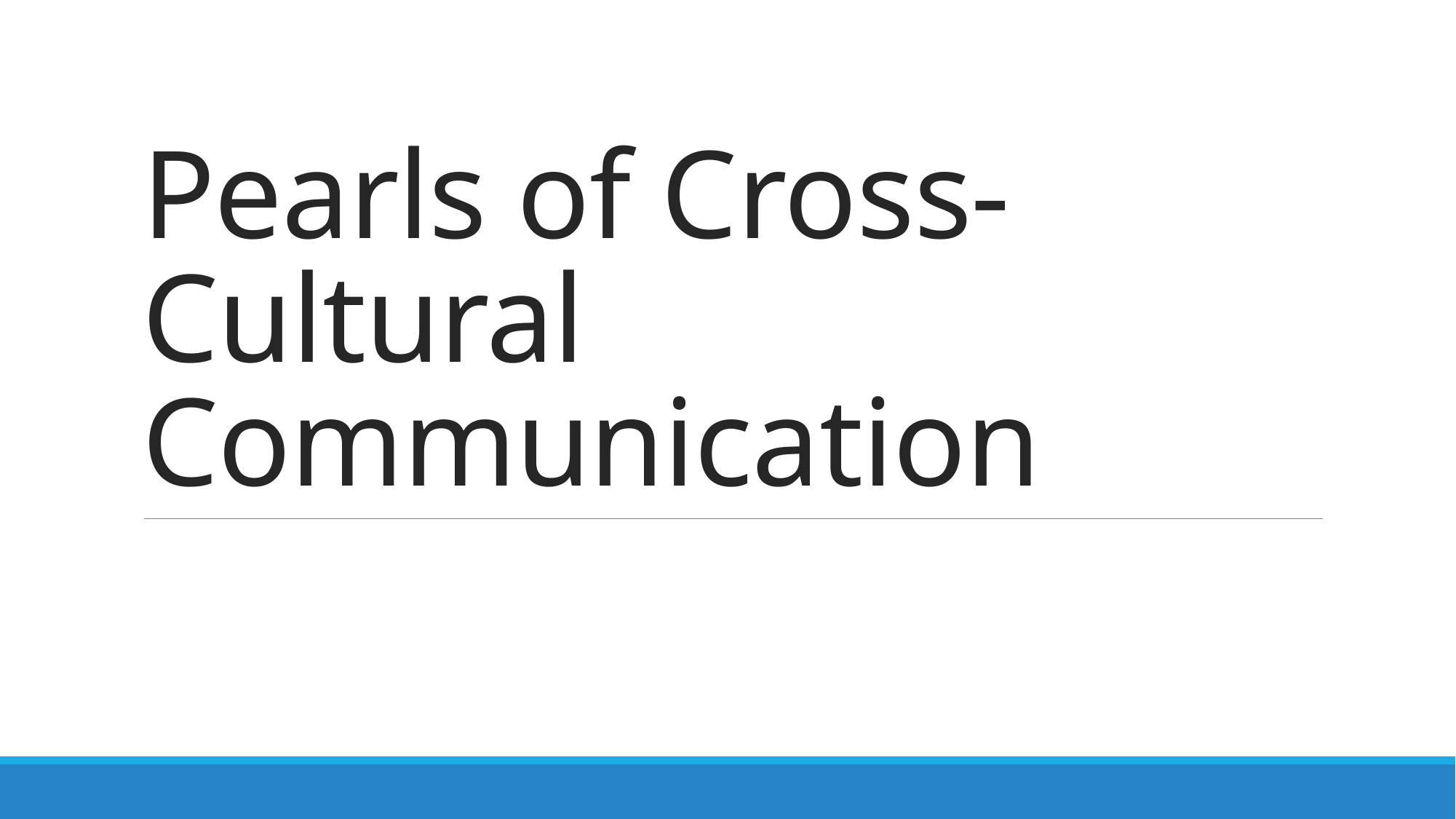

# Pearls of Cross-Cultural Communication

## Slide 2
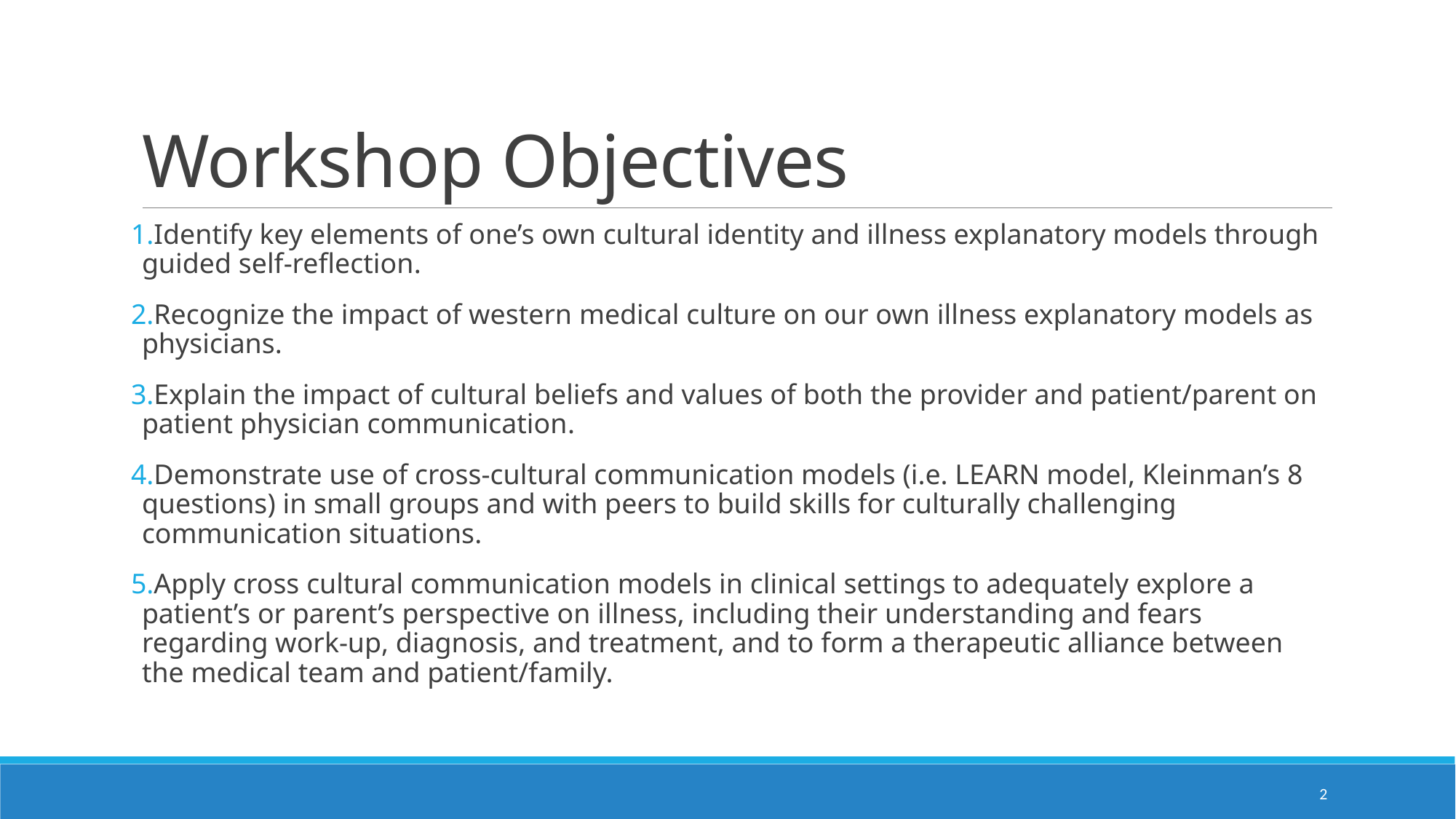

# Workshop Objectives
Identify key elements of one’s own cultural identity and illness explanatory models through guided self-reflection​.
Recognize the impact of western medical culture on our own illness explanatory models as physicians.
Explain the impact of cultural beliefs and values of both the provider and patient/parent on patient physician communication​.
Demonstrate use of cross-cultural communication models (i.e. LEARN model, Kleinman’s 8 questions) in small groups and with peers to build skills for culturally challenging communication situations.
Apply cross cultural communication models in clinical settings to adequately explore a patient’s or parent’s perspective on illness, including their understanding and fears regarding work-up, diagnosis, and treatment, and to form a therapeutic alliance between the medical team and patient/family.
2

## Slide 3
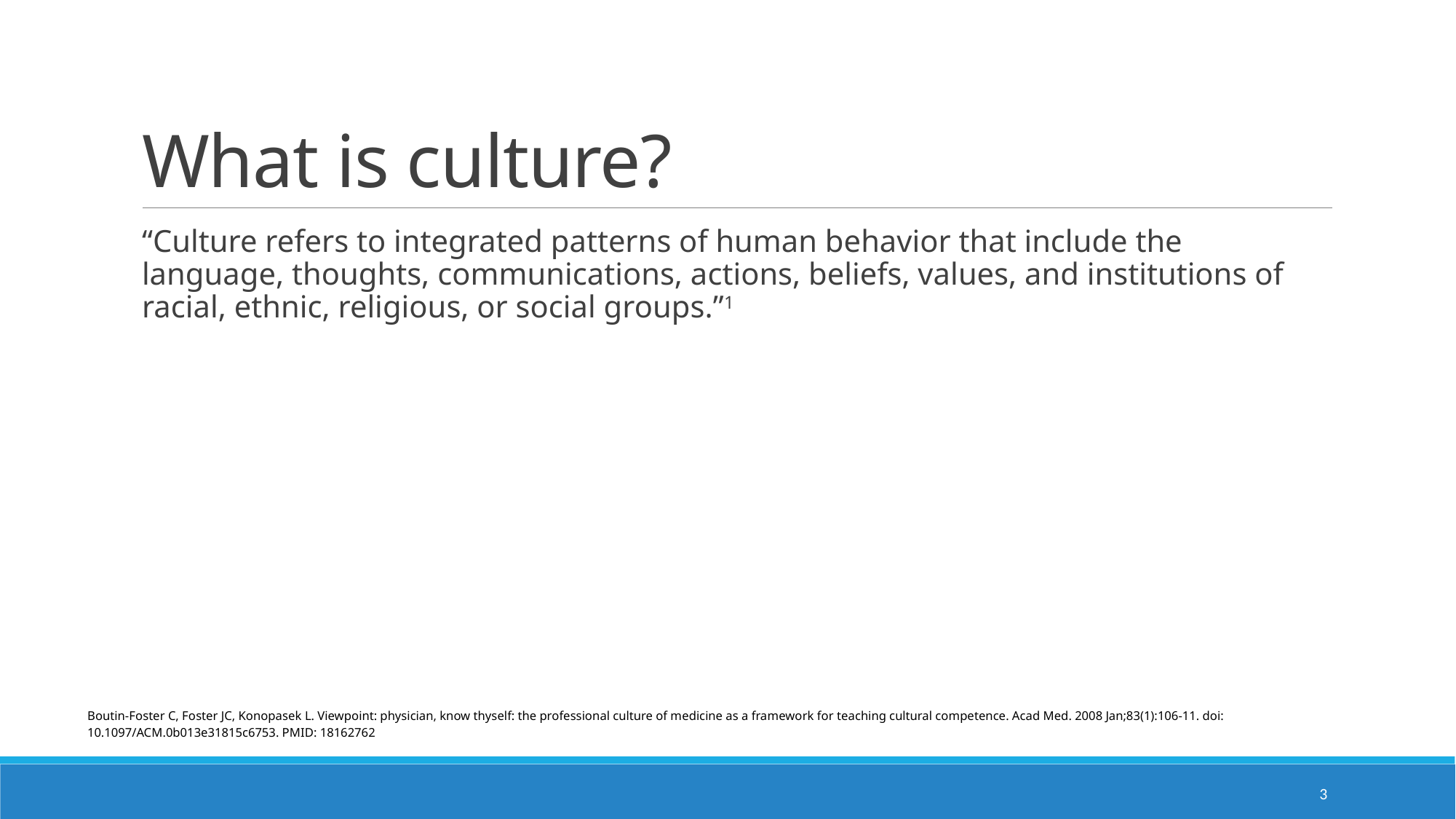

# What is culture?
“Culture refers to integrated patterns of human behavior that include the language, thoughts, communications, actions, beliefs, values, and institutions of racial, ethnic, religious, or social groups.”1
Boutin-Foster C, Foster JC, Konopasek L. Viewpoint: physician, know thyself: the professional culture of medicine as a framework for teaching cultural competence. Acad Med. 2008 Jan;83(1):106-11. doi: 10.1097/ACM.0b013e31815c6753. PMID: 18162762.
3

## Slide 4
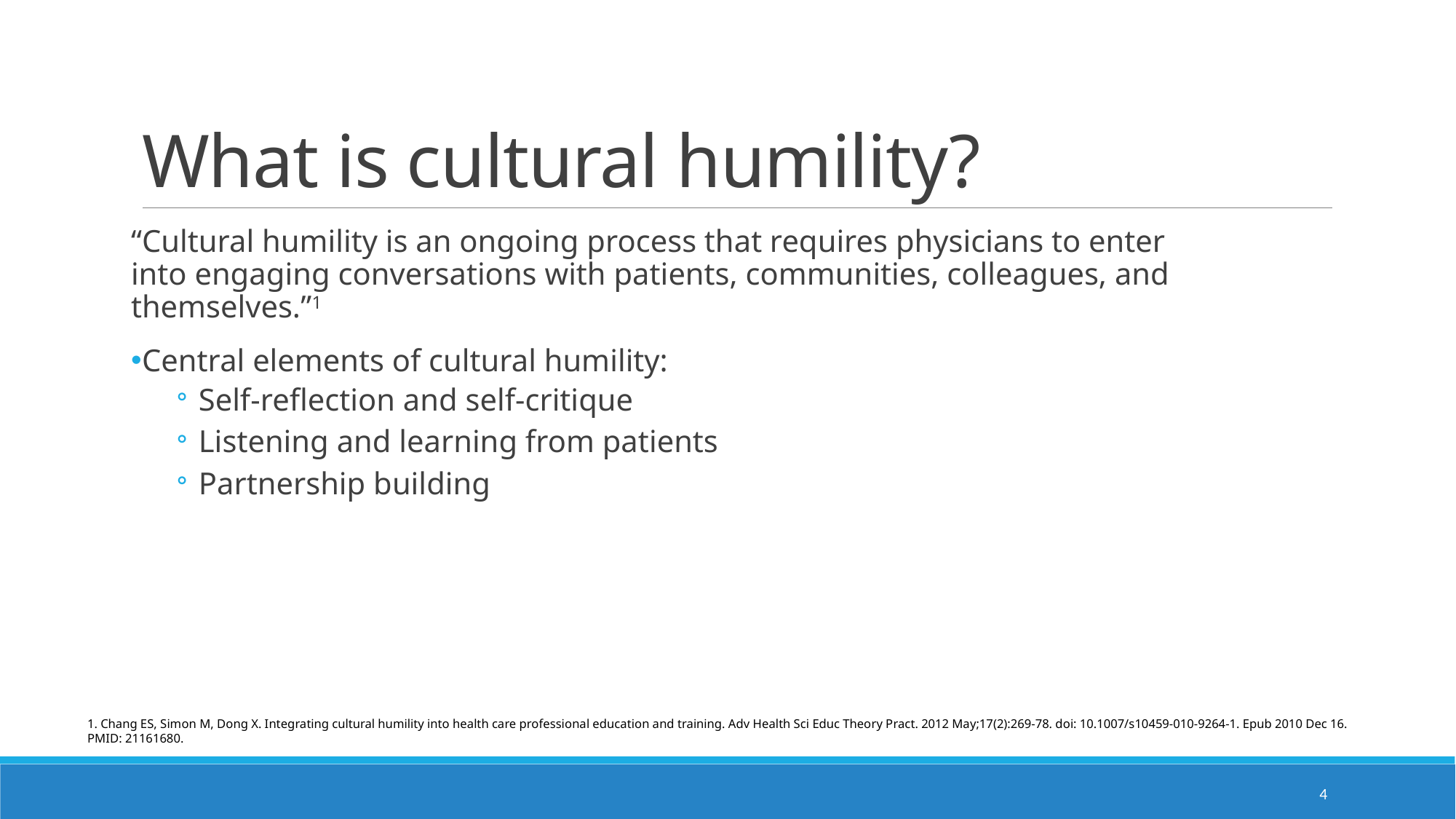

# What is cultural humility?
“Cultural humility is an ongoing process that requires physicians to enter into engaging conversations with patients, communities, colleagues, and themselves.”1​
Central elements of cultural humility:​
Self-reflection and self-critique​
Listening and learning from patients​
Partnership building​
1. Chang ES, Simon M, Dong X. Integrating cultural humility into health care professional education and training. Adv Health Sci Educ Theory Pract. 2012 May;17(2):269-78. doi: 10.1007/s10459-010-9264-1. Epub 2010 Dec 16. PMID: 21161680.
4

## Slide 5
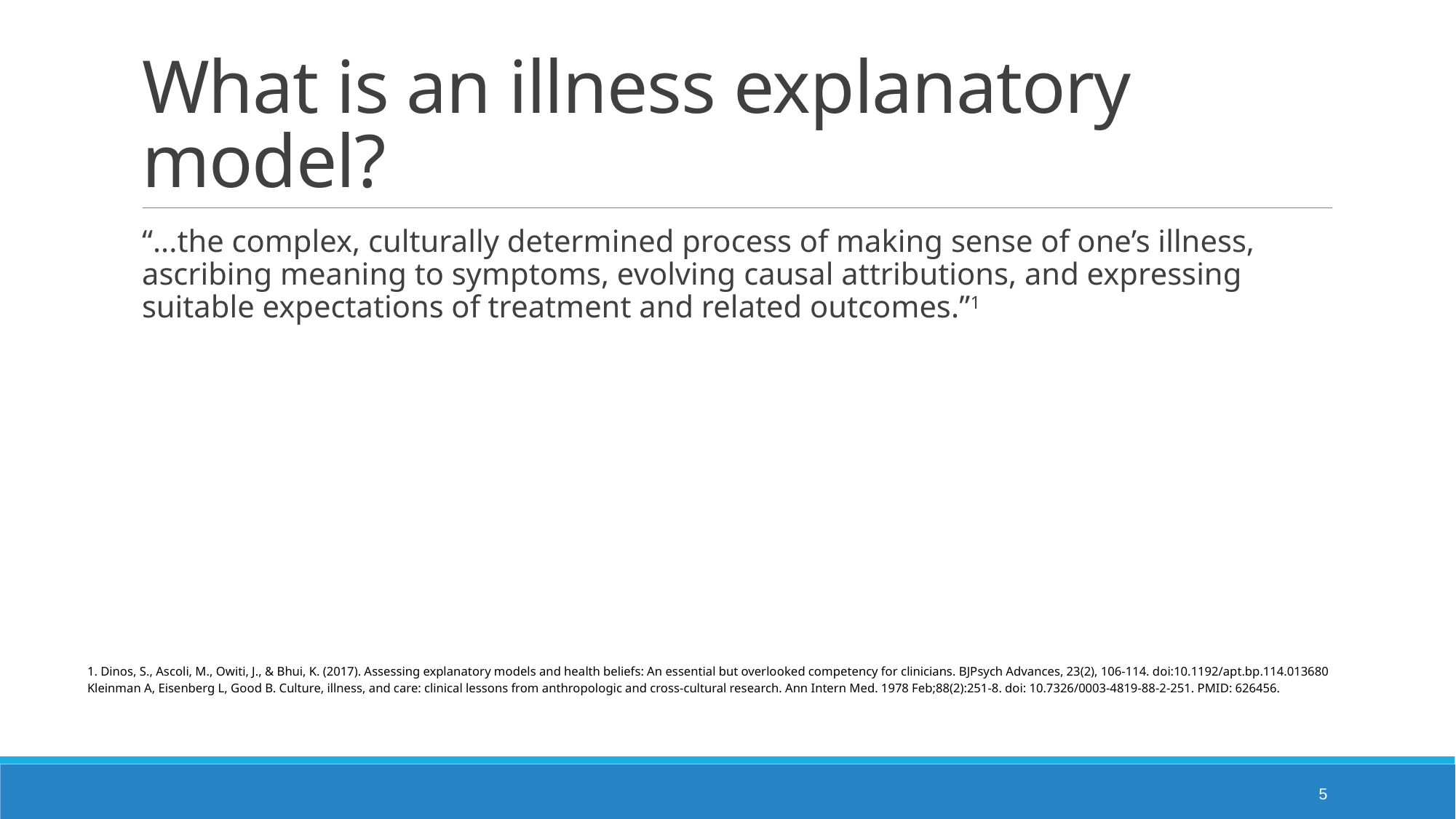

# What is an illness explanatory model?
“...the complex, culturally determined process of making sense of one’s illness, ascribing meaning to symptoms, evolving causal attributions, and expressing suitable expectations of treatment and related outcomes.”1
1. Dinos, S., Ascoli, M., Owiti, J., & Bhui, K. (2017). Assessing explanatory models and health beliefs: An essential but overlooked competency for clinicians. BJPsych Advances, 23(2), 106-114. doi:10.1192/apt.bp.114.013680​
Kleinman A, Eisenberg L, Good B. Culture, illness, and care: clinical lessons from anthropologic and cross-cultural research. Ann Intern Med. 1978 Feb;88(2):251-8. doi: 10.7326/0003-4819-88-2-251. PMID: 626456.​
​
5

## Slide 6
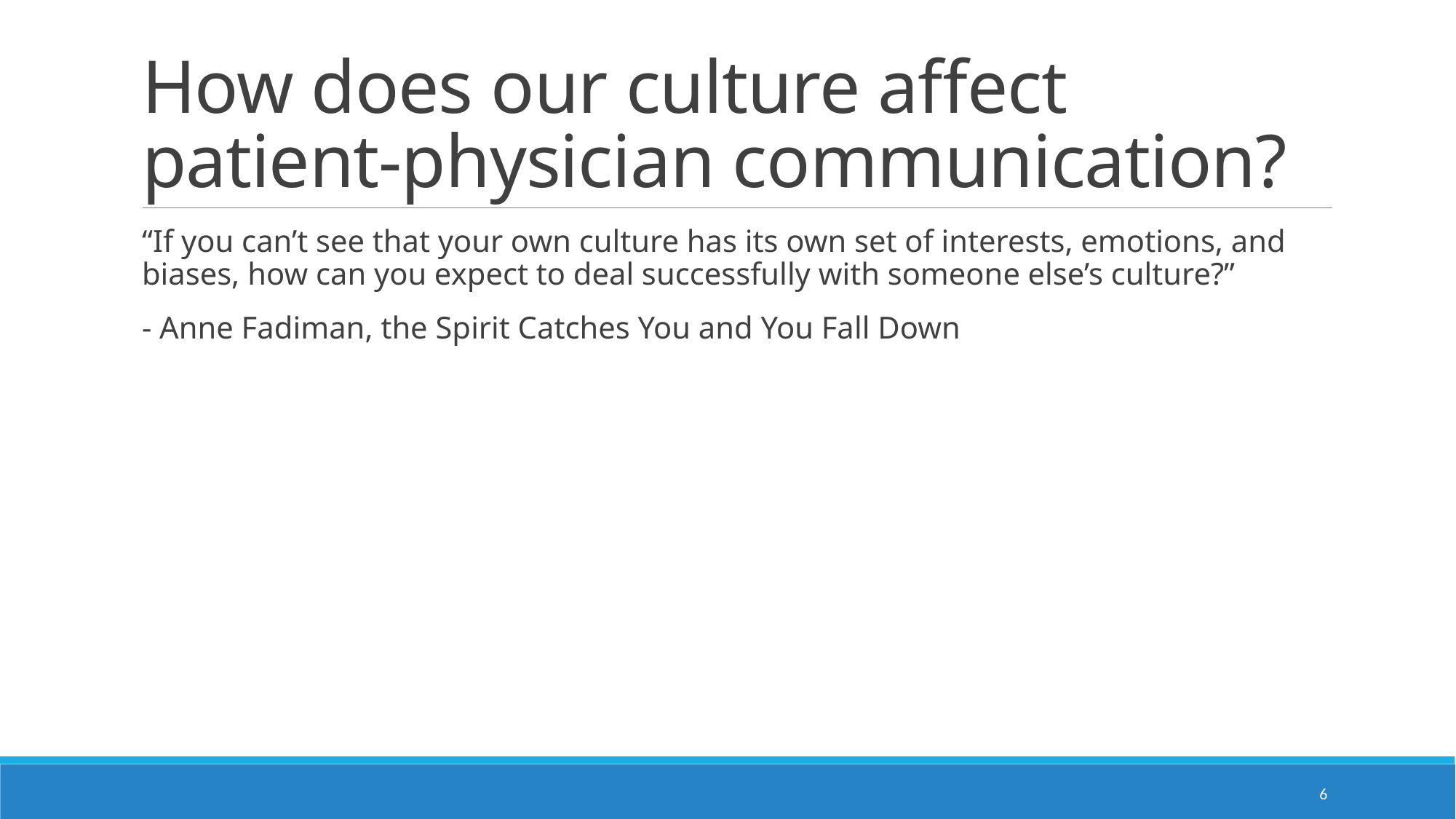

# How does our culture affect patient-physician communication?
“If you can’t see that your own culture has its own set of interests, emotions, and biases, how can you expect to deal successfully with someone else’s culture?”​
- Anne Fadiman, the Spirit Catches You and You Fall Down
6

## Slide 7
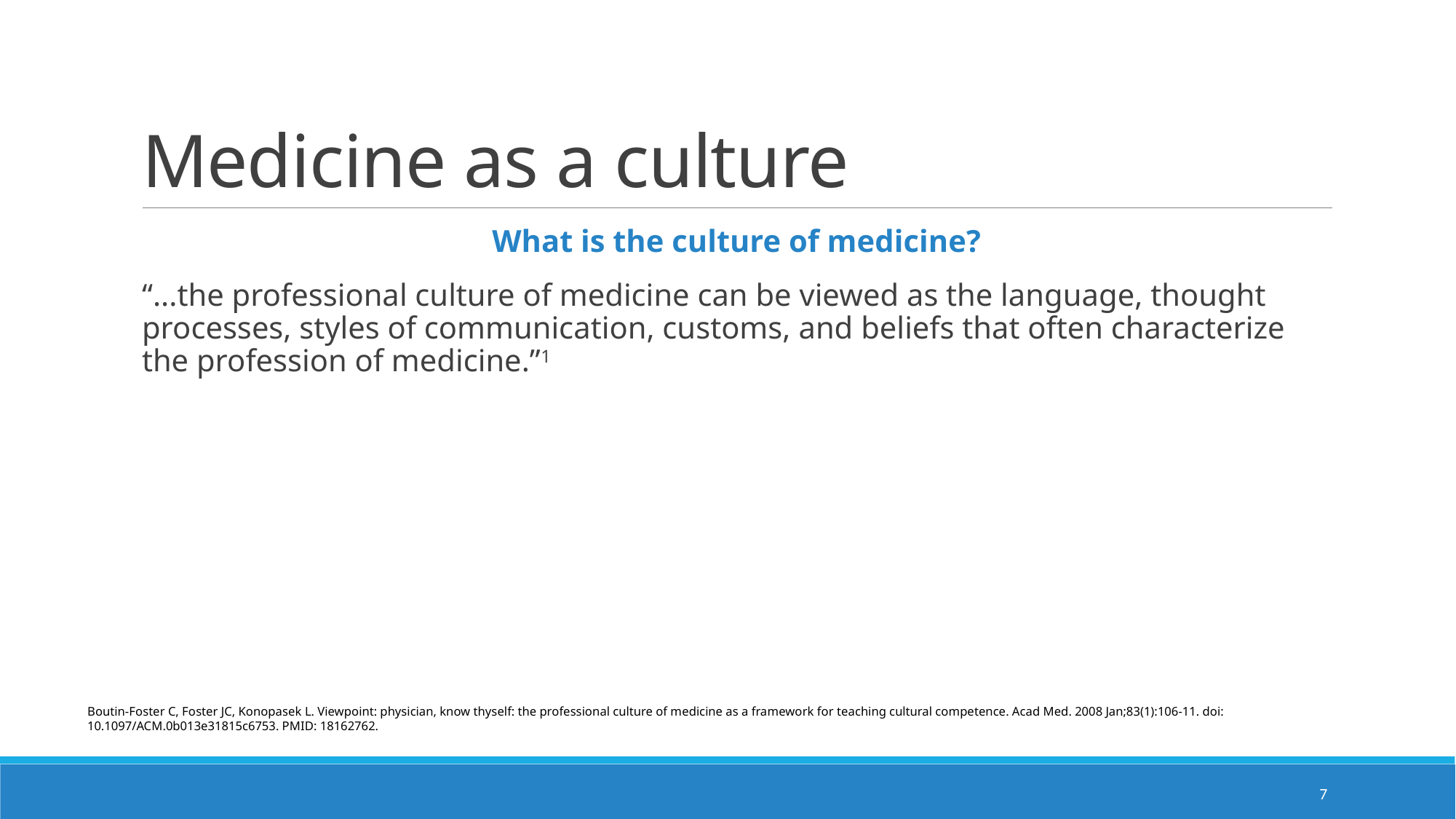

# Medicine as a culture
What is the culture of medicine?
“...the professional culture of medicine can be viewed as the language, thought processes, styles of communication, customs, and beliefs that often characterize the profession of medicine.”1
Boutin-Foster C, Foster JC, Konopasek L. Viewpoint: physician, know thyself: the professional culture of medicine as a framework for teaching cultural competence. Acad Med. 2008 Jan;83(1):106-11. doi: 10.1097/ACM.0b013e31815c6753. PMID: 18162762.
7

## Slide 8
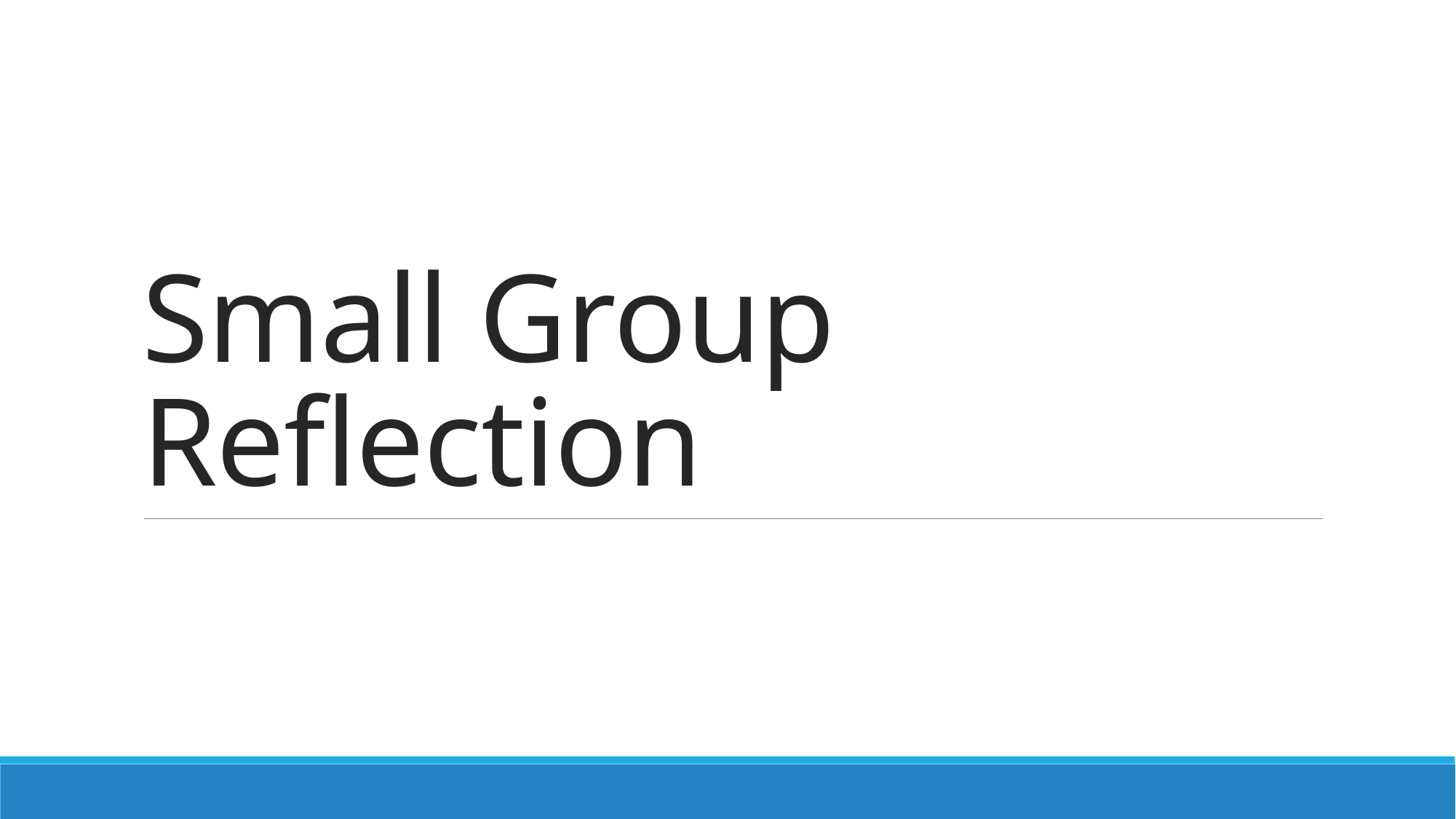

# Small Group Reflection

## Slide 9
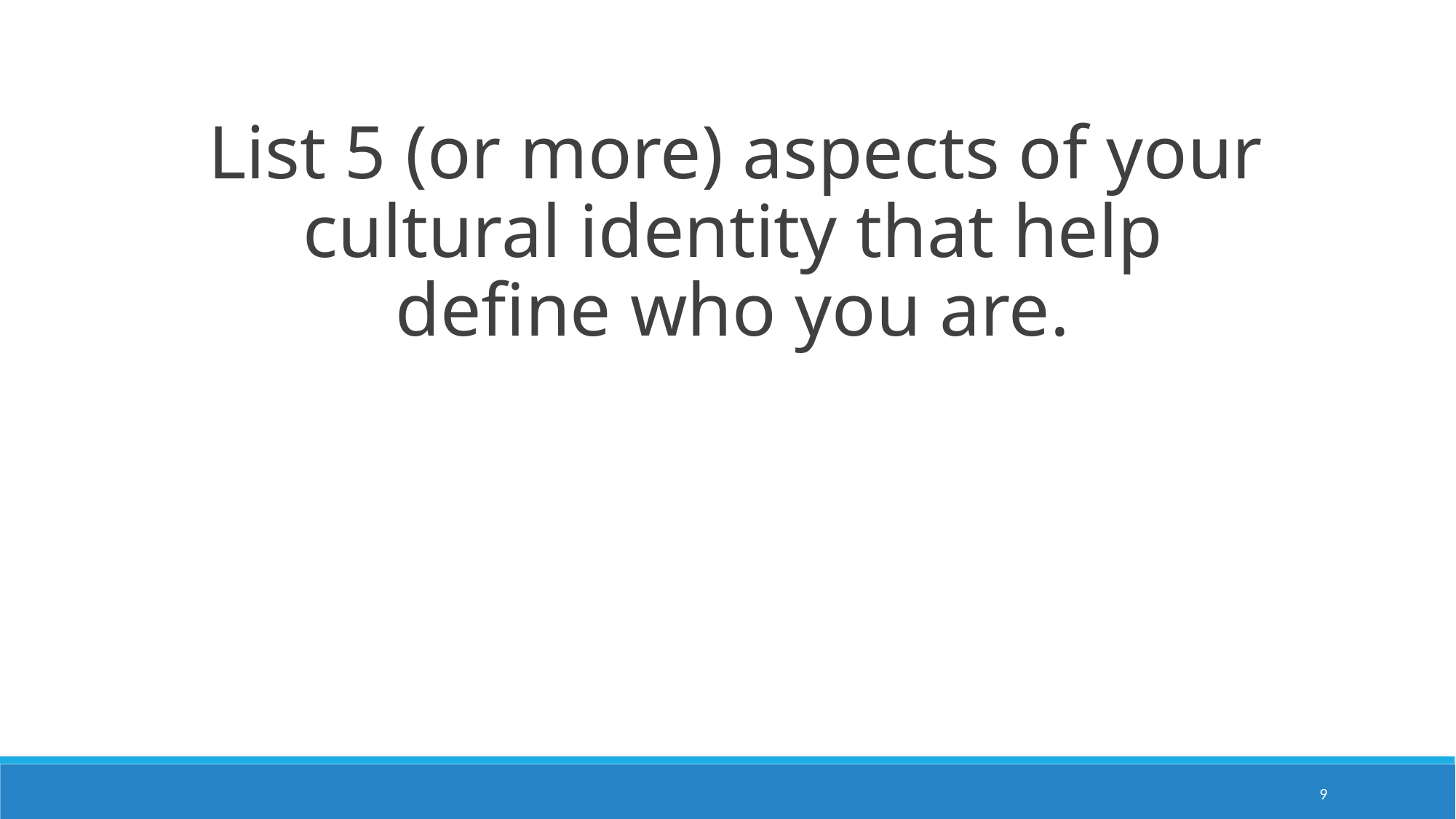

List 5 (or more) aspects of your cultural identity that help define who you are.​
9

## Slide 10
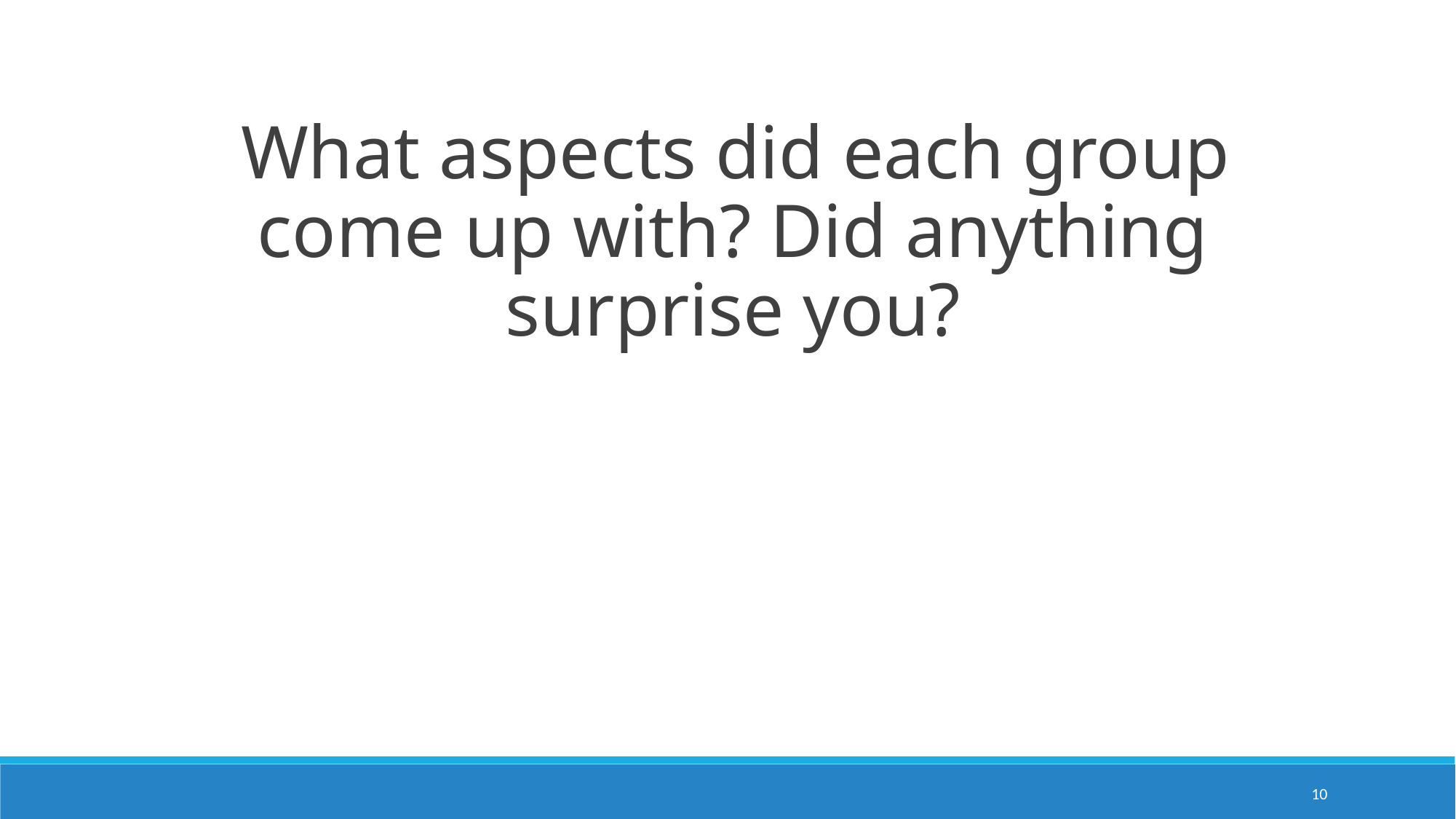

What aspects did each group come up with? Did anything surprise you?
10

## Slide 11
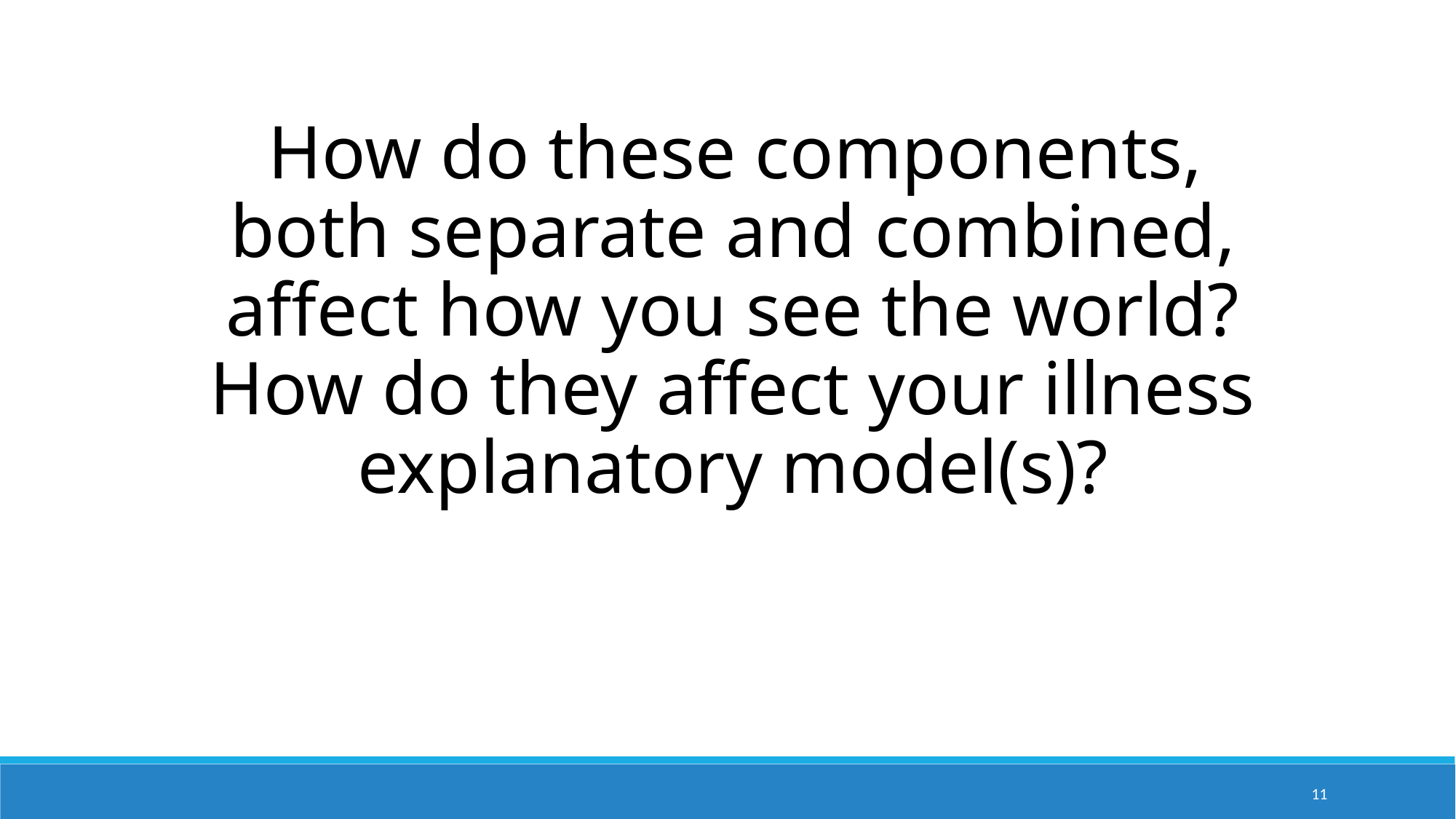

How do these components, both separate and combined, affect how you see the world? How do they affect your illness explanatory model(s)?
11

## Slide 12
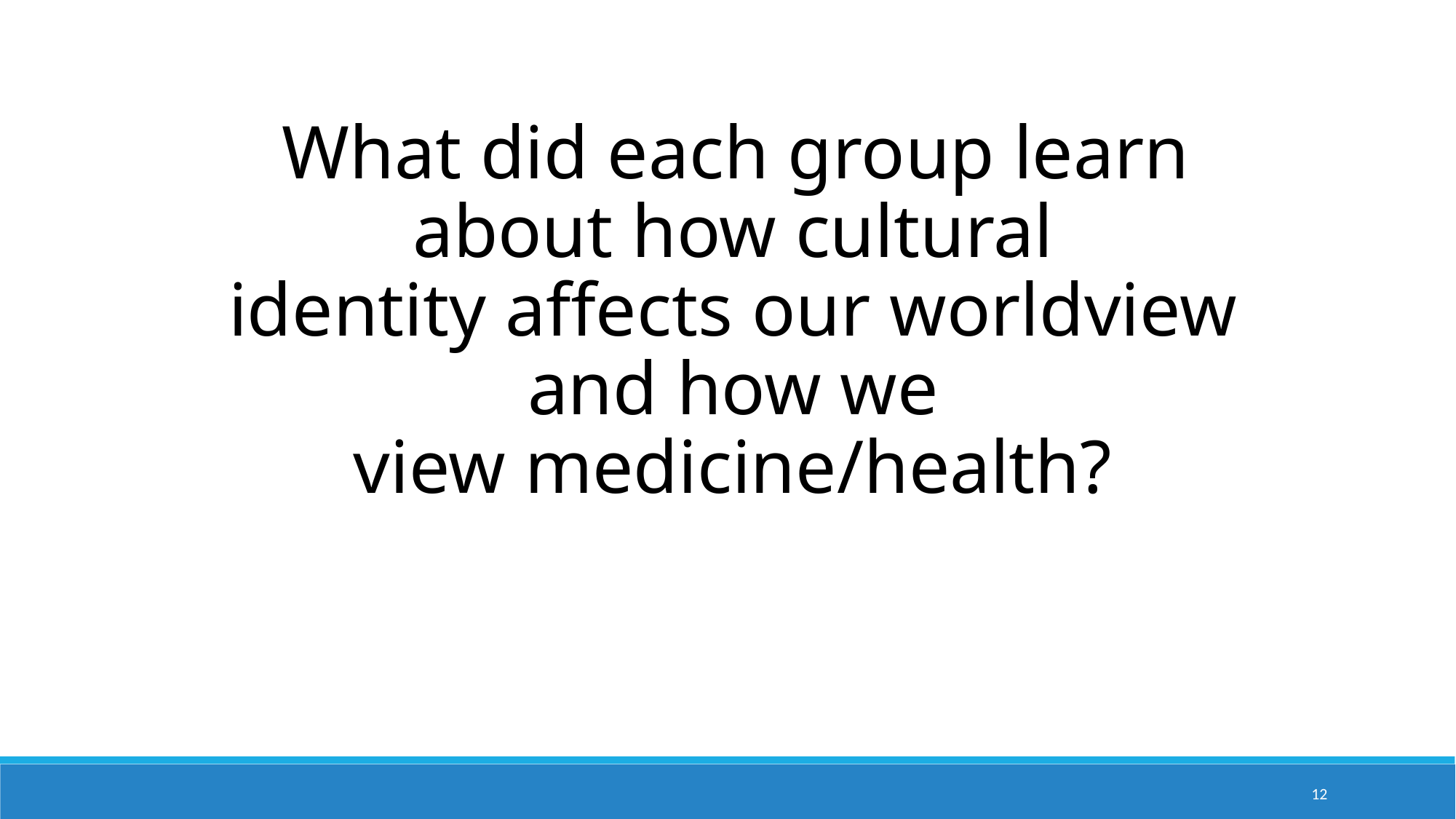

What did each group learn about how cultural identity affects our worldview and how we view medicine/health?
12

## Slide 13
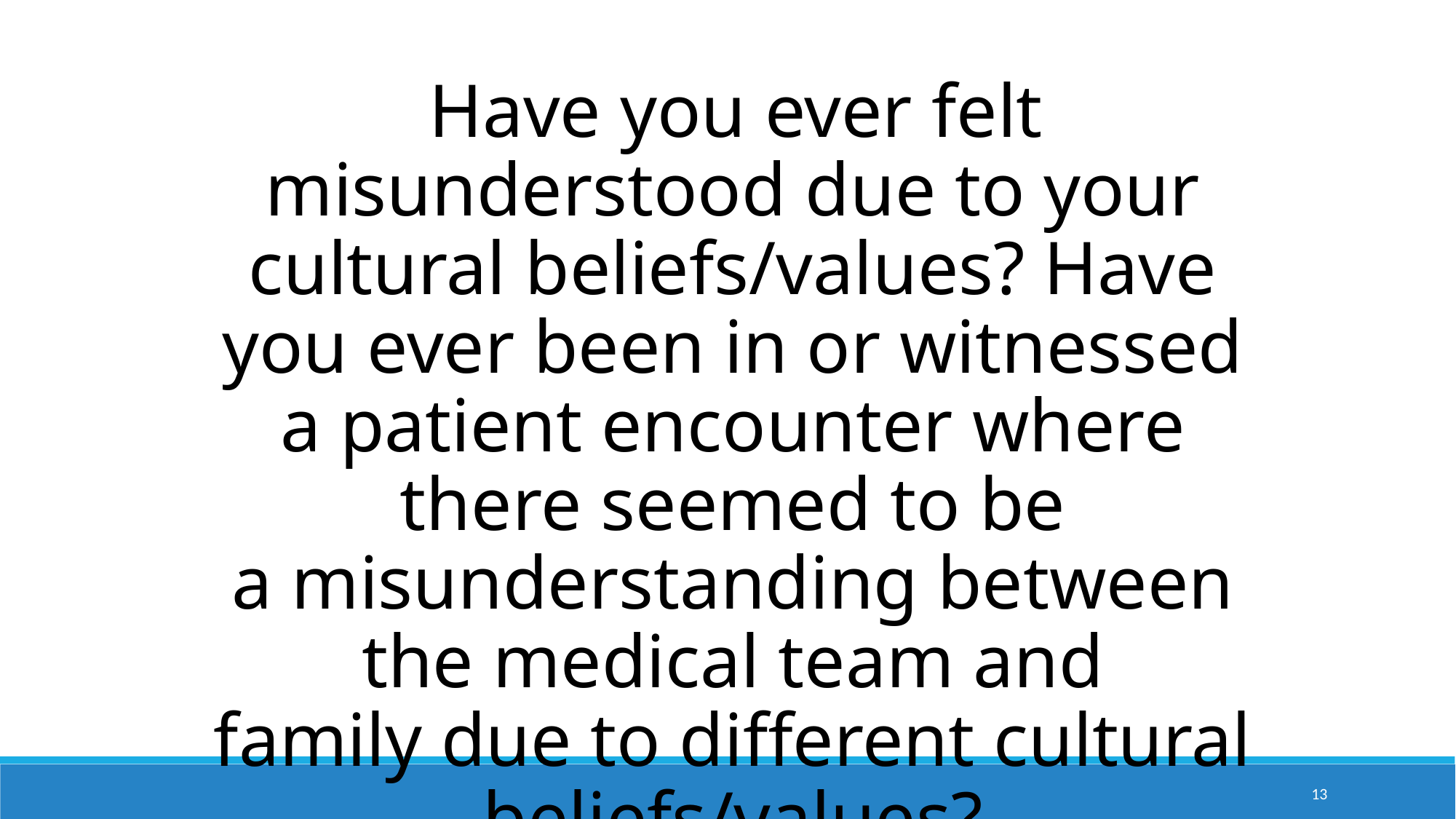

Have you ever felt misunderstood due to your cultural beliefs/values? Have you ever been in or witnessed a patient encounter where there seemed to be a misunderstanding between the medical team and family due to different cultural beliefs/values?
13

## Slide 14
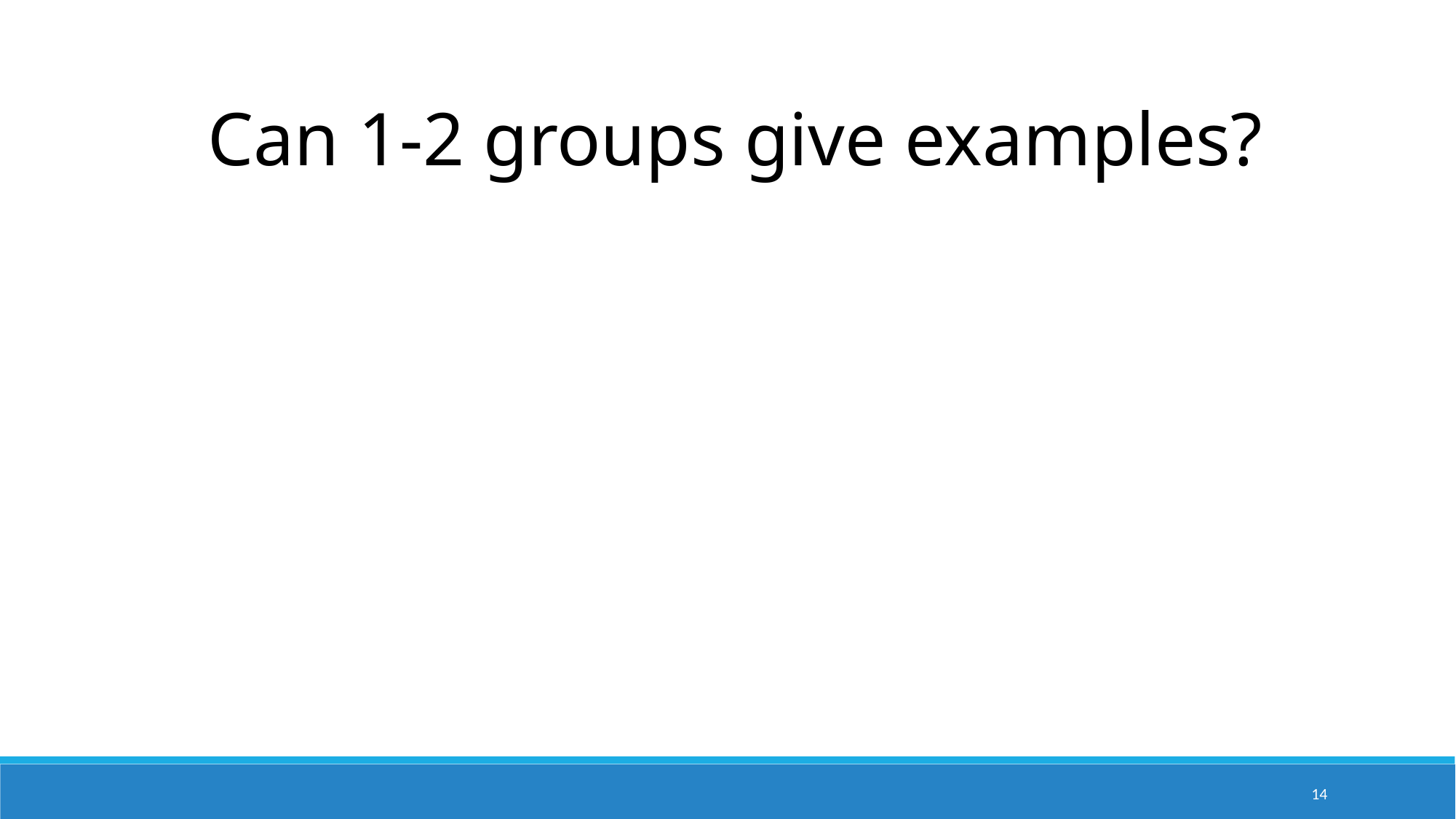

Can 1-2 groups give examples?
14

## Slide 15
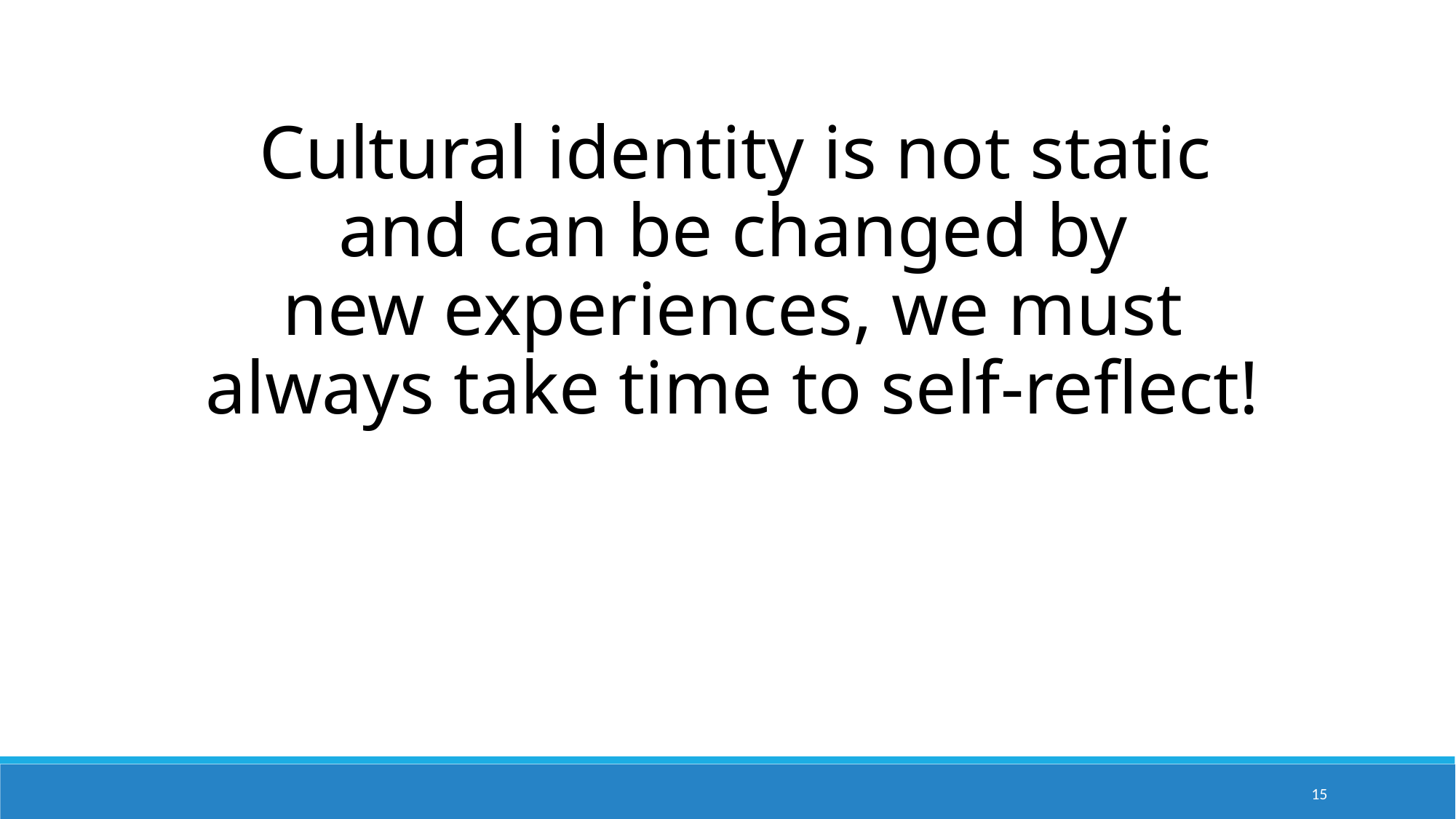

Cultural identity is not static and can be changed by new experiences, we must always take time to self-reflect!
15

## Slide 16
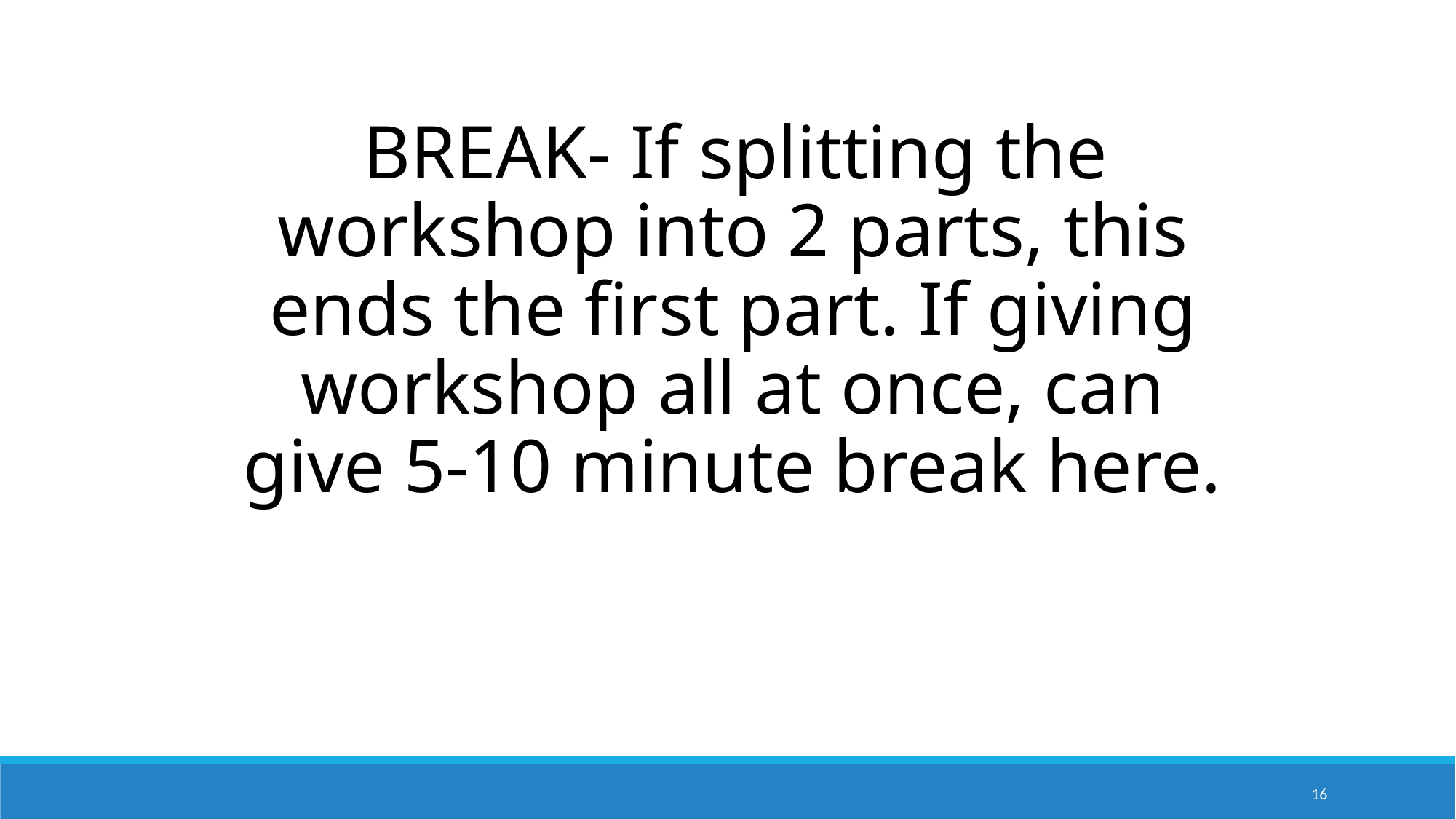

BREAK- If splitting the workshop into 2 parts, this ends the first part. If giving workshop all at once, can give 5-10 minute break here.
16

## Slide 17
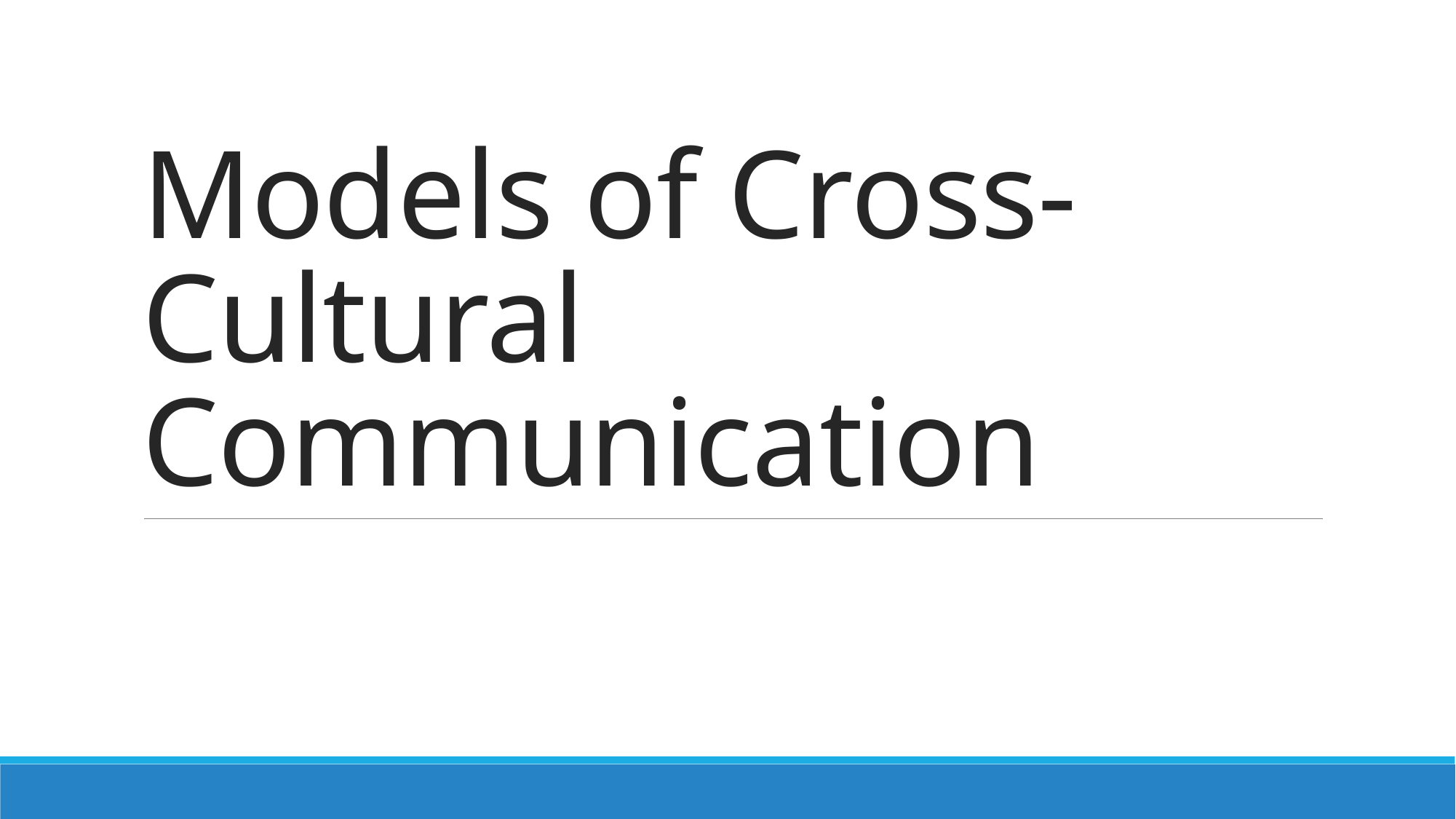

# Models of Cross-Cultural Communication

## Slide 18
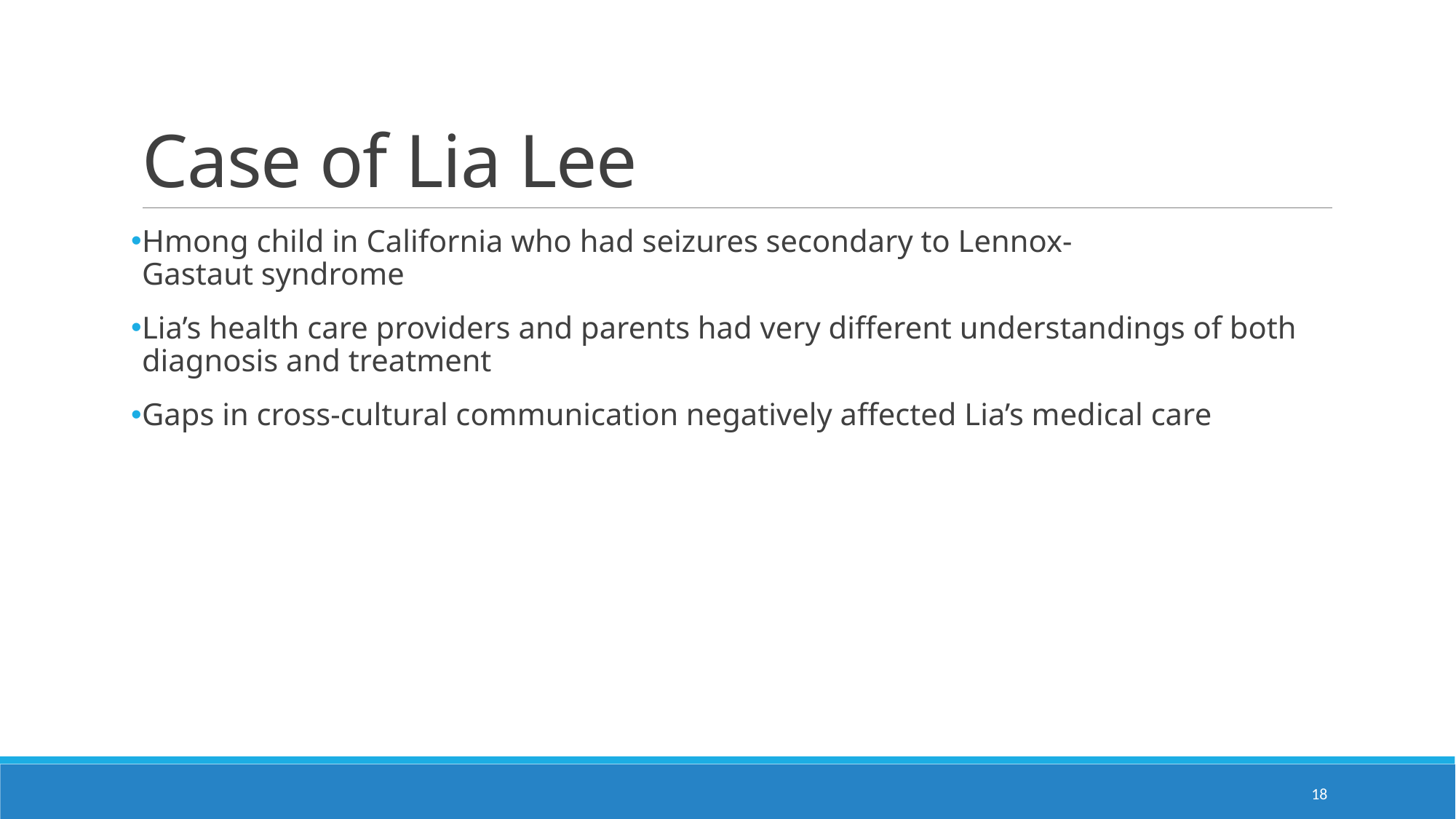

# Case of Lia Lee
Hmong child in California who had seizures secondary to Lennox-Gastaut syndrome​
Lia’s health care providers and parents had very different understandings of both diagnosis and treatment​
Gaps in cross-cultural communication negatively affected Lia’s medical care​
18

## Slide 19
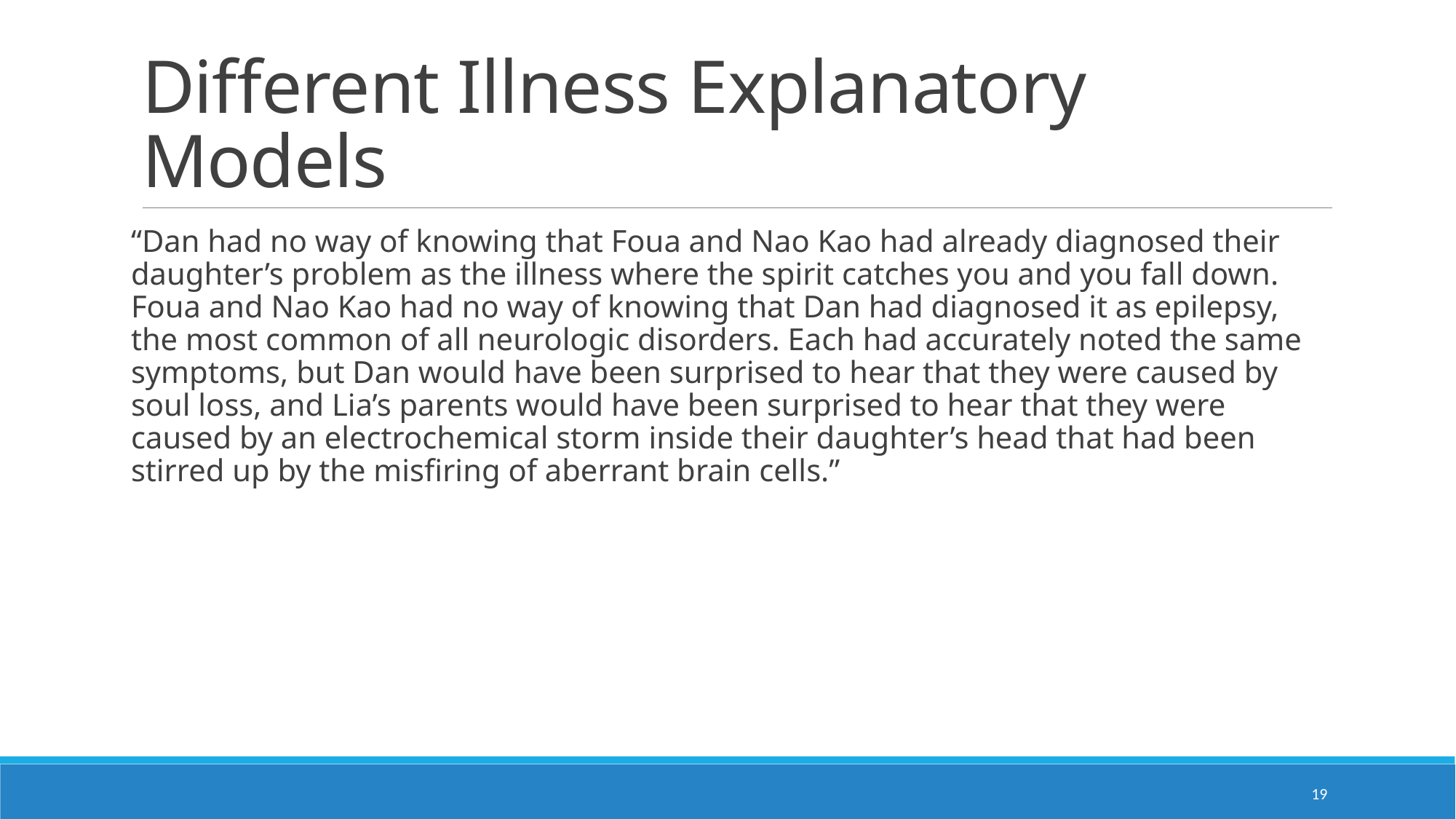

# Different Illness Explanatory Models
“Dan had no way of knowing that Foua and Nao Kao had already diagnosed their daughter’s problem as the illness where the spirit catches you and you fall down. Foua and Nao Kao had no way of knowing that Dan had diagnosed it as epilepsy, the most common of all neurologic disorders. Each had accurately noted the same symptoms, but Dan would have been surprised to hear that they were caused by soul loss, and Lia’s parents would have been surprised to hear that they were caused by an electrochemical storm inside their daughter’s head that had been stirred up by the misfiring of aberrant brain cells.”
19

## Slide 20
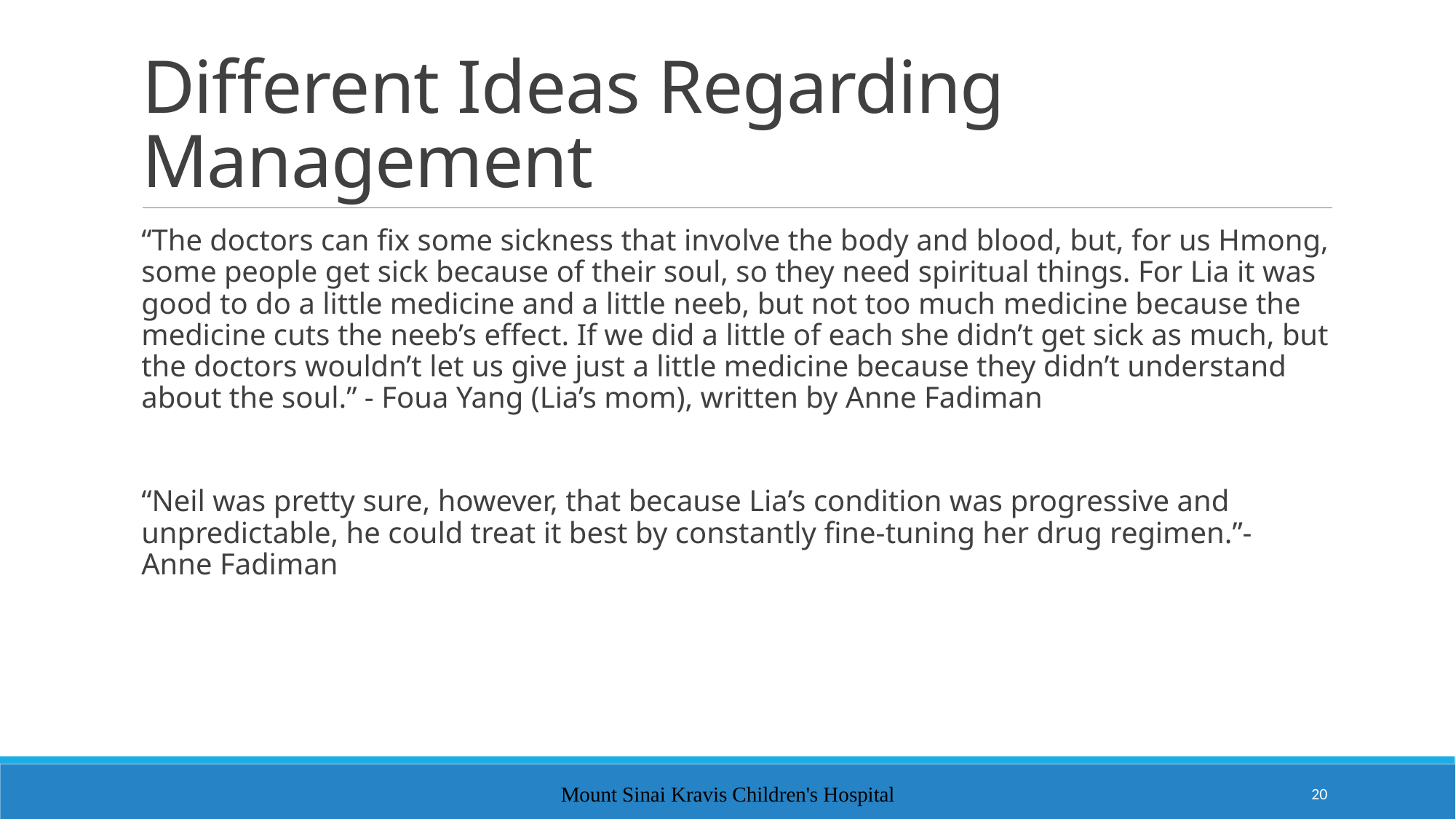

# Different Ideas Regarding Management
“The doctors can fix some sickness that involve the body and blood, but, for us Hmong, some people get sick because of their soul, so they need spiritual things. For Lia it was good to do a little medicine and a little neeb, but not too much medicine because the medicine cuts the neeb’s effect. If we did a little of each she didn’t get sick as much, but the doctors wouldn’t let us give just a little medicine because they didn’t understand about the soul.” - Foua Yang (Lia’s mom), written by Anne Fadiman​
​
“Neil was pretty sure, however, that because Lia’s condition was progressive and unpredictable, he could treat it best by constantly fine-tuning her drug regimen.”- Anne Fadiman​
​
 ​
Mount Sinai Kravis Children's Hospital
20

## Slide 21
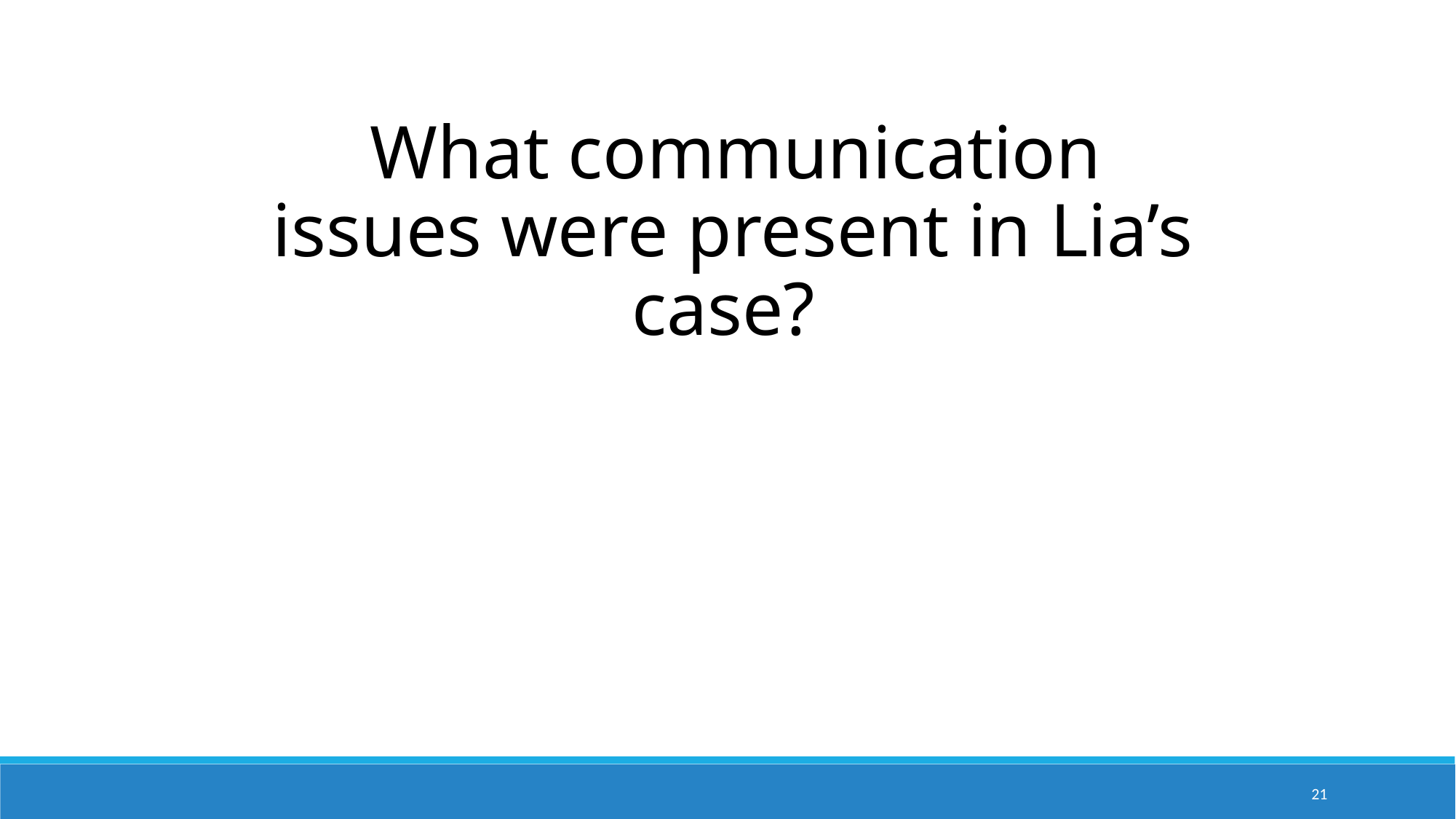

What communication issues were present in Lia’s case?
21

## Slide 22
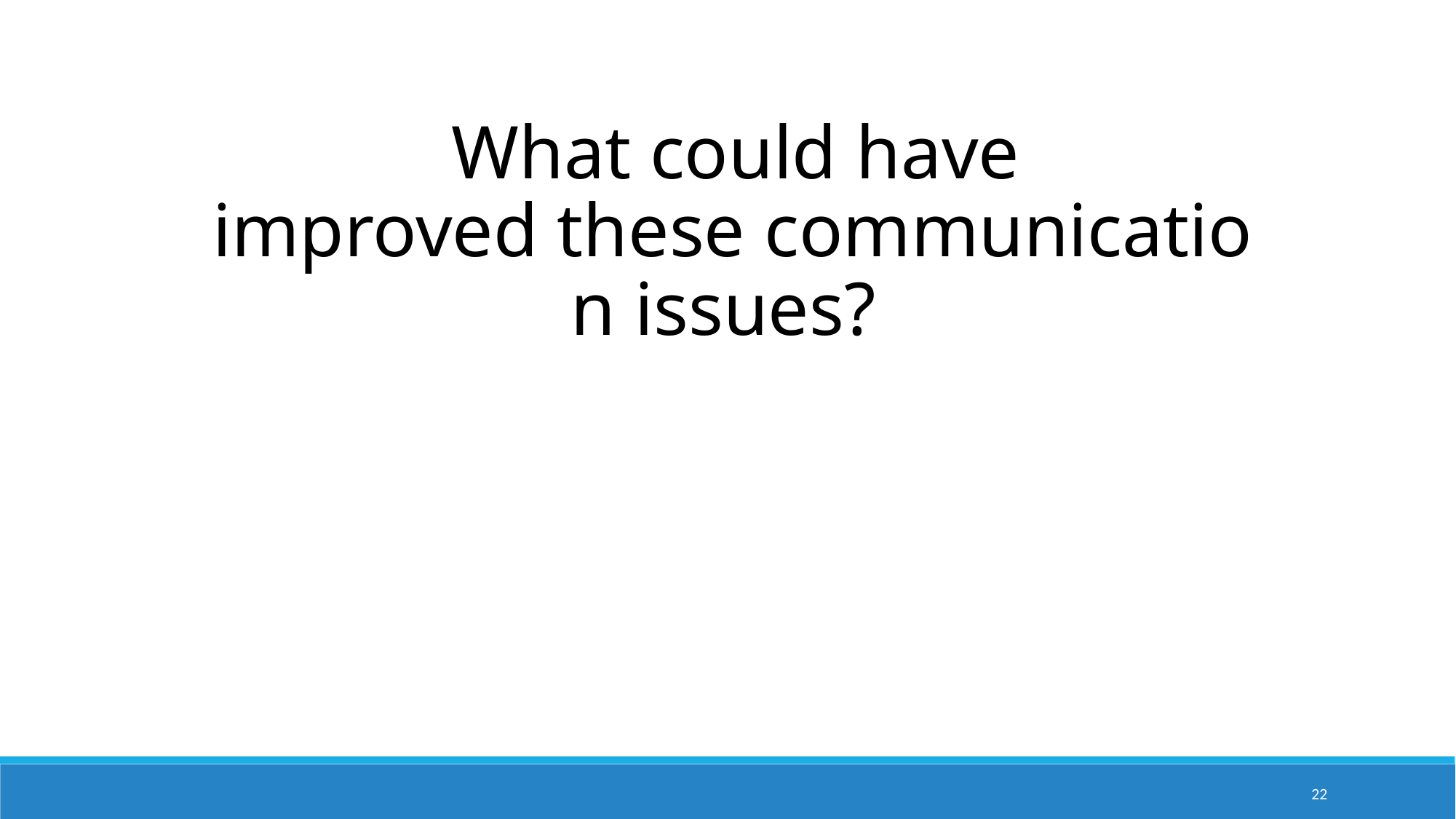

What could have improved these communication issues?
22

## Slide 23
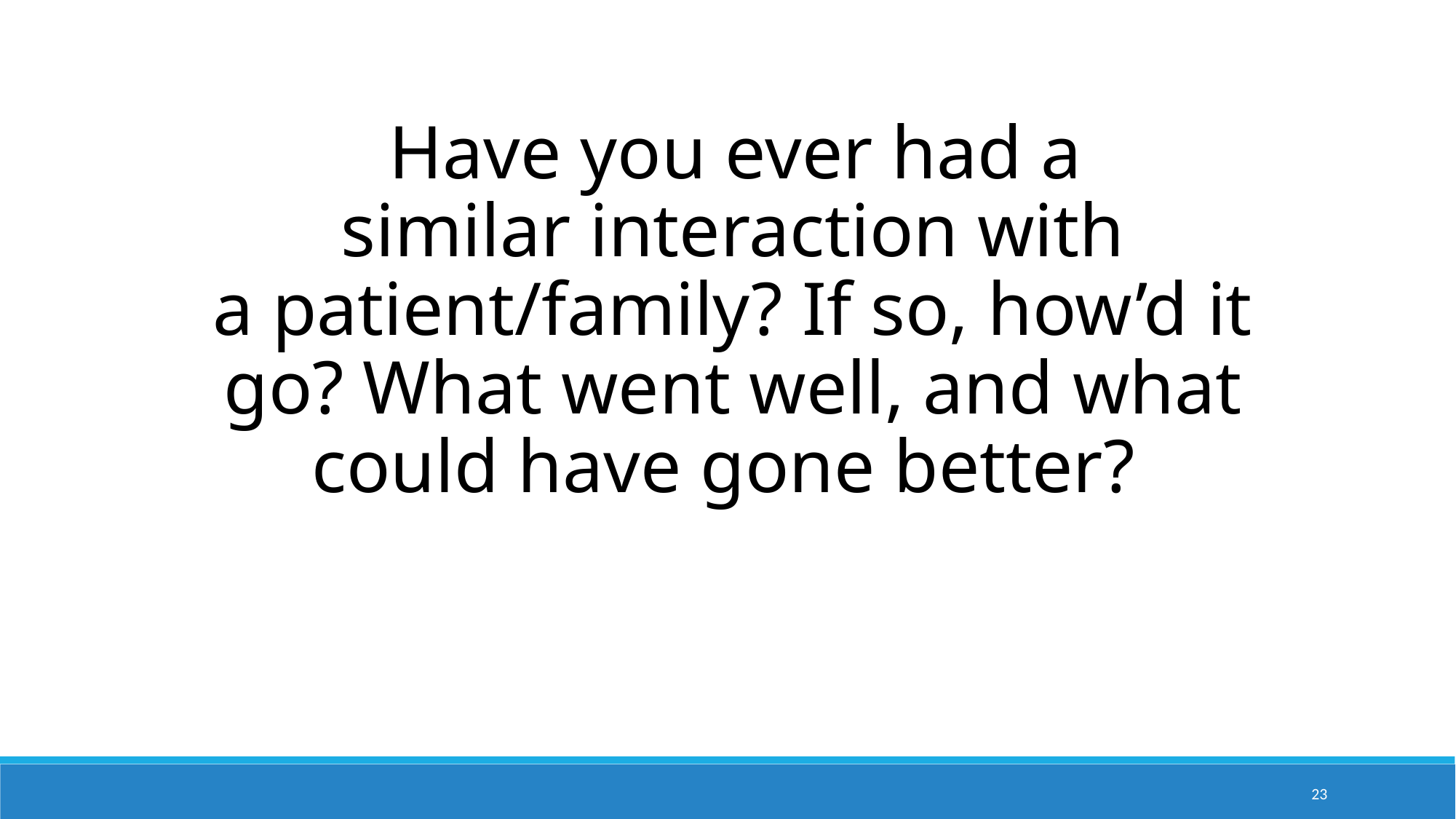

Have you ever had a similar interaction with a patient/family? If so, how’d it go? What went well, and what could have gone better?
23

## Slide 24
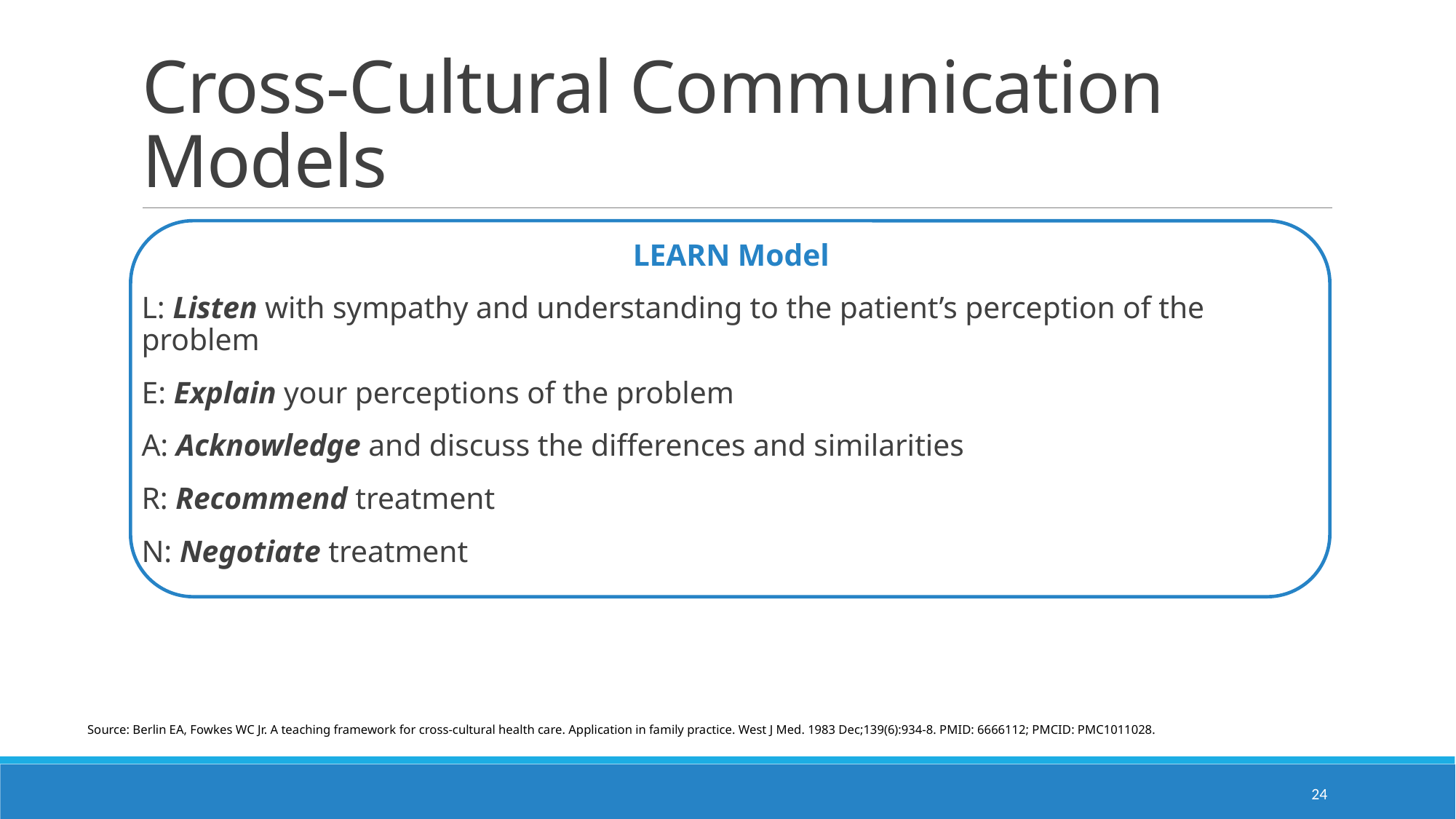

# Cross-Cultural Communication Models
LEARN Model
L: Listen with sympathy and understanding to the patient’s perception of the problem​
E: Explain your perceptions of the problem​
A: Acknowledge and discuss the differences and similarities​
R: Recommend treatment​
N: Negotiate treatment
Source: Berlin EA, Fowkes WC Jr. A teaching framework for cross-cultural health care. Application in family practice. West J Med. 1983 Dec;139(6):934-8. PMID: 6666112; PMCID: PMC1011028.
24

## Slide 25
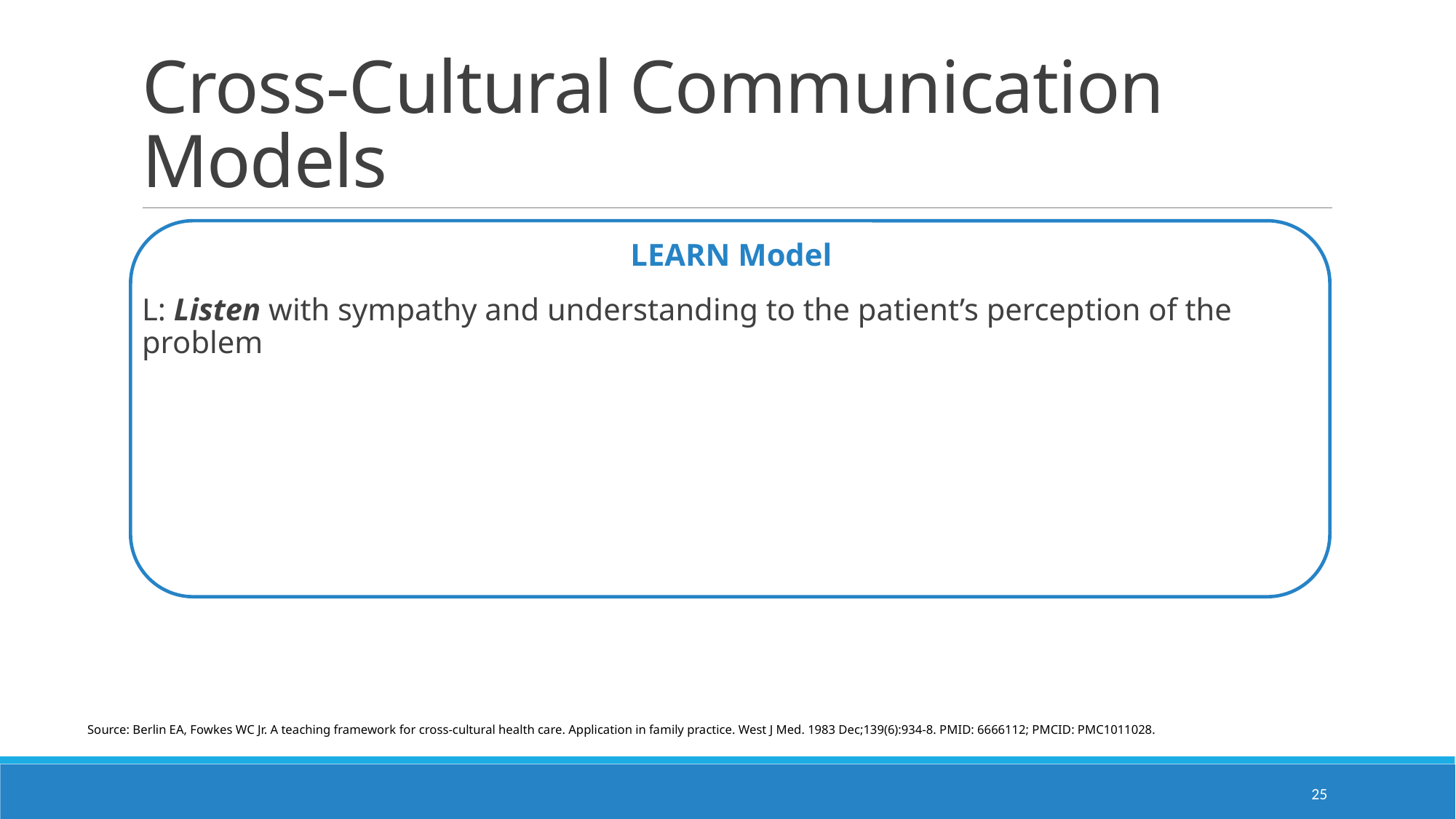

# Cross-Cultural Communication Models
LEARN Model
L: Listen with sympathy and understanding to the patient’s perception of the problem​
Source: Berlin EA, Fowkes WC Jr. A teaching framework for cross-cultural health care. Application in family practice. West J Med. 1983 Dec;139(6):934-8. PMID: 6666112; PMCID: PMC1011028.
25

## Slide 26
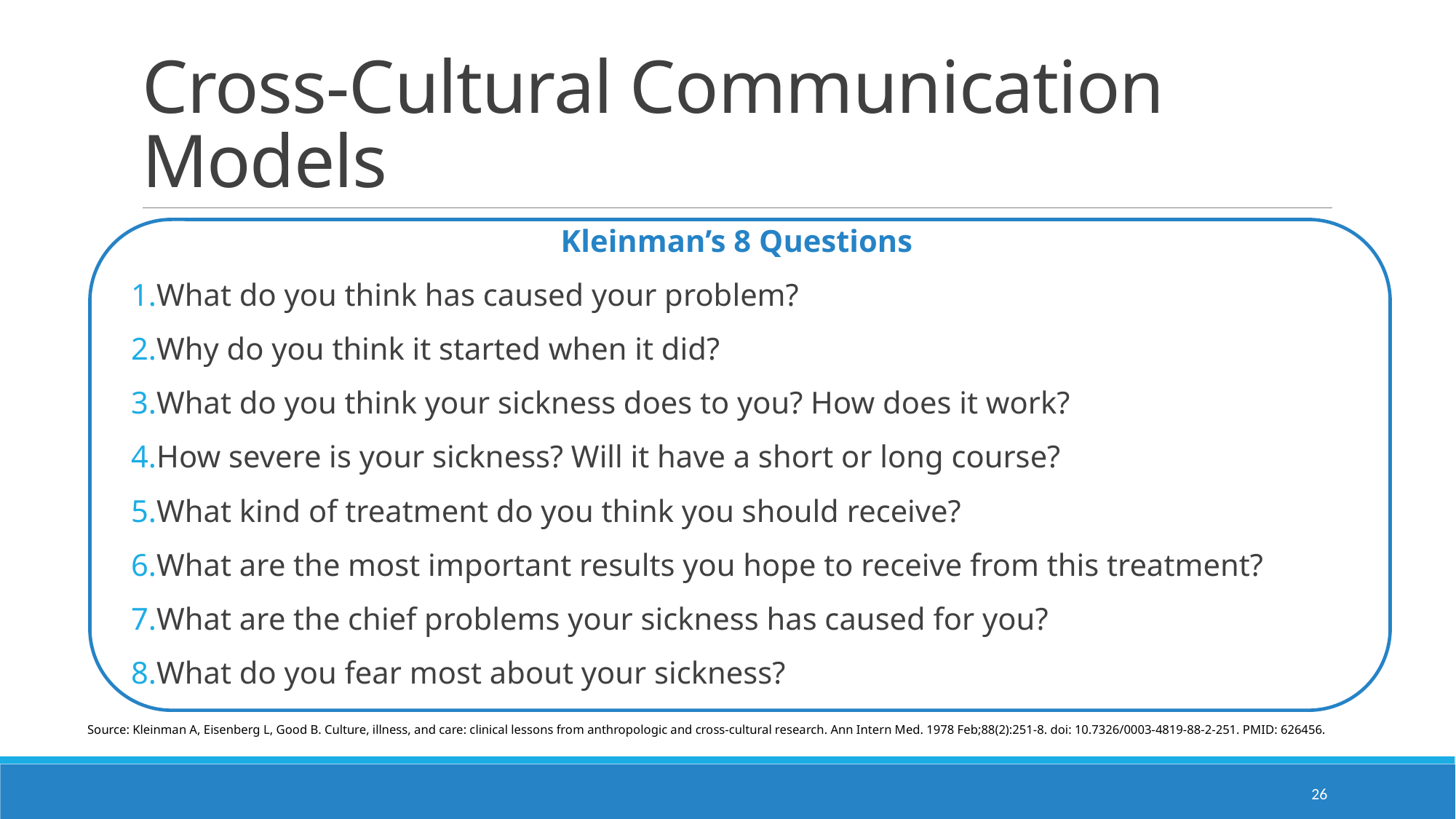

# Cross-Cultural Communication Models
Kleinman’s 8 Questions
What do you think has caused your problem?​
Why do you think it started when it did?​
What do you think your sickness does to you? How does it work?​
How severe is your sickness? Will it have a short or long course?​
What kind of treatment do you think you should receive?​
What are the most important results you hope to receive from this treatment?​
What are the chief problems your sickness has caused for you?​
What do you fear most about your sickness?
Source: Kleinman A, Eisenberg L, Good B. Culture, illness, and care: clinical lessons from anthropologic and cross-cultural research. Ann Intern Med. 1978 Feb;88(2):251-8. doi: 10.7326/0003-4819-88-2-251. PMID: 626456.​
26

## Slide 27
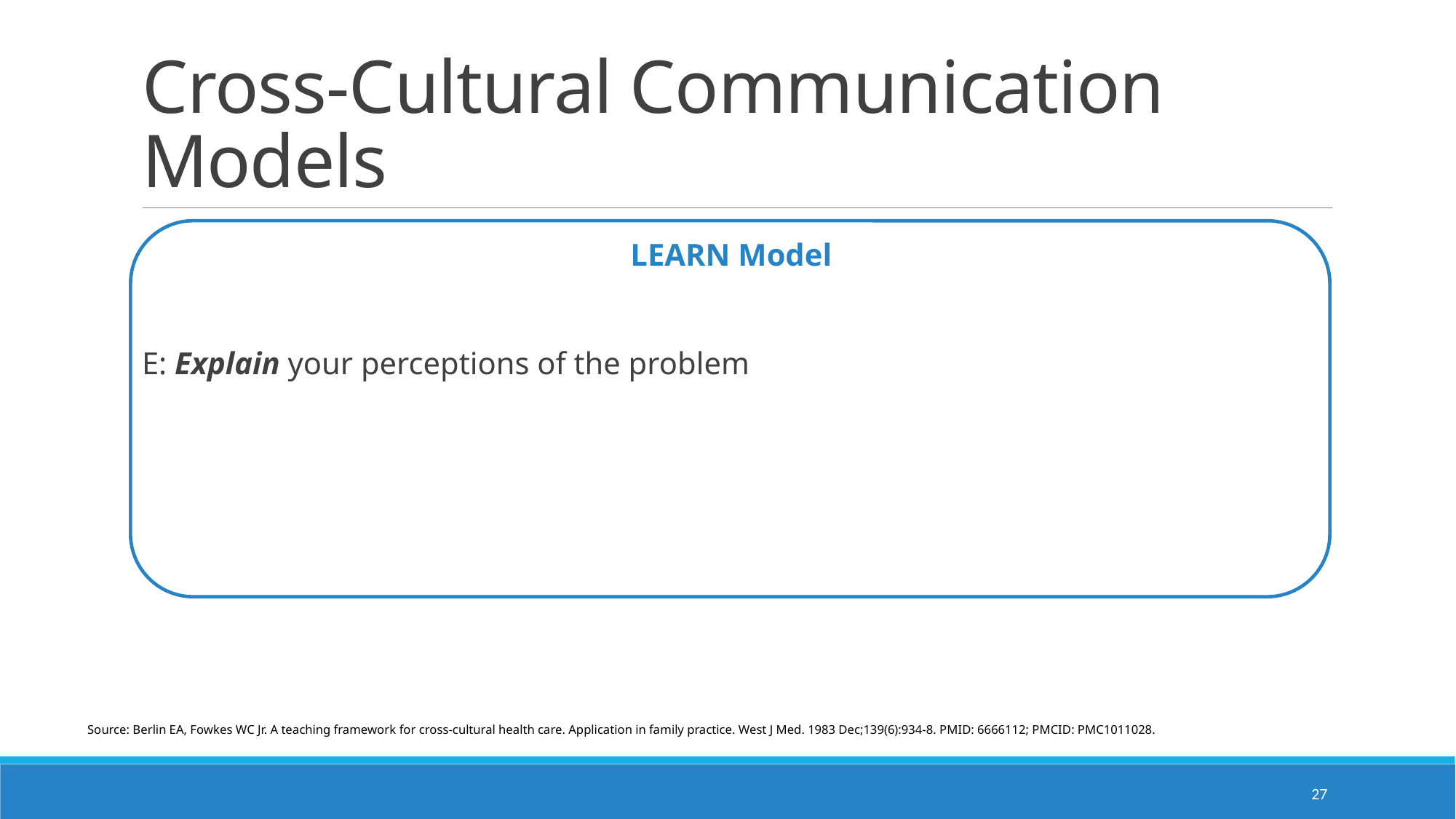

# Cross-Cultural Communication Models
LEARN Model
E: Explain your perceptions of the problem​
Source: Berlin EA, Fowkes WC Jr. A teaching framework for cross-cultural health care. Application in family practice. West J Med. 1983 Dec;139(6):934-8. PMID: 6666112; PMCID: PMC1011028.
27

## Slide 28
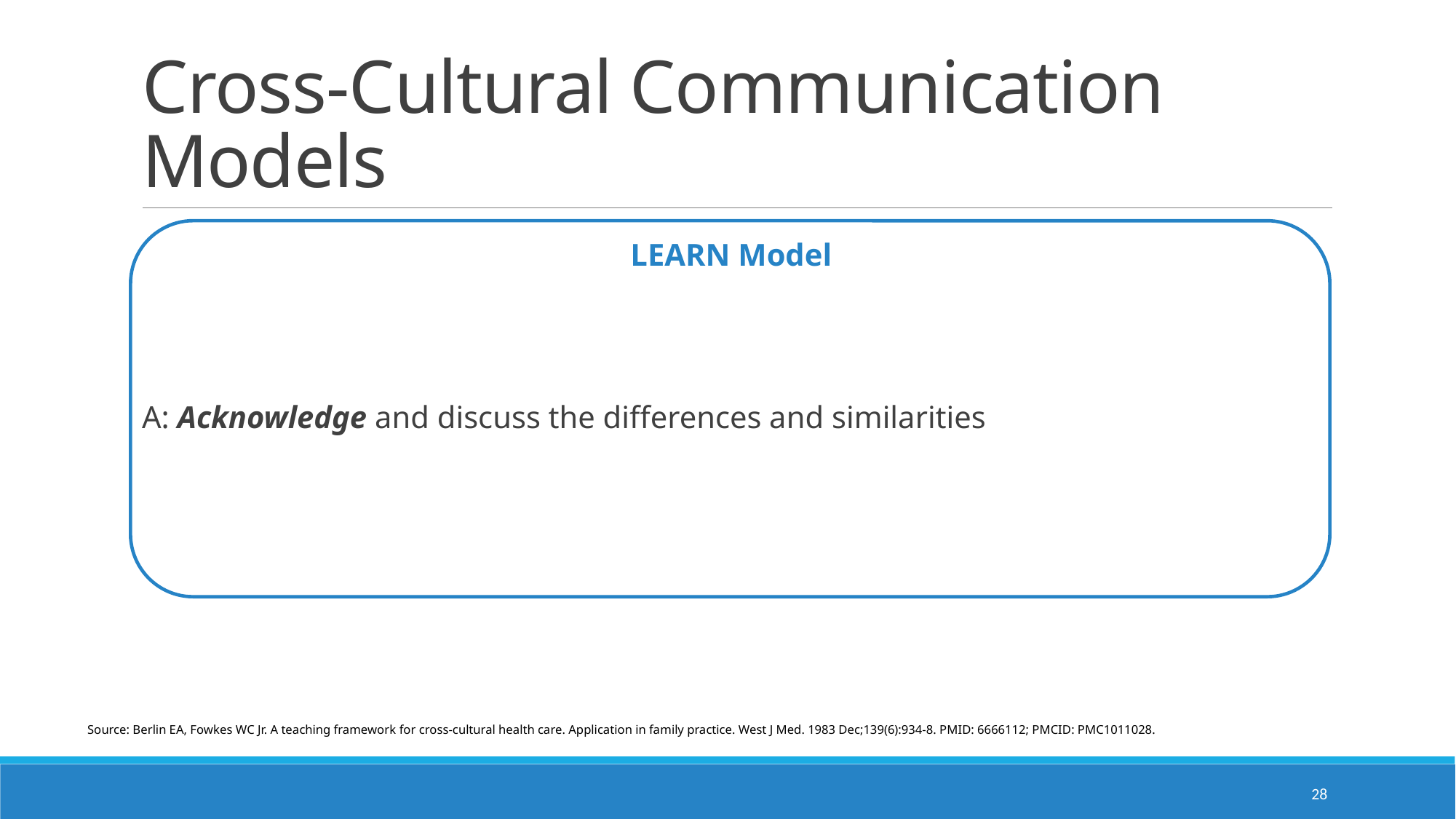

# Cross-Cultural Communication Models
LEARN Model
A: Acknowledge and discuss the differences and similarities​
Source: Berlin EA, Fowkes WC Jr. A teaching framework for cross-cultural health care. Application in family practice. West J Med. 1983 Dec;139(6):934-8. PMID: 6666112; PMCID: PMC1011028.
28

## Slide 29
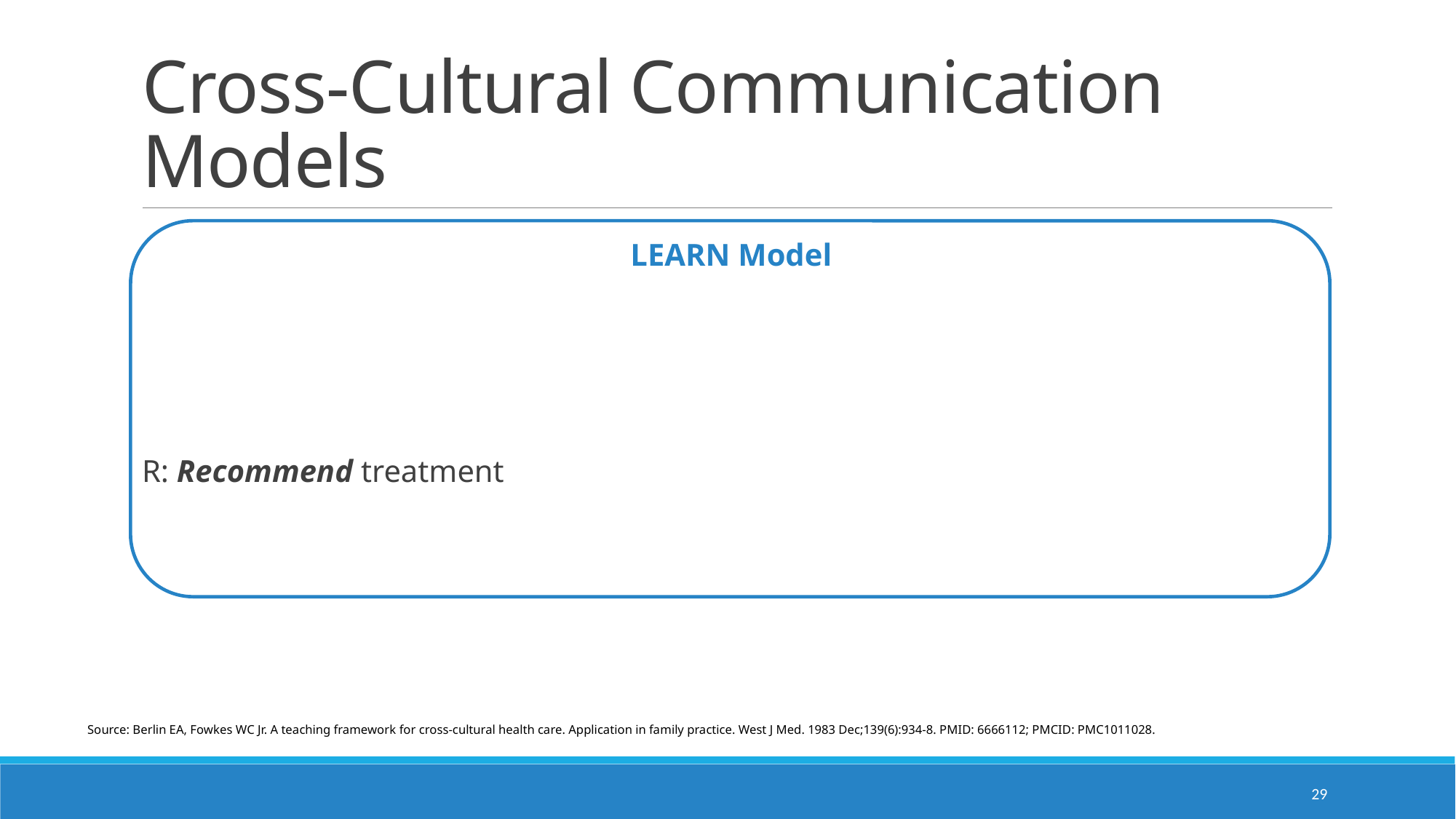

# Cross-Cultural Communication Models
LEARN Model
R: Recommend treatment​
Source: Berlin EA, Fowkes WC Jr. A teaching framework for cross-cultural health care. Application in family practice. West J Med. 1983 Dec;139(6):934-8. PMID: 6666112; PMCID: PMC1011028.
29

## Slide 30
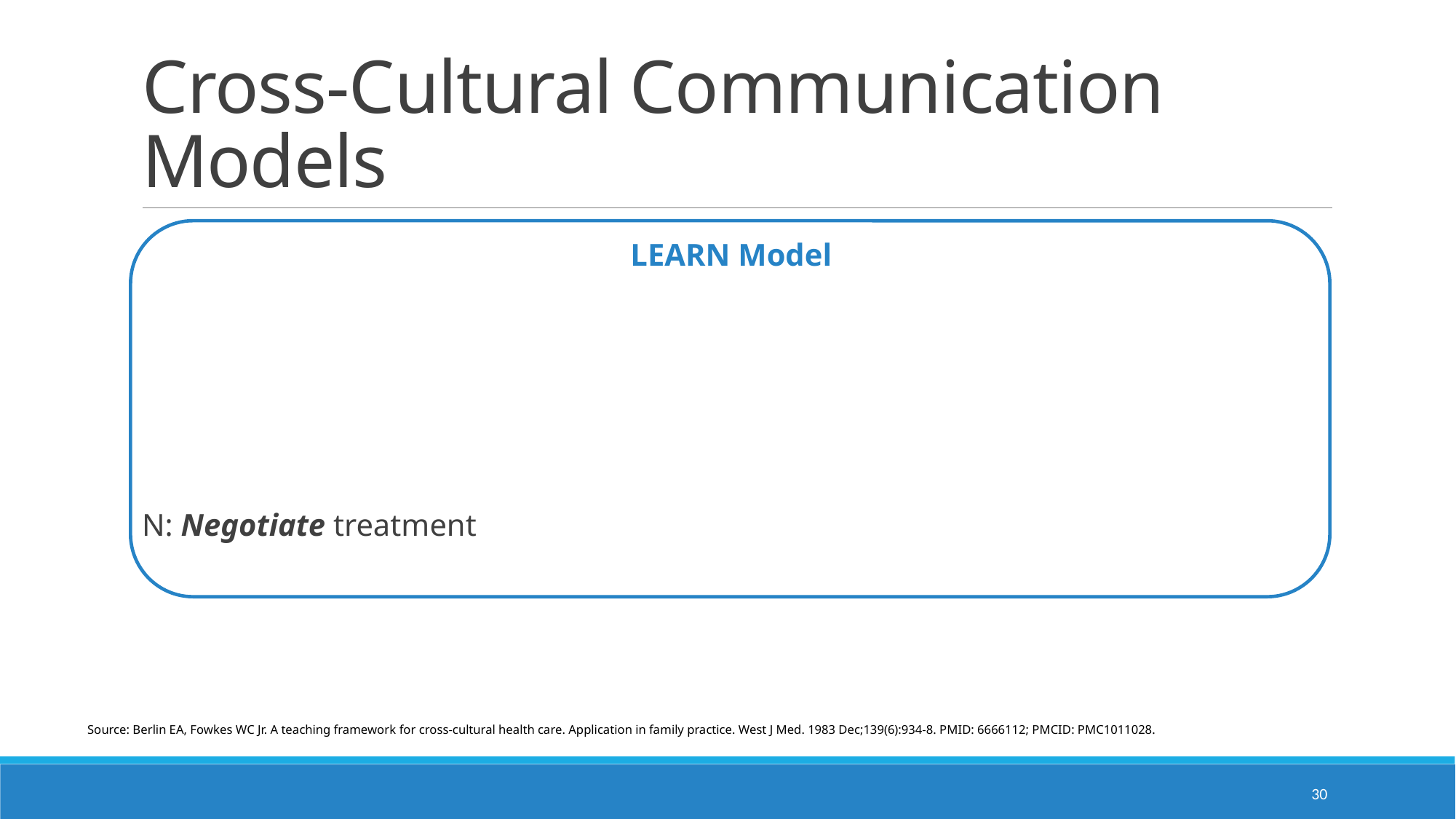

# Cross-Cultural Communication Models
LEARN Model
N: Negotiate treatment
Source: Berlin EA, Fowkes WC Jr. A teaching framework for cross-cultural health care. Application in family practice. West J Med. 1983 Dec;139(6):934-8. PMID: 6666112; PMCID: PMC1011028.
30

## Slide 31
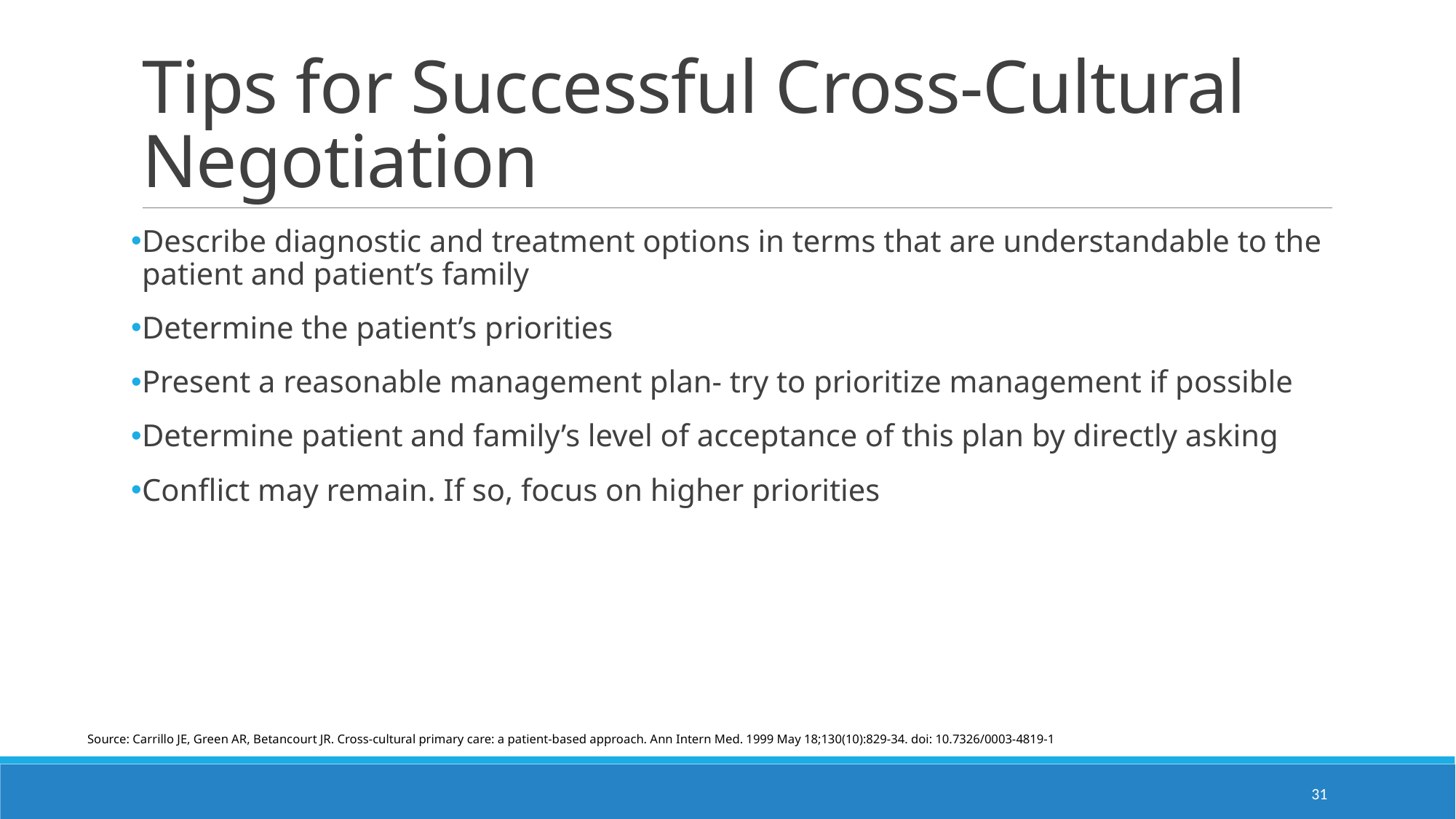

# Tips for Successful Cross-Cultural Negotiation
Describe diagnostic and treatment options in terms that are understandable to the patient and patient’s family​
Determine the patient’s priorities​
Present a reasonable management plan- try to prioritize management if possible​
Determine patient and family’s level of acceptance of this plan by directly asking​
Conflict may remain. If so, focus on higher priorities
Source: Carrillo JE, Green AR, Betancourt JR. Cross-cultural primary care: a patient-based approach. Ann Intern Med. 1999 May 18;130(10):829-34. doi: 10.7326/0003-4819-130-10-199905180-00017. PMID: 10366373.
31

## Slide 32
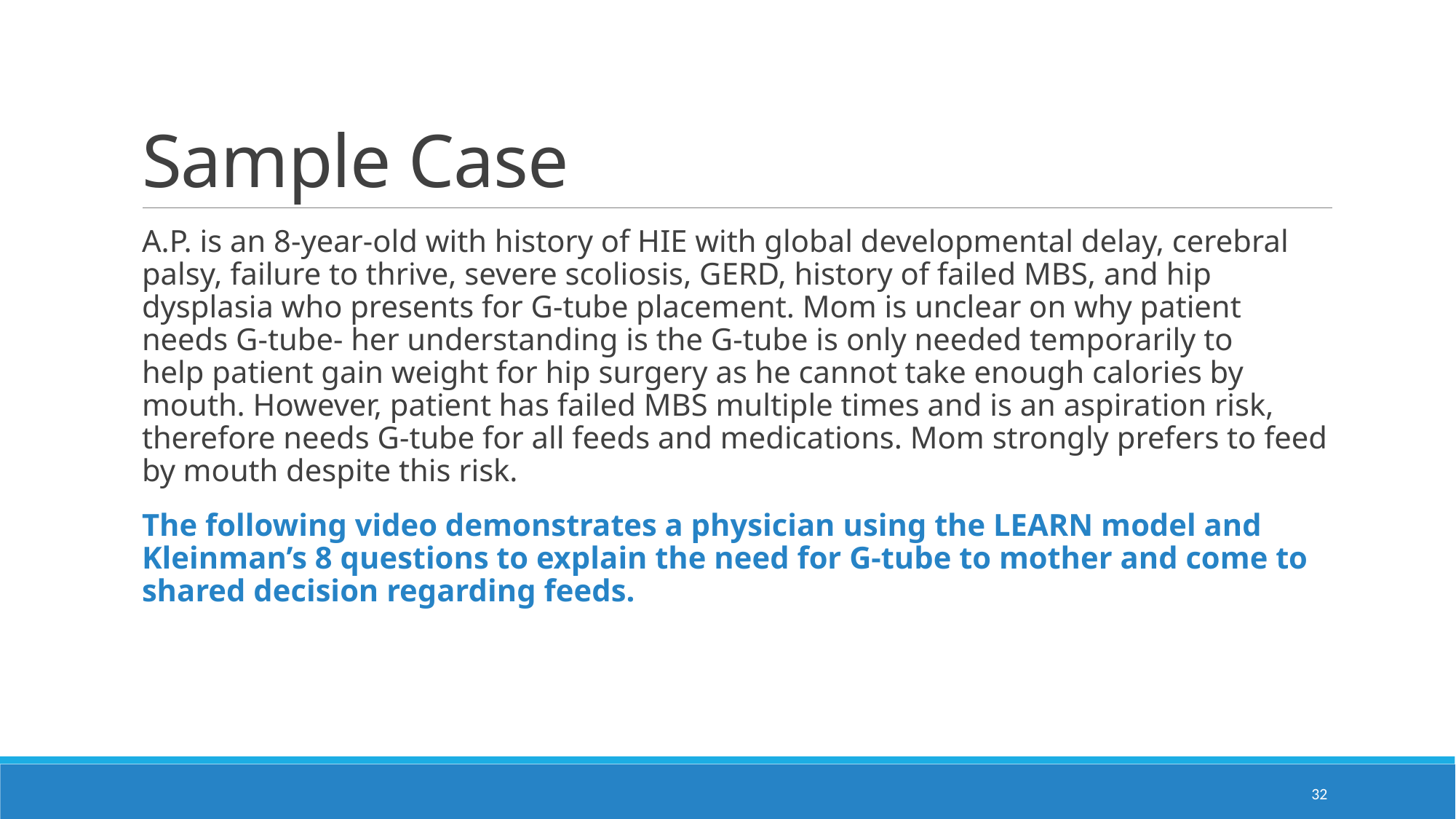

# Sample Case
A.P. is an 8-year-old with history of HIE with global developmental delay, cerebral palsy, failure to thrive, severe scoliosis, GERD, history of failed MBS, and hip dysplasia who presents for G-tube placement. Mom is unclear on why patient needs G-tube- her understanding is the G-tube is only needed temporarily to help patient gain weight for hip surgery as he cannot take enough calories by mouth. However, patient has failed MBS multiple times and is an aspiration risk, therefore needs G-tube for all feeds and medications. Mom strongly prefers to feed by mouth despite this risk. ​
The following video demonstrates a physician using the LEARN model and Kleinman’s 8 questions to explain the need for G-tube to mother and come to shared decision regarding feeds.
32

## Slide 33
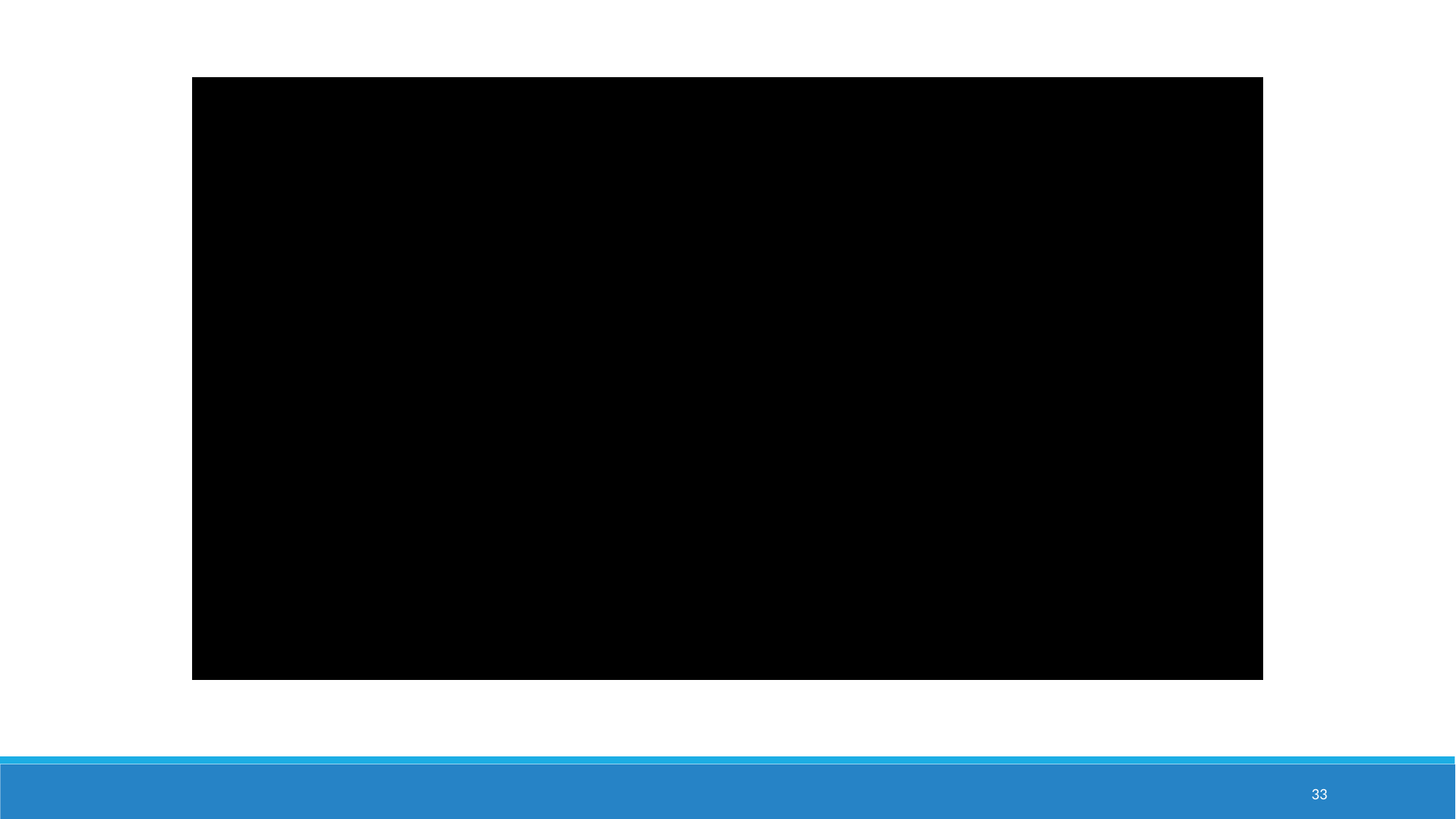

33

## Slide 34
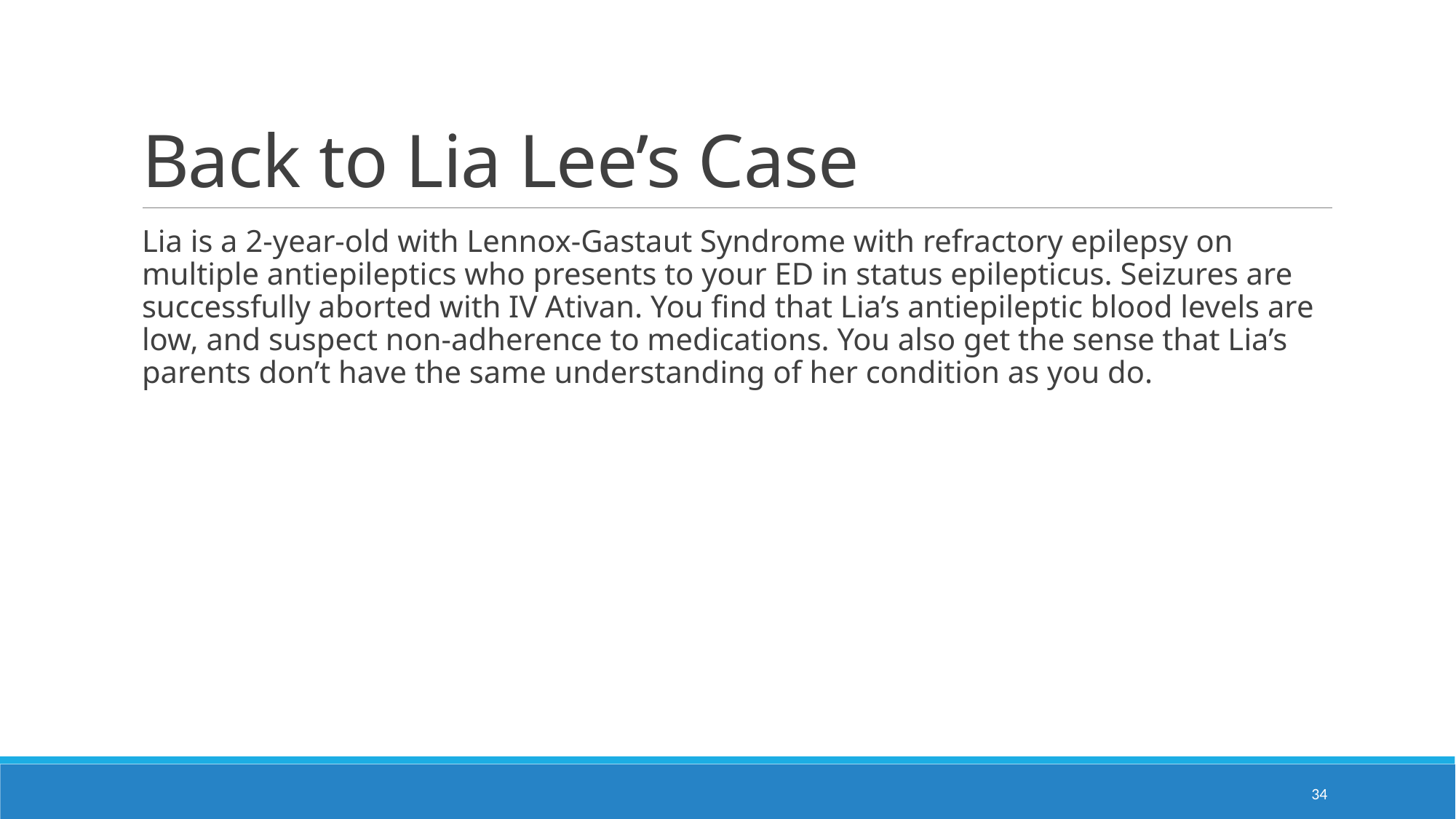

# Back to Lia Lee’s Case
Lia is a 2-year-old with Lennox-Gastaut Syndrome with refractory epilepsy on multiple antiepileptics who presents to your ED in status epilepticus. Seizures are successfully aborted with IV Ativan. You find that Lia’s antiepileptic blood levels are low, and suspect non-adherence to medications. You also get the sense that Lia’s parents don’t have the same understanding of her condition as you do.
34

## Slide 35
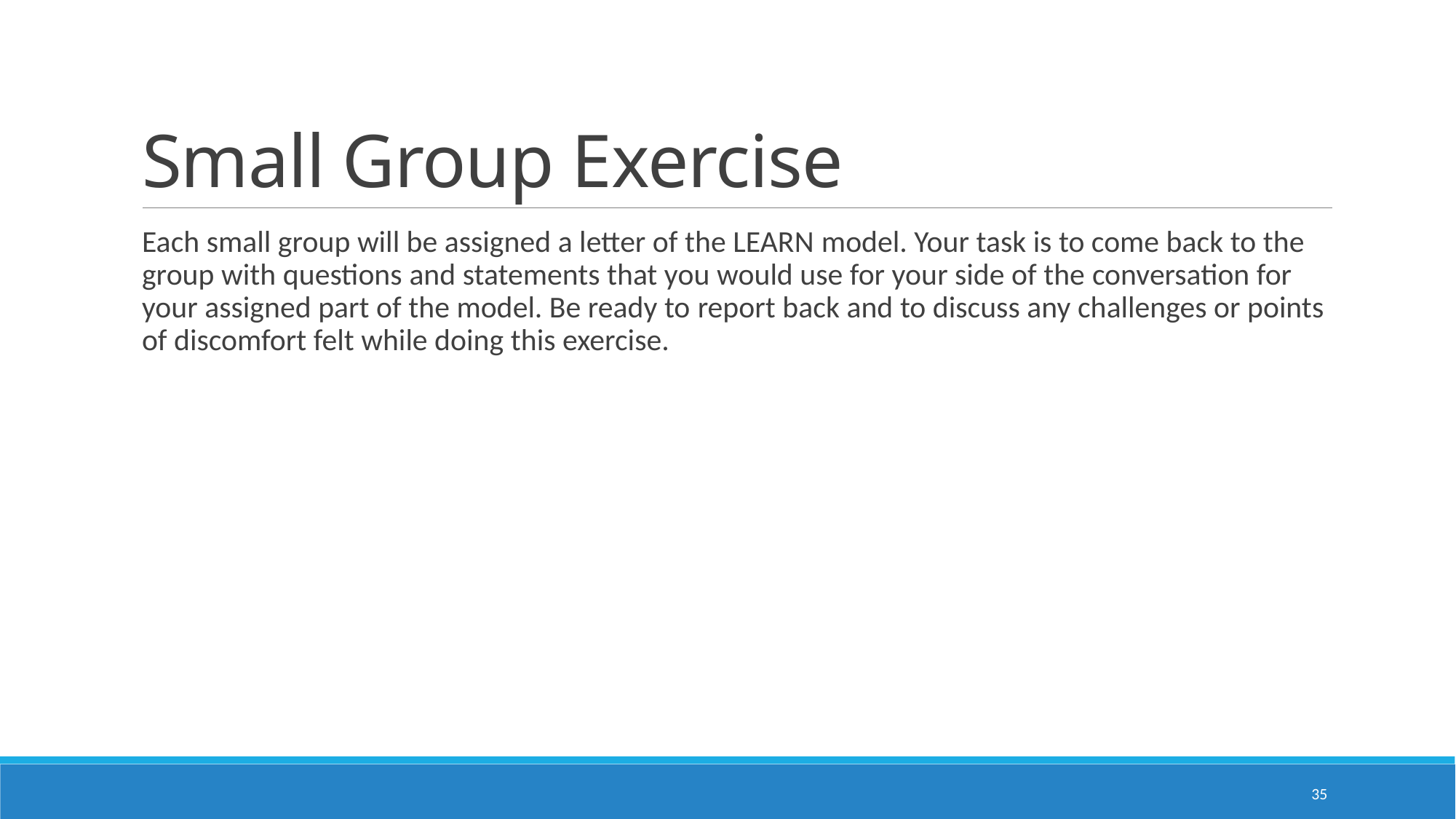

# Small Group Exercise
Each small group will be assigned a letter of the LEARN model. Your task is to come back to the group with questions and statements that you would use for your side of the conversation for your assigned part of the model. Be ready to report back and to discuss any challenges or points of discomfort felt while doing this exercise.
35

## Slide 36
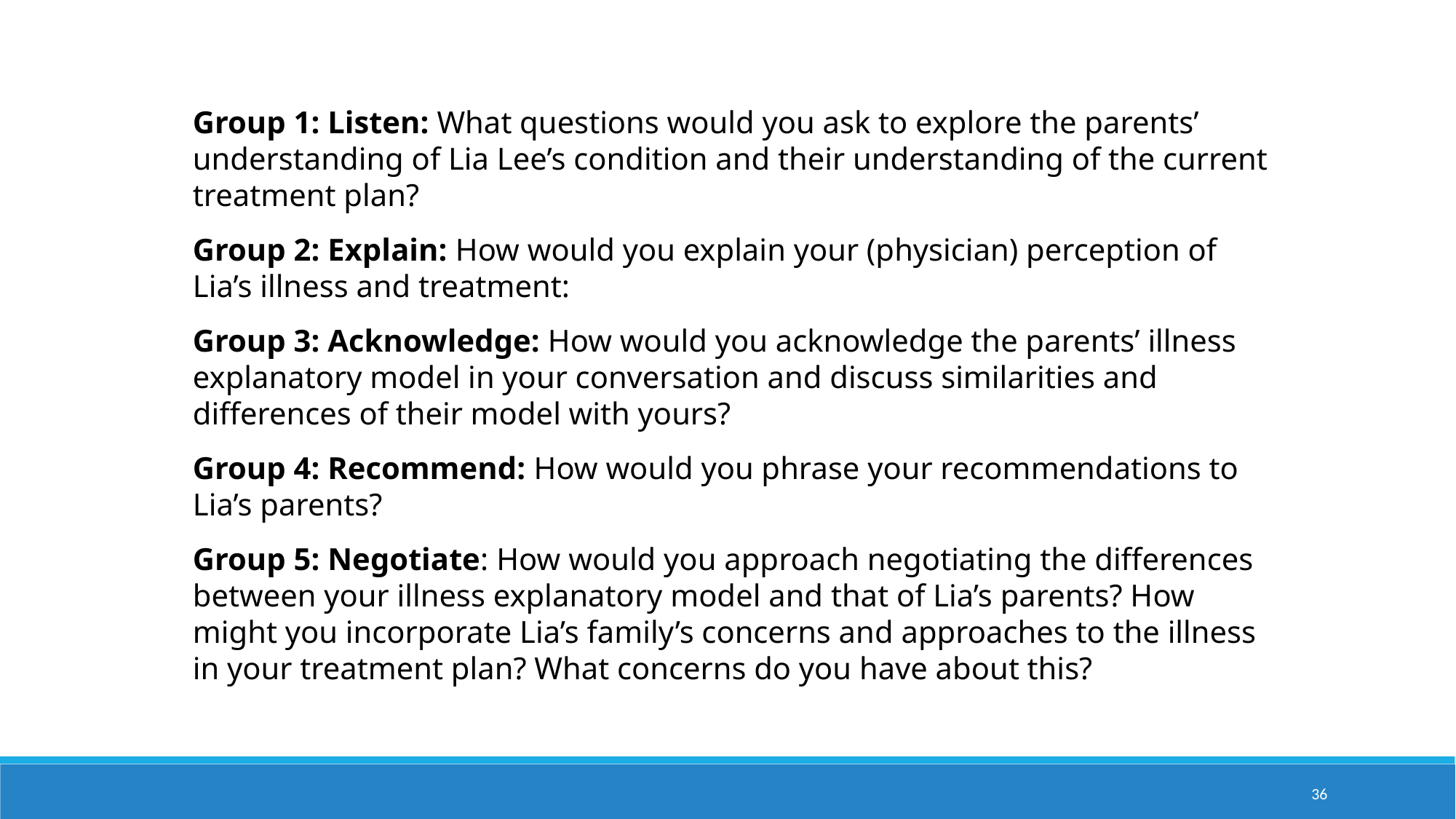

Group 1: Listen: What questions would you ask to explore the parents’ understanding of Lia Lee’s condition and their understanding of the current treatment plan?
Group 2: Explain: How would you explain your (physician) perception of Lia’s illness and treatment:
Group 3: Acknowledge: How would you acknowledge the parents’ illness explanatory model in your conversation and discuss similarities and differences of their model with yours?
Group 4: Recommend: How would you phrase your recommendations to Lia’s parents?
Group 5: Negotiate: How would you approach negotiating the differences between your illness explanatory model and that of Lia’s parents? How might you incorporate Lia’s family’s concerns and approaches to the illness in your treatment plan? What concerns do you have about this?
36

## Slide 37
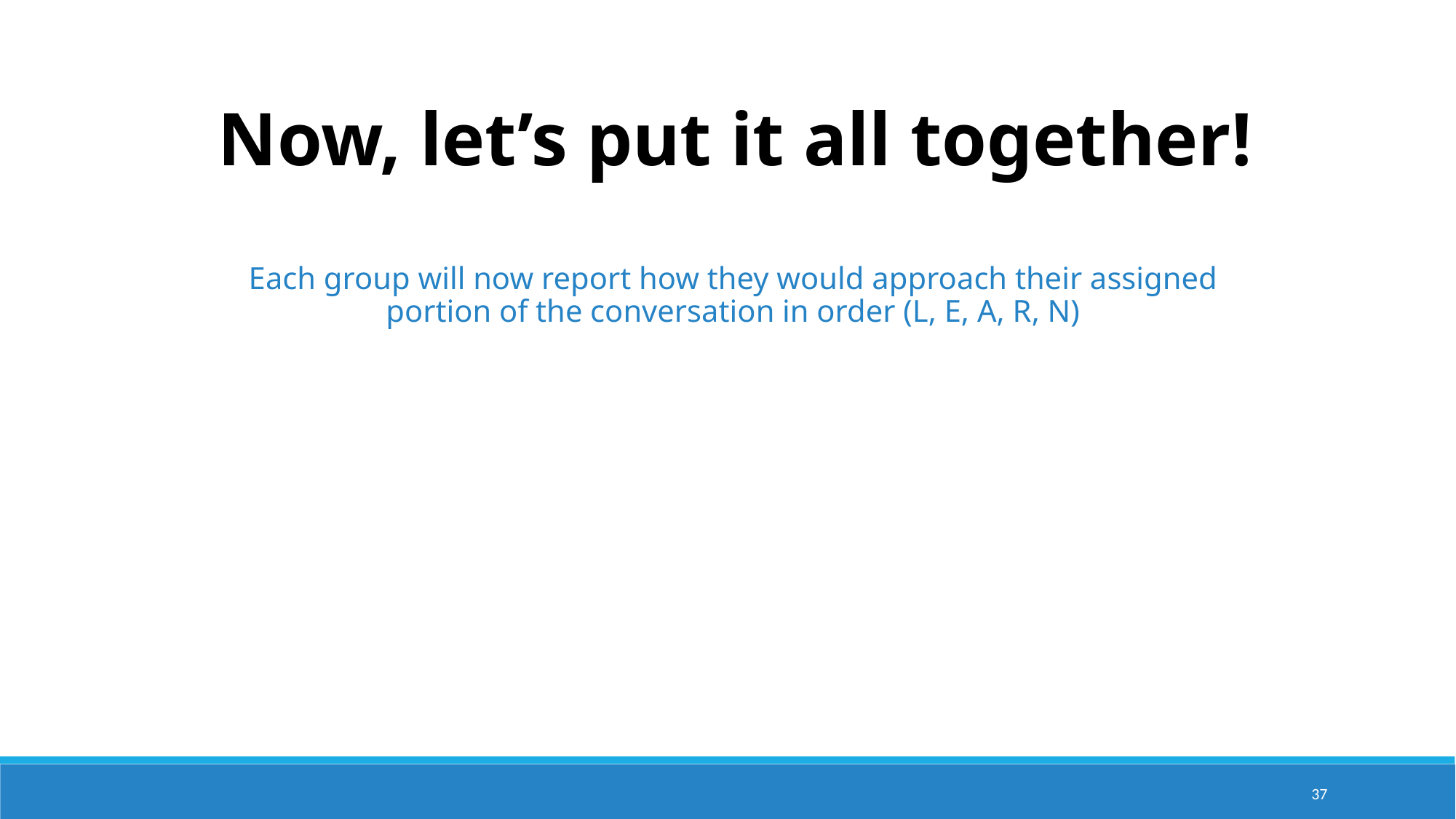

Now, let’s put it all together!
Each group will now report how they would approach their assigned portion of the conversation in order (L, E, A, R, N)
37

## Slide 38
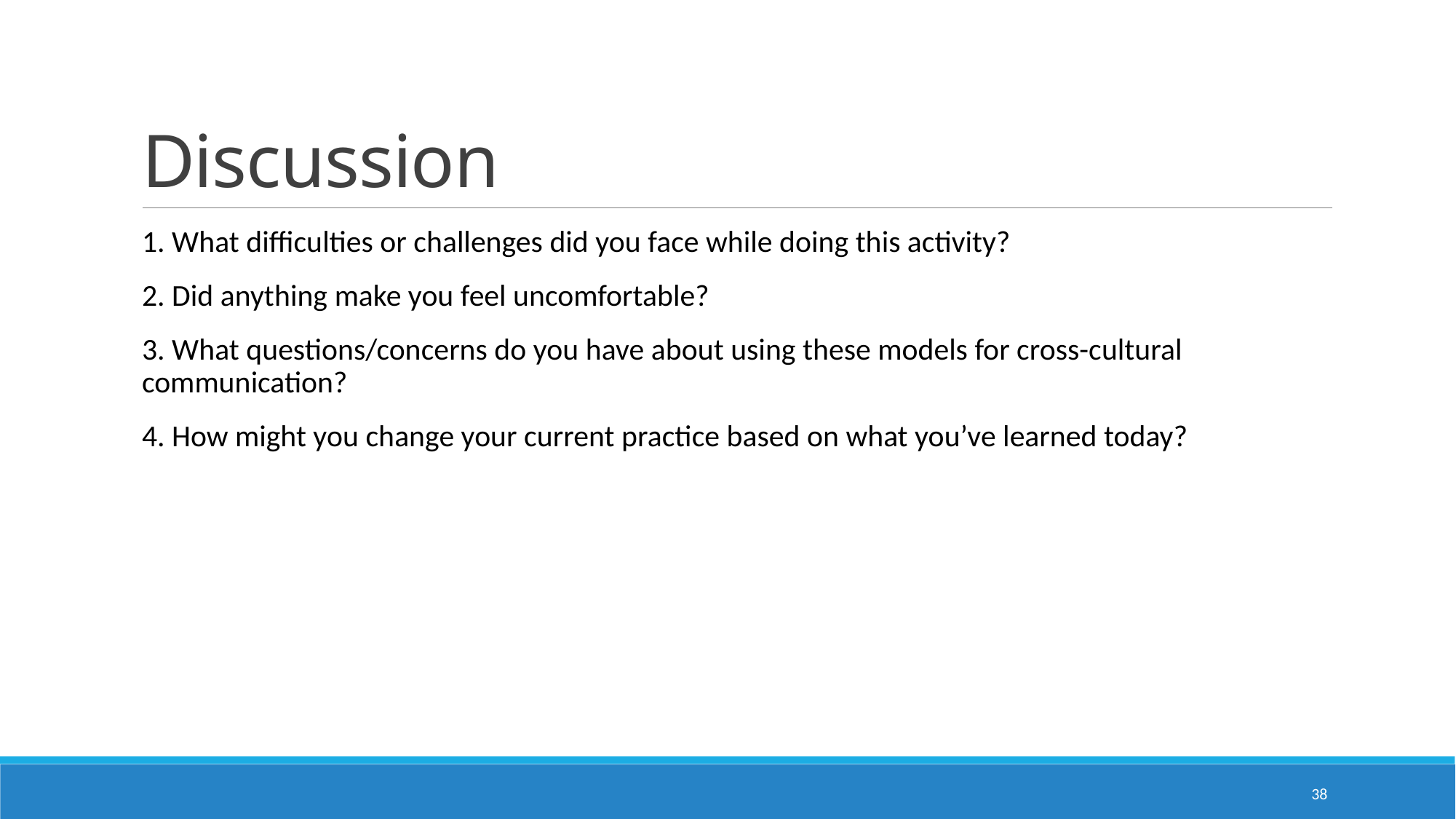

# Discussion
1. What difficulties or challenges did you face while doing this activity?​
2. Did anything make you feel uncomfortable?​
3. What questions/concerns do you have about using these models for cross-cultural communication?​
4. How might you change your current practice based on what you’ve learned today?​
​
38

## Slide 39
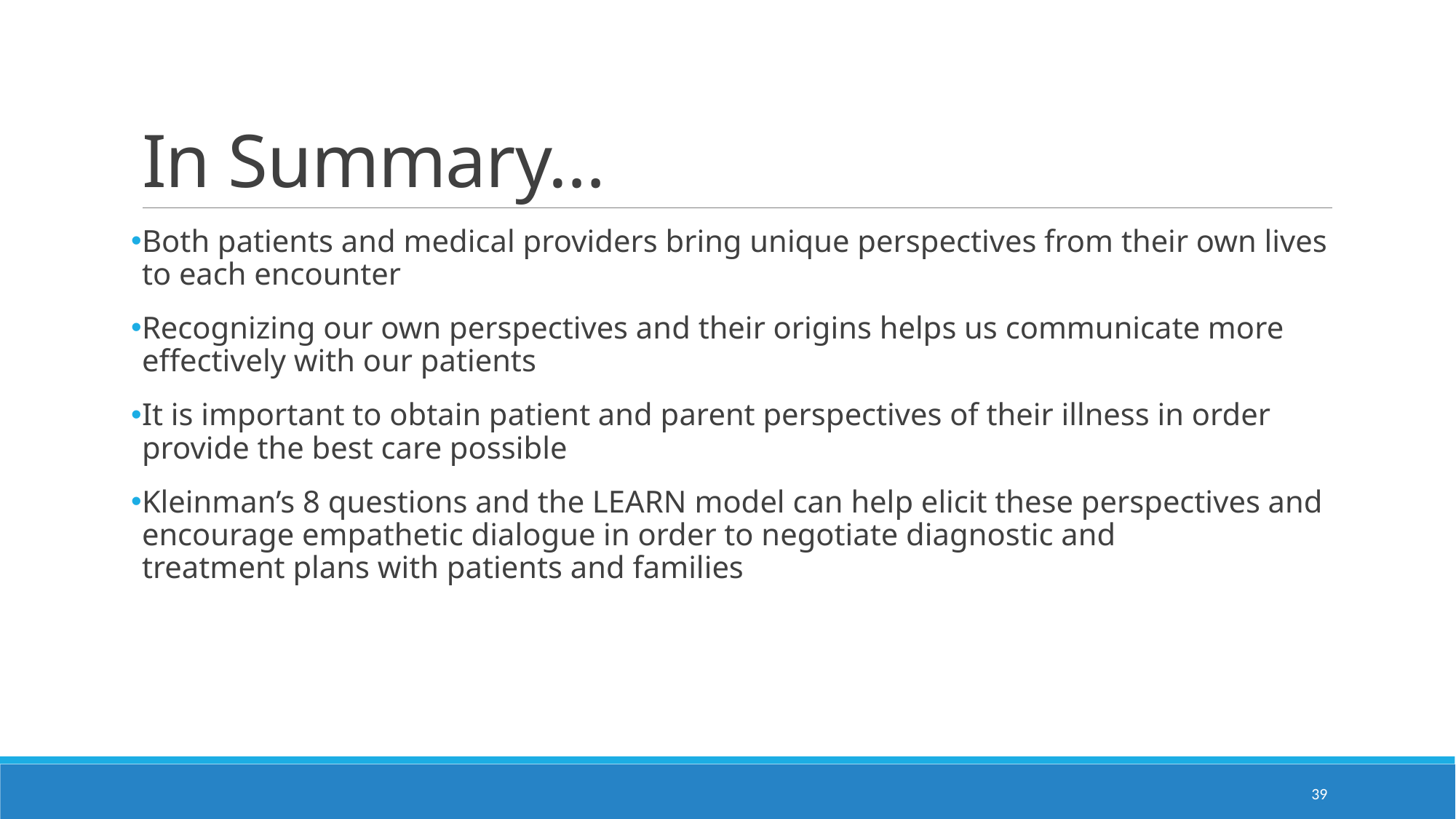

# In Summary…
Both patients and medical providers bring unique perspectives from their own lives to each encounter​
Recognizing our own perspectives and their origins helps us communicate more effectively with our patients​
It is important to obtain patient and parent perspectives of their illness in order provide the best care possible​
Kleinman’s 8 questions and the LEARN model can help elicit these perspectives and encourage empathetic dialogue in order to negotiate diagnostic and treatment plans with patients and families
​
39

## Slide 40
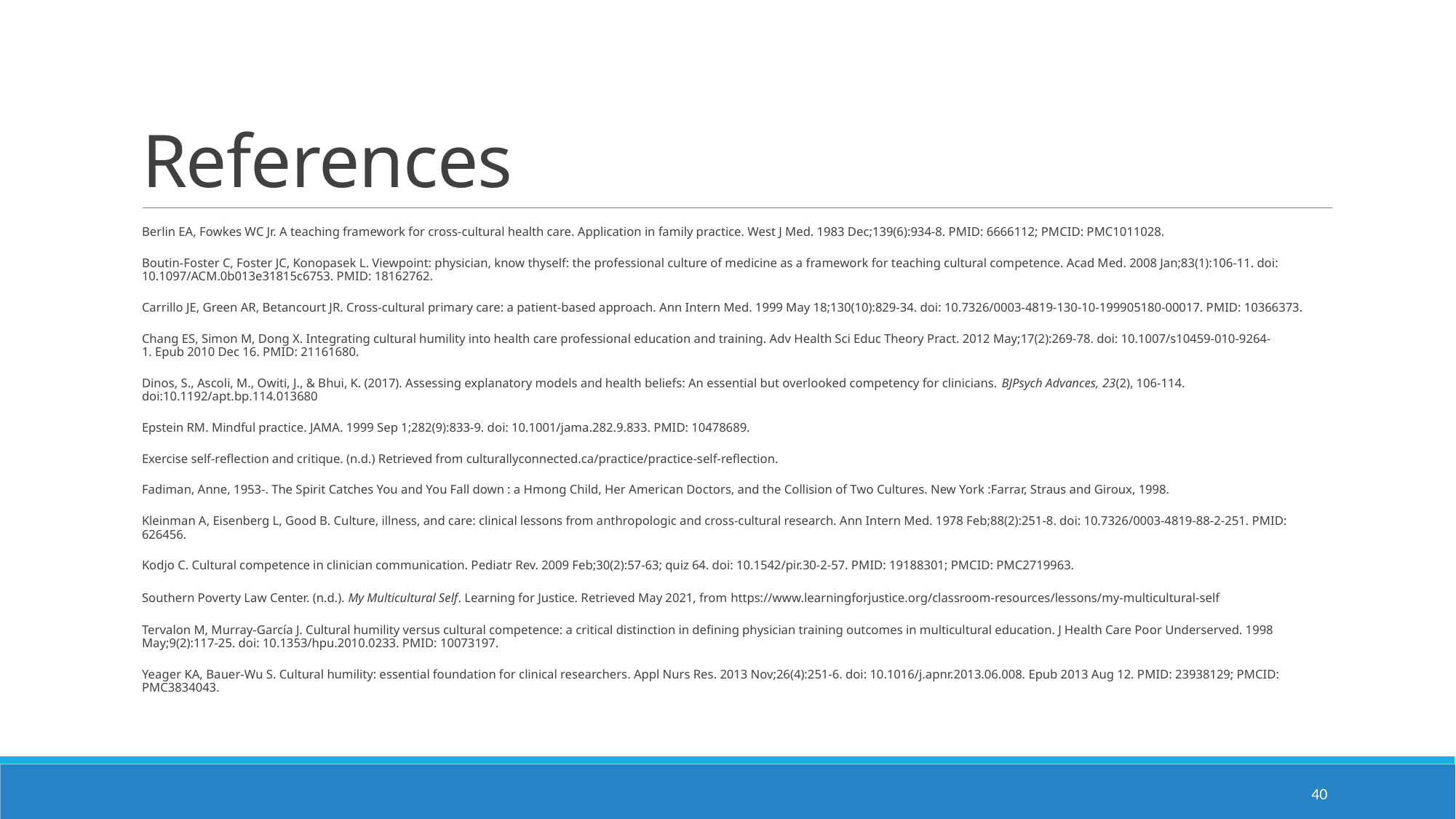

# References
Berlin EA, Fowkes WC Jr. A teaching framework for cross-cultural health care. Application in family practice. West J Med. 1983 Dec;139(6):934-8. PMID: 6666112; PMCID: PMC1011028.​
Boutin-Foster C, Foster JC, Konopasek L. Viewpoint: physician, know thyself: the professional culture of medicine as a framework for teaching cultural competence. Acad Med. 2008 Jan;83(1):106-11. doi: 10.1097/ACM.0b013e31815c6753. PMID: 18162762.​
Carrillo JE, Green AR, Betancourt JR. Cross-cultural primary care: a patient-based approach. Ann Intern Med. 1999 May 18;130(10):829-34. doi: 10.7326/0003-4819-130-10-199905180-00017. PMID: 10366373.​
Chang ES, Simon M, Dong X. Integrating cultural humility into health care professional education and training. Adv Health Sci Educ Theory Pract. 2012 May;17(2):269-78. doi: 10.1007/s10459-010-9264-1. Epub 2010 Dec 16. PMID: 21161680.​
Dinos, S., Ascoli, M., Owiti, J., & Bhui, K. (2017). Assessing explanatory models and health beliefs: An essential but overlooked competency for clinicians. BJPsych Advances, 23(2), 106-114. doi:10.1192/apt.bp.114.013680​
Epstein RM. Mindful practice. JAMA. 1999 Sep 1;282(9):833-9. doi: 10.1001/jama.282.9.833. PMID: 10478689.​
Exercise self-reflection and critique. (n.d.) Retrieved from culturallyconnected.ca/practice/practice-self-reflection.​
Fadiman, Anne, 1953-. The Spirit Catches You and You Fall down : a Hmong Child, Her American Doctors, and the Collision of Two Cultures. New York :Farrar, Straus and Giroux, 1998.​
Kleinman A, Eisenberg L, Good B. Culture, illness, and care: clinical lessons from anthropologic and cross-cultural research. Ann Intern Med. 1978 Feb;88(2):251-8. doi: 10.7326/0003-4819-88-2-251. PMID: 626456.​
Kodjo C. Cultural competence in clinician communication. Pediatr Rev. 2009 Feb;30(2):57-63; quiz 64. doi: 10.1542/pir.30-2-57. PMID: 19188301; PMCID: PMC2719963.​
Southern Poverty Law Center. (n.d.). My Multicultural Self. Learning for Justice. Retrieved May 2021, from https://www.learningforjustice.org/classroom-resources/lessons/my-multicultural-self
Tervalon M, Murray-García J. Cultural humility versus cultural competence: a critical distinction in defining physician training outcomes in multicultural education. J Health Care Poor Underserved. 1998 May;9(2):117-25. doi: 10.1353/hpu.2010.0233. PMID: 10073197.​
Yeager KA, Bauer-Wu S. Cultural humility: essential foundation for clinical researchers. Appl Nurs Res. 2013 Nov;26(4):251-6. doi: 10.1016/j.apnr.2013.06.008. Epub 2013 Aug 12. PMID: 23938129; PMCID: PMC3834043.​
​
40
